# Biscembranoids and Cembranoids from the Soft Coral *Sarcophyton elegans*

**DOI:** 10.3390/md15040085

**Published:** 2017-03-23

**Authors:** Wei Li, Yi-Hong Zou, Man-Xi Ge, Lan-Lan Lou, Yun-Shao Xu, Abrar Ahmed, Yun-Yun Chen, Jun-Sheng Zhang, Gui-Hua Tang, Sheng Yin

**Affiliations:** School of Pharmaceutical Sciences, Sun Yat-sen University, Guangzhou 510006, China; liwei249@mail2.sysu.edu.cn (W.L.); zouyh5@mail2.sysu.edu.cn (Y.-H.Z.); mintabby@outlook.com (M.-X.G.); loulanlan1006@163.com (L.-L.L.); xuysh6@mail2.sysu.edu.cn (Y.-S.X.); abrarpharma@gmail.com (A.A.); chenyy@mail.sysu.edu.cn (Y.-Y.C.); zhangjsh0814@163.com (J.-S.Z.); tanggh5@mail.sysu.edu.cn (G.-H.T.)

**Keywords:** *Sarcophyton elegans*, biscembranoids, cembranoids, NO inhibition

## Abstract

Two novel biscembranoids, sarelengans A and B (**1** and **2**), five new cembranoids, sarelengans C–G (**3**–**7**), along with two known cembranoids (**8** and **9**) were isolated from the South China Sea soft coral *Sarcophyton elegans*. Their structures were determined by spectroscopic and chemical methods, and those of **1**, **4**, **5**, and **6** were confirmed by single crystal X-ray diffraction. Compounds **1** and **2** represent the first example of biscembranoids featuring a *trans*-fused A/B-ring conjunction between the two cembranoid units. Their unique structures may shed light on an unusual biosynthetic pathway involving a cembranoid-∆^8^ rather than the normal cembranoid-∆^1^ unit in the *endo*-Diels-Alder cycloaddition. Compounds **2** and **3** exhibited potential inhibitory effects on nitric oxide production in RAW 264.7 macrophages, with IC_50_ values being at 18.2 and 32.5 μM, respectively.

## 1. Introduction

Biscembranoids are a group of unique tetraterpenoids exclusively occurring in the soft corals of the genera of *Sarcophyton*, *Sinularia*, and *Lobophytum* (family Alcyoniidae) [1,2,3,4,5]. Biosynthetically they are proposed to be derived from the Diels-Alder cycloaddition between two cembranoid monomers, forming a 14/6/14 (A/B/C) carbon ring system [6,7,8,9,10]. Since the first biscembranoid named methyl isosartortuoate was reported from the Chinese soft coral *Sarcophyton tortuosum* [1], more than 80 biscembranoids have been isolated so far [11]. The structural diversity of these biscembranoids mainly arise from the oxygenation and cyclization of the cembranoid-diene part (ring C), with maintaining of the basic structural features of cembranoid-dienophile moiety (ring A). Due to their significant biological activities (e.g., cytotoxic [2] and antibacterial [8] properties) and appealing architectures, biscembranoids have attracted broad interests from both natural product [12,13] and synthetic chemists [14,15] over the last decades.

*Sarcophyton elegans* is a common soft coral species found on the sea shore of the South China Sea. Previous chemical investigations of *S. elegans* have led to the isolation of several cembranoids, tetracyclic diterpenoids, steroids, and biscembranoids, some of which showed antibacterial and cytotoxic activities [8,16,17,18]. In our early work aiming at the discovery of antitumor agents from this species, two cembranoids with anti-tumor cell migration properties were isolated [19]. In our screening program aimed at the discovery of novel nitric oxide (NO) inhibitors from natural resources [20,21], the EtOAc fraction of the ethanolic extract of *S. elegans* showed a certain inhibitory activity against the lipopolysaccharide (LPS)-induced NO production in RAW 264.7 macrophages. Subsequent chemical investigation led to the isolation of two new biscembranoids (**1** and **2**), five new cembranoids (**3**–**7**), and two known compounds (**8** and **9**). Compounds **1** and **2** represent the first example of A/B ring *trans*-fused biscembranoids. Bioassay verified that compounds **2** and **3** were responsible for the NO inhibitory activities of the EtOAc fraction, with IC_50_ values being 18.2 and 32.5 μM, respectively. Herein, details of the isolation, structural elucidation, and NO inhibitory activities of these compounds are described.

## 2. Results and Discussion

**Figure 1:**
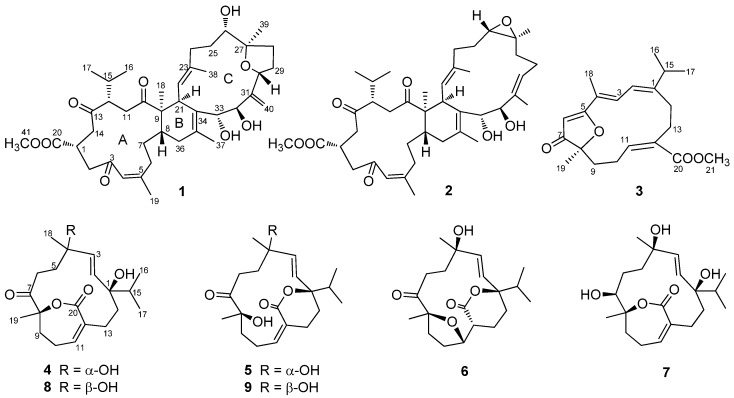
Structures of compounds **1**–**9**.

The frozen sample of *S. elegans* was chopped and exhaustively extracted with 95% EtOH at room temperature (rt). After removal of solvent in vacuo, the residue was suspended in H_2_O and then partitioned sequentially with petroleum ether (PE) and EtOAc. Various column chromatographic separations of the EtOAc extract afforded compounds **1**–**9** (Figure 1).

Compound **1**, a colorless crystal, had the molecular formula C_41_H_60_O_9_, as established by HRESIMS at *m*/*z* 719.4121 [M + Na]^+^ (calcd. 719.4130), corresponding to 12 degrees of unsaturation (DOUs). The IR absorption bands at 3408 and 1710 cm^−1^ indicated the presence of the hydroxyl and carbonyl groups, respectively. Detailed analysis of the ^1^H-NMR revealed the presence of seven methyl groups [δ_H_ 0.92 (3H × 2, d, *J* = 6.7 Hz), 1.07 (3H, s), 1.08 (3H, s), 1.54 (3H, s), 1.70 (3H, s), and 1.86 (3H, d, *J* = 1.2 Hz)], one methoxy group [δ_H_ 3.63 (3H, s)], four oxymethine protons [δ_H_ 3.47 (1H, m), 3.89 (1H, dd, *J* = 10.2, 4.2 Hz), 4.07 (1H, d, *J* = 9.7 Hz), and 4.39 (1H, d, *J* = 9.7 Hz)], a terminal double bond [δ_H_ 5.21 (1H, s) and 5.47 (1H, s)], two olefinic protons [δ_H_ 4.88 (1H, d, *J* = 10.6 Hz) and 5.96 (1H, d, *J* = 1.2 Hz)], and a series of aliphatic methylene or methine multiplets. The ^13^C-NMR spectrum, in combination with DEPT experiments, resolved 41 carbon resonances attributable to three ketone groups (δ_C_ 215.5, 215.4 and 203.4), a methyl ester group (δ_C_ 176.3 and 52.3), a terminal double bond (δ_C_ 151.6 and 112.8), two trisubstituted double bonds (δ_C_ 154.1, 136.9, 128.2, and 127.4), a tetrasubstituted double bond (δ_C_ 132.7 and 130.2), two sp^3^ quaternary carbons (one oxygenated), nine sp^3^ methines (four oxygenated), 10 sp^3^ methylenes, and seven methyls. As eight of the 12 DOUs were accounted for by three ketones, an ester carbonyl group, and four double bonds, the remaining DOUs required that **1** was tetracyclic. The aforementioned data are characteristic of a biscembranoid, closely related to those reported in the literature [5,12,22].

**Figure 2:**
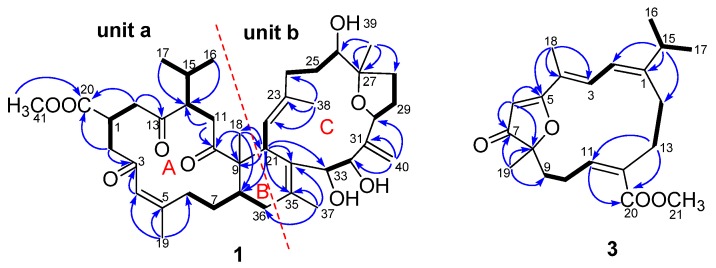
^1^H−^1^H COSY (correlation spectroscopy) (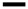) and key HMBC (heteronuclear multiple-bond correlation spectroscopy) (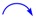) correlations of compounds **1** and **3**.

Detailed 2D NMR studies (HSQC, ^1^H–^1^H COSY, and HMBC experiments) further confirmed the presence of two highly oxygenated cembranoid units (**a** and **b**) in **1** (Figure 2). Three fragments, C-2−C-1−C-14, C-6−C-7−C-8, and C-11−C-12−C-15−C-16 (C-17), were first established in unit **a** by the ^1^H–^1^H COSY correlations. The connectivities of these fragments, three ketones, one double bond, one quaternary carbon, and one methyl ester were achieved by HMBC correlations of H_3_-18/C-8, C-9, and C-10, H_3_-19/C-4, C-5, and C-6, H_2_-2(H-4)/C-3, H_2_-11/C-10, and C-12, and H_2_-14/C-13, and C-20, which generated a methyl sarcoate moiety (ring A) commonly found in biscembranoids [23].

As for unit **b**, four spin systems of C-21–C-22, C-24–C-25–C-26, C-28–C-29–C-30, and C-32–C-33 were readily recognized from the ^1^H–^1^H COSY spectrum. The connections of these fragments, three double bonds, and one sp^3^ quaternary carbon were achieved by HMBC correlations, generating the framework of a 14-membered carbon ring C. Although no HMBC correlation was observed between H-30 and C-27, the presence of the oxygen bridge between C-30 and C-27 were deduced by comparison of its ^13^C-NMR data with those of bisglaucumlide H [24] bearing the similar tetrahydrofuran rings, as well as on consideration of the unsaturation degrees of the molecule. The connection of units **a** and **b** was finally achieved by HMBC correlations of H_3_-18/C-8, C-9, and C-21, H-21/C-8, C-9, C-18, and C-35, and H_3_-37/C-34, C-35, and C-36, which formed a six-membered carbon ring B bridging these two parts.

**Figure 3:**
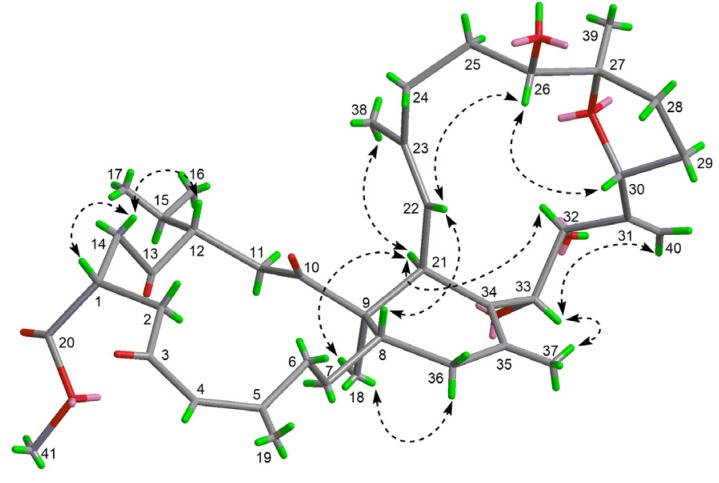
Key NOE (nuclear overhauser effect) correlations of **1**.

**Figure 4:**
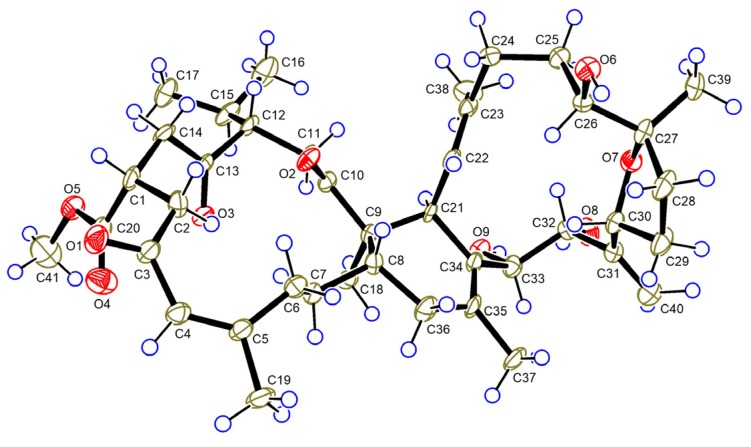
ORTEP diagram of **1**.

The relative configuration of **1** was established based on a NOESY experiment and analysis of coupling constants. The strong NOE interactions of H-8/H-22 and H_3_-18/H_2_-36b indicated that H-8 and CH_3_-18 occupied the axial bonds of ring B in a chair conformation (Figure 3). Thus, ring A and B were *trans*-fused. The NOE correlations of H-14a/H-1 and H-12 indicated that these protons were cofacial on ring A and were arbitrarily assigned as β-orientation. The NOE correlations of H-22/H-8 and H-26 and H-26/H-30 suggested that these protons were cofacial on ring C and were assigned as β-orientation, while correlations of H-21/H_3_-18 and H-32 suggested that these protons were α-oriented. The *trans-*relationship of H-33 and H-32 was implied by the large coupling constant between these two protons (*J* = 9.7 Hz) [25]. Finally, the structure of **1** including the absolute configuration was confirmed by a single crystal X-ray crystallographic analysis using anomalous scattering of Cu Kα radiation, Flack parameter = 0.04 (13) (Figure 4). Thus, compound **1** was given a trivial name sarelengan A.

Compound **2**, a colorless oil, had the molecular formula C_41_H_60_O_8_, as determined by HRESIMS ion at *m/z* 703.4193 [M + Na]^+^ (calcd. 703.4180). The 1D NMR spectra of **2** exhibited most of the structural features found in **1**, with major difference being the absence of a tetrahydrofuran ring and the replacement of a terminal double bond by a trisubstituted double bond in **2**. The relatively upfield-shifted carbon signals of an oxymethine (δ_C_ 64.0, C-26) and a quaternary carbon (δ_C_ 61.2, C-27) suggested the presence of an epoxy ring in **2** [26]. The epoxy ring was located at C-26 and C-27 by HMBC correlations of H_3_-39/C-26 and C-27, as well as ^1^H–^1^H COSY correlation of H_2_-25/H-26. The trisubstituted double bond was located at C-30 and C-31 by HMBC correlations from H_3_-40 to C-30 and C-31, and ^1^H–^1^H COSY correlation of H_2_-29/H-30. The planar structure of **2** was further confirmed by detailed analyses of its 2D NMR data. The configuration of **2** was assigned to be the same as that of **1** by comparison of their NOESY and ^13^C-NMR data. In particular, the NOESY correlations of H-8/H-22 and H_3_-18/H-36b confirmed the *trans*-fused A/B ring, while the *trans*-relationship of H-32 and H-33 was assigned by NOE correlations of H-33/H_3_-37 and H-32/H-21, as well as the large coupling constant (*J* = 10.0 Hz) between H-32 and H-33. The NOE correlations of H-22/H-8 and H-26/H-22 and H-28b indicated that H-26 was β-oriented and in a *trans*-position of CH_3_-39 on the epoxy ring (S56, Supplementary Materials), which was further confirmed by comparison of the NMR data of C-26 and C-27 in **2** with those of known analogue sarcophytolide H [13]. The *E* geometry of Δ^30^ was assigned by NOE correlation of H-30/H-32. Thus, compound **2** was determined as depicted and was given the trivial name sarelengan B.

**Scheme 1:**
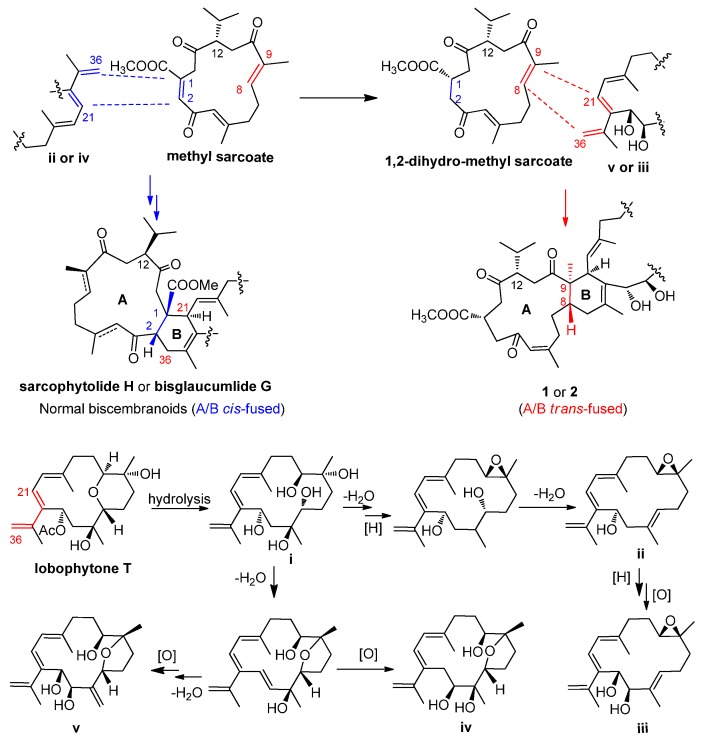
Proposed biogenesis of compounds **1** and **2**.

It is worth noting that compounds **1** and **2** possessed a *trans*-fused A/B ring system, differing from those of the previously reported biscembranoids [8,10], suggesting that the coupling between two monemeric cembranoids in **1** and **2** may involve an unusual biosynthetic pathway. As shown in Scheme 1, the well-known soft coral metabolite, methyl sarcoate, was considered as the cembranoid-dienophile precursor, which underwent biohydrogenation to produce a dienophile intermediate 1,2-dihydro-methyl sarcoate. Lobophytone T isolated from *Lobophytum pauciflorum* [22], was served as the cembranoid-diene precursor, which underwent hydrolysis to generate intermediate **i**. Dehydration of **i** followed by a series redox reactions generated the epoxy intermediates (**ii** and **iii**) and the tetrahydrofuran-containing intermidiates (**iv** and **v**), respectively. The *endo*-Diels-Alder cycloaddition between methyl sarcoate-Δ^1^ and **ii**- or **iv**-Δ^21(34)^, Δ^35^ generates the “normal biscembranoids” (A/B ring *cis*-fused), sarcophytolide H [13] or bisglaucumlide G [24], respectively, while the *endo*-Diels-Alder cycloaddition between methyl 1,2-dihydro-methyl sarcoate -Δ^8^ and **v-** or **iii**-Δ^21(34)^, Δ^35^ produces the A/B ring *trans*-fused scaffold of **1** or **2**, respectively.

Compound **3**, a colorless oil, had the molecular formula C_21_H_28_O_4_ as determined by HRESIMS at *m*/*z* 367.1871 [M + Na]^+^ (calcd. 367.1880), implying eight DOUs. The ^1^H-NMR data of **3** showed two methyl groups [δ_H_ 1.43 (3H, s) and 1.96 (3H, d, *J* = 1.1 Hz)], an isopropyl group [δ_H_ 1.12 (3H × 2, d, *J* = 6.8 Hz), 2.52 (1H, m)], four olefinic protons [δ_H_ 5.64 (1H, s), 5.99 (1H, t, *J* = 6.4 Hz), 6.29 (1H, d, *J* = 12.1 Hz), and 7.42 (1H, d, *J* = 12.1, 1.1 Hz)], and a series of aliphatic methylene or methine multiplets. The ^13^C-NMR spectrum, in combination with DEPT experiments, resolved 21 carbon resonances attributable to one ketone group (δ_C_ 209.6), a methyl ester group (δ_C_ 169.2 and 51.5), four trisubstituted double bonds (δ_C_ 187.7, 164.1, 152.1, 136.6, 130.1, 121.7, 119.3, and 100.4), one sp^3^ oxygenated quaternary carbon, one sp^3^ methine, four sp^3^ methylenes and four methyls. Aforementioned information indicated that **3** possessed structural features of a cembranoid. The gross structure of **3** was further established by analysis of its 2D NMR data (Figure 2). In the ^1^H–^1^H COSY spectrum four partial structures, C-2–C-3, C-9–C-10–C-11, C-13–C-14, and C-16–C-15–C-17 were established by a series of ^1^H–^1^H COSY correlation (Figure 2). The connectivities of the four structural fragments, the double bonds, the methyls, and the methyl ester were achieved by analysis of the HMBC correlations. In particular, HMBC correlations from H-15 to C-1, C-2, and C-14, from H_3_-18 to C-3, C-4, and C-5, from H_3_-19 to C-7, C-8, and C-9, from H-6 to C-5, C-7, and C-8, and from H_2_-13 to C-11 and C-20 constructed a 14-membered carbon ring. As the above-mentioned structure elucidation already accounted for seven of the eight DOUs, the remaining one DOU required the presence of an additional ring. The severely down-field shifted carbon signals of C-5 (δ_C_ 187.7) and C-8 (δ_C_ 91.3) implied the presence of an ether bond between C-8 and C-5 to form a furan-3(2*H*)-one ring. This was further supported by comparison of its ^13^C-NMR data with those of synthetic 2,2,5-trimethyl-3(2*H*)-furanone [27]. The configuration of C-8 was tentatively assigned as *S* based on the biogenetic consideration of this compound class, while the 1*E*, 3*E*, 5*Z*, 11*Z* configurations were determined by NOESY correlations of H-2/H_3_-16, H-2/H_3_-18, H-6/H_3_-18, and H-11/H_2_-13, respectively. Thus, compound **3** was determined as shown in Figure 1 and was given the trivial name sarelengan C.

**Figure 5:**
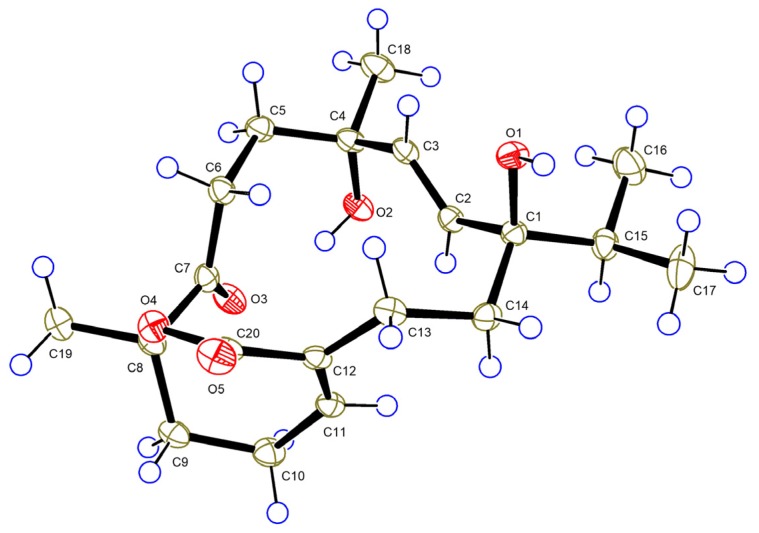
ORTEP diagram of **4**.

Compound **4**, a colorless crystal, had the molecular formula C_20_H_30_O_5_, as established by HRESIMS. The NMR data of **4** were very similar to those of sartrolide E (**8**) [28], with slight differences arising from the carbon signals of C-5 (δ_C_ 34.3 in **4**; δ_C_ 32.8 in **8**) and C-18 (δ_C_ 31.4 in **4**; δ_C_ 29.9 in **8**), indicating that **4** was a C-4 epimer of **8**. Detailed 2D analysis further supported that they shared the same planar structure. The structure of **4** including the absolute configuration (1*R*, 4*S*, and 8*R*) was secured by a single crystal X-ray crystallographic analysis using anomalous scattering of Cu Kα radiation, Flack parameter = 0.08 (10) (Figure 5). Thus, compound **4** was given the trivial name sarelengan D.

**Figure 6:**
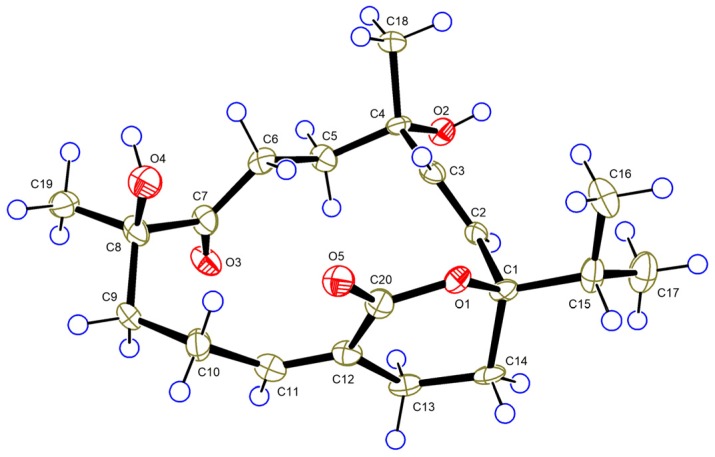
ORTEP diagram of **5**.

Compound **5**, a colorless crystal, gave a [M + Na]^+^ ion at *m*/*z* 373.1984 in the HRESIMS corresponding to the molecular formula C_20_H_30_O_5_. The 1D NMR spectra of **5** showed high similarity to those of sarcophelegan B (**9**) [19], except for the slight difference of carbon signals of C-5 (δ_C_ 38.1 in **5**; δ_C_ 36.5 in **9**) and C-18 (δ_C_ 26.9 in **5**; δ_C_ 29.3 in **9**), indicating that **5** was a C-4 epimer of **9**. Detailed 2D analysis further supported that they shared the same planar structure. The structure of **5** including the absolute configuration (1*R*, 4*S*, and 8*R*) was secured by a single crystal X-ray crystallographic analysis using anomalous scattering of Cu Kα radiation, Flack parameter = −0.01 (10) (Figure 6). Thus, compound **5** was given the trivial name sarelengan E.

**Figure 7:**
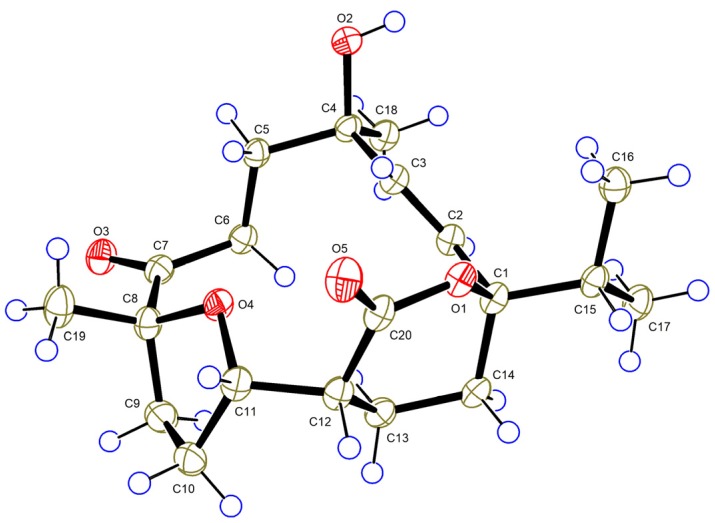
ORTEP diagram of **6**.

Compound **6**, a colorless crystal, exhibited a molecular formula of C_20_H_30_O_5_ as determined by HRESIMS. The ^1^H and ^13^C-NMR data of **6** showed high similarity to those of sarcophelegan B (**9**) [19], except for the replacement of the Δ^11^ in **9** by an oxymethine (δ_H_ 4.87; δ_C_ 80.8) and a methine (δ_H_ 2.69; δ_C_ 44.1) in **6**. Since **6** possessed the same DOUs as that of **9**, the formation of an oxygen bridge between C-8 and the oxymethine (C-11) was suggested. This was supported by the downfield-shifted carbonyl at C-20 (δ_C_ 175.3 in **6**; δ_C_ 168.8 in **9**) and C-8 (δ_C_ 90.0 in **6**; δ_C_ 79.7 in **9**), although no direct HMBC correlation from H-11 to C-8 was available. The structure of **6** was further confirmed by converting **9** to **6** in a basic condition and the absolute configuration (1*S*, 4*R*, 8*R*, 11*S*, and 12*R*) was assigned by a single crystal X-ray diffraction analysis with Flack parameter = −0.05 (13) (Figure 7). Thus, compound **6** was given a trivial name sarelengan F.

Compound **7** had a molecular formula of C_20_H_32_O_5_ as established by HRESIMS data. The 1D NMR data of **7** were very similar to those of sartrolide D [28], a known cembranoid isolated from *Sarcophyton trocheliophorum*, with the only difference being due to the chemical shift of C-7 (δ_C_ 66.1 in **7**; δ_C_ 72.7 in sartrolide D), indicating that **7** was a C-7 epimer of sartrolide D. Detailed 2D analysis further supported that they shared the same planar structure. The β-orientation of 7-OH was supported by the NOE correlations of H-2/H-7 and H-15. Thus, compound **7** was given the trivial name sarelengan G.

The known compounds, sartrolide E (**8**) [28] and sarcophelegan B (**9**) [19], were identified by comparison of their NMR data and optical rotation values with those reported in the literature.

All compounds were evaluated for their inhibitory effects on the NO production in LPS-induced RAW 264.7 macrophages using the Griess assay, and quercetin was used as the positive control (IC_50_ = 20.0 μM). Compounds **2** and **3** showed moderate inhibitory activities with IC_50_ values being at 18.2 and 32.5 μM, respectively, while other compounds were inactive (inhibition <50% at 50 μM). To investigate whether the NO inhibition was caused by the cytotoxicity, the effects of compounds **2** and **3** on LPS-induced RAW 264.7 macrophages viability were measured using the MTT method. The results showed that **2** and **3** (up to 80 μM) had no significant cytotoxicity towards the cells in 24 h incubation.

## 3. Materials and Methods

### 3.1. General Experimental Procedures

X-ray data were collected using an Agilent Xcalibur Nova X-ray diffractometer (Agilent, Santa Clara, CA, USA). Melting points were measured on an X-4 melting instrument (SRI International, Silicon Valley, CA, USA) and are uncorrected. Optical rotations were measured on a Rudolph Autopol I automatic polarimeter (Perkin-Elmer, Waltham, MA, USA), UV spectra on a Shimadzu UV-2450 spectrophotometer (Shimadzu, Kyoto, Japan), and IR spectra were determined on a Bruker Tensor 37 infrared spectrophotometer (Bruker, Karlsruhe, Germany). NMR spectra were measured on a Bruker AM-400 spectrometer (Bruker, Karlsruhe, Germany) at 25 °C. ESIMS was measured on a Finnigan LCQ Decainstrument (Thermo Finnigan, San Jose, CA, USA), and HRESIMS were performed on a Waters Micromass Q-TOF spectrometer (Waters, Milford, MA, USA). A Shimadzu LC-20 AT equipped with an SPD-M20A PDA detector (Shimadzu, Kyoto, Japan) was used for HPLC. A YMC-pack ODS-A column (250 × 10 mm, S-5 μm, 12 nm) (YMC, Tokyo, Japan) were used for semi-preparative HPLC separation. Silica gel (300–400 mesh, Qingdao Haiyang Chemical Co., Ltd., Qingdao, China), C_18_ reversed-phase (RP-C_18_) silica gel (12 nm, S-50 μm, YMC Co., Ltd., Kyoto, Japan), and Sephadex LH-20 gel (Amersham Biosciences, Piscataway, NJ, USA) were used for column chromatography (CC). All solvents were of analytical grade (Guangzhou Chemical Reagents Company, Ltd., Guangzhou, China).

### 3.2. Animal Material

The soft coral *S*. *elegans* were collected from the Xisha Islands in the South China Sea, in October 2014, at a depth of 8–10 m of water. The biological material was frozen immediately until used and was identified by Cheng-Qi Fan from East China Sea Fisheries Research Institute. A voucher specimen (accession number: HLRZ201410) has been deposited at the School of Pharmaceutical Sciences, Sun Yat-sen University, Guangzhou, China.

### 3.3. Extraction and Isolation

The frozen samples (500 g, wet weight) of *S*. *elegans* were extracted with EtOH (3 × 2 L) at rt to give 40 g of crude extract. The extract was suspended in H_2_O (200 mL) and partitioned sequentially to give dried PE (5 g) and EtOAc (9 g) extracts. The EtOAc extract was subjected to silica gel CC eluted with a CH_2_Cl_2_/MeOH gradient (200:1→20:1) to afford Fr. I–VI. Separation of the Fr. II (1 g) by Sephadex LH-20 eluted with MeOH led to Fr. IIa−IIb. Fr. IIa was purified on a semi-preparative reversed-phase (RP) HPLC system equipped with a YMC column (CH_3_CN/H_2_O, 8:2, 3 mL/min) to give **3** (4 mg, *t*_R_ 10 min). Fr. IIb was purified using RP-HPLC (CH_3_CN/H_2_O, 7:3, 3 mL/min) to give **5** (11 mg, *t*_R_ 8 min). Fractionation of Fr. III (2.5 g) by Sephadex LH-20 eluted with EtOH led to Fr. IIIa−IIIc. Fr. IIIa (1 g) was chromatographed over a RP-C_18_ column eluted with MeOH/H_2_O (4:6→10:0) to afford Fr. IIIa1−IIIa3. Fr. IIIa1 was loaded onto a Sephadex LH-20 column and eluted with CH_2_Cl_2_-MeOH (1:1) to give **6** (20 mg). Fr. IIIa2 was purified using RP-HPLC (CH_3_CN/H_2_O, 6:4, 3 mL/min) to give **8** (31 mg, *t*_R_ 8.5 min) and **9** (16.8 mg, *t*_R_ 9 min). Fr. VI (2 g) was subjected to silica gel CC (PE/EtOAc, 2:1→1:4) to give Fr. VIa–VId. Fr. VIa was purified using RP-HPLC (CH_3_CN/H_2_O, 4:6, 3 mL/min) to give **4** (35 mg, *t*_R_ 10 min) and **7** (4 mg, *t*_R_ 12 min). Fr. VIc was purified using RP-HPLC (MeOH/H_2_O, 8:2, 3 mL/min) to give **1** (3 mg, *t*_R_ 10.5 min). Fr. VId was purified using RP-HPLC (MeOH/H_2_O, 7:3, 3 mL/min) to give **2** (12 mg, *t*_R_ 11 min).

Sarelengan A (**1**). Colorless crystal; mp 210–212 °C; ${\left[\alpha \right]}_{\text{D}}^{25}$ +130.0 (*c* 0.20, MeOH); UV (MeOH) λ_max_ (log ε) 217 (4.05) nm; IR (KBr) ν_max_ 3408, 2968, 2931, 1710, 1698, 1379, 1190, 987, 734 cm^−1^; ^1^H and ^13^C-NMR data, see Table 1; HRESIMS *m*/*z* 719.4121 [M + Na]^+^ (calcd. for C_41_H_60_O_9_Na, 719.4130).

Crystal data for sarelengan A (**1**). C_4__1_H_6__0_O_9_·C_2_H_3_N, (M = 737.98 g/mol): monoclinic, space group P2_1_ (no. 4), *a* = 13.28203 (9) Å, *b* = 9.73686(7) Å, *c* = 16.15823 (13) Å, β = 100.9378 (7)°, *V* = 2051.70 (3) Å^3^, *Z* = 2, *T* = 103 (2) K, μ (Cu Kα) = 0.664 mm^−1^, *Dcalc* = 1.1945 g/cm^3^, 35174 reflections measured (5.58° ≤ 2θ ≤ 144.78°), 7856 unique (*R*_int_ = 0.0889, *R*_sigma_ = 0.0626) which were used in all calculations. The final *R*_1_ was 0.0557 (I ≥ 2σ (I)) and wR_2_ was 0.1463 (all data). Flack parameter = 0.04 (13). Crystallographic data for the structure of **1** have been deposited in the Cambridge Crystallographic Data Centre (deposition number: CCDC 1520904).

Sarelengan B (**2**). Colorless oil; ${\left[\alpha \right]}_{\text{D}}^{25}$ +154.7 (*c* 0.30, MeOH); UV (MeOH) λ_max_ (log ε) 209 (3.97) nm; IR (KBr) ν_max_ 3408, 2928, 1736, 1698, 1438, 1373, 1284, 1037, 735 cm^−1^; ^1^H and ^13^C-NMR data, see Table 1; HRESIMS *m*/*z* 703.4193 [M + Na]^+^ (calcd. for C_41_H_60_O_8_ Na, 703.4180).

Sarelengan C (**3**). Colorless oil; ${\left[\alpha \right]}_{\text{D}}^{25}$ +110.5 (*c* 0.40, MeOH); UV (MeOH) λ_max_ (log ε) 222 (3.87), 354 (4.13) nm; IR (KBr) ν_max_ 2956, 2925, 1691, 1544, 1438, 1377, 1137, 794 cm^−1^; ^1^H and ^13^C-NMR data, see Table 2 and Table 3; HRESIMS *m*/*z* 367.1871 [M + Na]^+^ (calcd. for C_21_H_28_O_4_Na, 367.1880).

Sarelengan D (**4**). Colorless crystal; mp 186–188 °C; ${\left[\alpha \right]}_{\text{D}}^{25}$ +34.9 (*c* 0.75, MeOH); UV (MeOH) λ_max_ (log ε) 210 (3.67) nm; IR (KBr) ν_max_ 3398, 2927, 1697, 1437, 1377, 1283, 1206, 1035, 734 cm^−1^; ^1^H and ^13^C-NMR data, see Table 2 and Table 3; HRESIMS *m*/*z* 373.1979 [M + Na]^+^ (calcd. for C_20_H_30_O_5_Na, 373.1985).

Crystal data for sarelengan D (**4**). C_20_H_30_O_5_, (*M* = 350.46 g/mol): monoclinic, space group P2_1_ (no. 4), *a* = 7.06058 (10) Å, *b* = 8.04934 (10) Å, *c* = 16.4003 (2) Å, β = 90.0131 (11)°, *V* = 932.08 (2) Å^3^, *Z* = 2, *T* = N/A K, μ (Cu Kα) = 0.716 mm^−1^, *Dcalc* = 1.2486 g/cm^3^, 8797 reflections measured (13.66° ≤ 2θ ≤ 144.14°), 3369 unique (*R*_int_ = 0.0135, *R*_sigma_ = 0.0126) which were used in all calculations. The final *R*_1_ was 0.0254 (I ≥ 2σ (I)) and *wR*_2_ was 0.0645 (all data). Flack parameter = 0.08 (10). Crystallographic data for the structure of **4** have been deposited in the Cambridge Crystallographic Data Centre (deposition number: CCDC 1520903).

Sarelengan E (**5**). Colorless crystal; mp 177–178 °C; ${\left[\alpha \right]}_{\text{D}}^{25}$ −6.4 (*c* 0.90, MeOH); UV (MeOH) λ_max_ (log ε) 230 (3.66) nm; IR (KBr) ν_max_ 3418, 2965, 2928, 1685, 1616, 1450, 1177, 791 cm^−1^; ^1^H and ^13^C-NMR data, see Table 2 and Table 3; HRESIMS *m*/*z* 373.1984 [M + Na]^+^ (calcd. for C_20_H_30_O_5_Na, 373.1985).

Crystal data for sarelengan E (**5**). C_20_H_30_O_5_, (*M* = 350.46 g/mol): triclinic, space group P1 (no. 1), *a* = 9.6159 (2) Å, *b* = 9.7162 (2) Å, *c* = 21.2262 (4) Å, α = 102.7736 (18)°, β = 99.6112 (18)°, γ = 96.8219 (18)°, *V* = 1881.77 (7) Å^3^, *Z* = 4, *T* = N/A K, μ (Cu Kα) = 0.709 mm^−1^, *Dcalc* = 1.2369 g/cm^3^, 35900 reflections measured (4.36° ≤ 2θ ≤ 144.66°), 11571 unique (*R*_int_ = 0.0249, R_sigma_ = 0.0249) which were used in all calculations. The final *R*_1_ was 0.0372 (I ≥ 2σ (I)) and *wR*_2_ was 0.1183 (all data). Flack parameter = −0.01 (10). Crystallographic data for the structure of **5** have been deposited in the Cambridge Crystallographic Data Centre (deposition number: CCDC 1520902).

Sarelengan F (**6**). Colorless crystal; mp 183–185 °C; ${\left[\alpha \right]}_{\text{D}}^{25}$ +57.3 (*c* 0.67, MeOH); UV (MeOH) λ_max_ (log ε) 207 (3.03) nm; IR (KBr) ν_max_ 3533, 2979, 2930, 1717, 1695, 1462, 1270, 1027, 738 cm^−1^; ^1^H and ^13^C-NMR data, see Table 2 and Table 3; HRESIMS *m*/*z* 373.1985 [M + Na]^+^ (calcd. for C_20_H_30_O_5_Na, 373.1985).

Crystal data for sarelengan F (**6**). C_20_H_30_O_5_, (*M* = 350.46 g/mol): orthorhombic, space group P2_1_2_1_2_1_ (no. 19), *a* = 6.43129 (10) Å, *b* = 12.91548 (17) Å, *c* = 22.1013 (3) Å, *V* = 1835.81 (5) Å^3^, *Z* = 4, *T* = N/A K, μ (Cu Kα) = 0.727 mm^−1^, *Dcalc* = 1.2679 g/cm^3^, 17493 reflections measured (7.92° ≤ 2θ ≤ 144.66°), 3612 unique (*R*_int_ = 0.0661, *R*_sigma_ = 0.0432) which were used in all calculations. The final *R*_1_ was 0.0418 (I ≥ 2σ (I)) and *wR*_2_ was 0.1088 (all data). Flack parameter = −0.05 (13). Crystallographic data for the structure of **6** have been deposited in the Cambridge Crystallographic Data Centre (deposition number: CCDC 1520905).

Sarelengan G (**7**). Colorless oil; ${\left[\alpha \right]}_{\text{D}}^{25}$ +10.0 (*c* 0.20, MeOH); UV (MeOH) λ_max_ (log ε) 226 (3.70) nm; IR (KBr) ν_max_ 3454, 2925, 1673, 1388, 1225, 1124, 1050, 734 cm^−1^; ^1^H and ^13^C-NMR data, see Table 2 and Table 3; HRESIMS *m*/*z* 375.2138 [M + Na]^+^ (calcd. for C_20_H_32_O_5_Na, 375.2142).

### 3.4. Chemical Transformation of ***9*** to ***6***

Compound **9** (6 mg) was treated with NaOH (1% in MeOH, 1 mL) at room temperature for 2 h. The mixture was then diluted with 5 mL of H_2_O, followed by the extraction of EtOAc (3 × 5 mL). The organic layer was dried and evaporated to give a residue, which was purified on a flash silica gel column eluting with CH_2_Cl_2_ to afford **6** (3.4 mg). The structure was confirmed by comparison of its NMR and optical rotation data with those of the natural product.

### 3.5. Cell Culture and Viability Assay

The RAW 264.7 mouse macrophage cell line was obtained from the Cell Bank of Shanghai Institute of Biochemistry and Cell Biology (Chinese Academy of Sciences, Shanghai, China), and was cultured in Dulbecco’s modified Eagle medium (DMEM, Gibco Invitrogen Corp., Carlsbad, CA, USA). The cells were supplemented with 3.0 mM glutamine, antibiotics (100 U/mL penicillin and 100 U/mL streptomycin), and 10% heat-inactivated fetal bovine serum at 37 °C under a humidified atmosphere of 5% CO_2_. The cell viability of the cultured cells was assessed by MTT assay. Briefly, RAW 264.7 mouse macrophage cells were incubated with 200 μL MTT solution (0.5 mg/mL in medium) for 4 h at 37 °C, and then the supernatants were removed and residues were dissolved in 100 μL DMSO. The absorbance was detected at 490 nm using a microplate reader (Molecular Devices, Silicon Valley, CA, USA) and analyzed using a Soft Max Pro 5 software (Molecular Devices, Silicon Valley, CA, USA).

### 3.6. Measurement of NO Production

The NO concentration was measured by the Griess reaction. Briefly, RAW 264.7 macrophage cells were cultured onto 96-well plates at a density of 5 × 10^4^ cell/well and then incubated with or without 1.0 μg/mL LPS (Escherichia coli 055:B5, Sigma, St. Louis, MO, USA) in the absence or presence of compounds for 24 h. After that, 50 μL of culture supernatant was allowed to react with equal volume of Griess reagent (1% sulfanilamide, 0.1% *N*-1-naphthylethylenediamine dihydrochloride in 5% phosphoric acid) for 10 min at rt in the dark. Then, the optical density was measured at 540 nm using a microplate reader (Molecular Devices, Silicon Valley, CA, USA). Sodium nitrite was used as a standard to calculate the nitrite concentration. Inhibition (%) = (1 − (A_LPS + sample_ − A_untreated_)/(A_LPS_ − A_untreated_)) × 100. The experiments were performed in triplicates, and the data were expressed as the mean ± standard deviation (SD) values. Quercetin was used as a positive control.

## 4. Conclusions

The chemical investigation of the South China Sea coral *S*. *elegans* led to the isolation of two novel biscembranoids, sarelengans A and B (**1** and **2**), five new cembranoids, sarelengans C–G (**3**–**7**), along with two known cembranoids (**8** and **9**). Compounds **1** and **2** represent the first example of biscembranoids featuring a *trans*-fused A/B-ring conjunction between the two cembranoid units. Their unique structures may shed light on an unusual biosynthetic pathway involving a cembranoid-∆^8^ rather than the normal cembranoid-∆^1^ unit in the *endo*-Diels-Alder cycloaddition. All compounds were tested for their inhibitory activity against nitric oxide (NO) production induced by lipopolysaccharide (LPS) in RAW 264.7 macrophages, and compounds **2** and **3** showed moderate inhibitory activities with IC_50_ values being at 18.2 and 32.5 μM, respectively. The current study expanded the members of the biscembranoid family.

## Figures and Tables

**Table 1:** ^1^H (400 MHz) and ^13^C (100 MHz) NMR (nuclear magnetic resonance) data for **1** and **2** in methanol-*d*_4_ (δ in ppm).

No.	1		2	
	δ_C_, Type	δ_H_, Multi. (*J* in Hz)	δ_C_, Type	δ_H_, Multi. (*J* in Hz)
1	36.7, CH	3.15, m	36.5, CH	3.14, m
2	43.0, CH_2_	a 3.06, dd (17.6, 4.6)	43.3, CH_2_	a 3.07, dd (14.0, 4.4)
		b 2.86, m		b 2.89, m
3	203.4, C		202.5, C	
4	127.4, CH	5.96, d (1.2)	126.7, CH	6.03, d (1.1)
5	154.1, C		156.0, C	
6	34.1, CH_2_	a 2.80, m	33.2, CH_2_	a 2.54, m
		b 2.07, m		b 2.36, m
7	36.7, CH_2_	1.05, m	35.6, CH_2_	1.06, m
8	33.2, CH	2.30, m	33.0, CH	2.33, m
9	55.9, C		56.4, C	
10	215.5, C		214.7, C	
11	41.1, CH_2_	a 2.99, dd (18.0, 10.6)	41.8, CH_2_	2.95, m
		b 2.61, dd (18.0, 3.3)		
12	54.8, CH	2.51, m	55.8, CH	2.35, m
13	215.4, C		215.5, C	
14	44.1, CH_2_	a 3.44, dd (18.1, 3.7)	42.0, CH_2_	a 3.18, dd (17.9, 3.8)
		b 2.87, m		b 2.74, dd (17.9, 6.2)
15	31.2, CH	1.70, m	31.4, CH	1.72, m
16	21.0, CH_3_	0.92, d (6.7)	21.1, CH_3_	0.96, d (6.7)
17	21.2, CH_3_	0.92, d (6.7)	21.2, CH_3_	0.90, d (6.7)
18	16.8, CH_3_	1.07, s	16.5, CH_3_	1.13, s
19	26.1, CH_3_	1.86, d (1.2)	27.4, CH_3_	1.90, d (1.1)
20	176.3, C		176.6, C	
21	41.6, CH	3.68, d (10.6)	42.0, CH	3.40, d (11.2)
22	128.2, CH	4.88, d (10.6)	126.4, CH	4.89, d (11.2)
23	136.9, C		133.1, C	
24	38.3, CH_2_	2.00, m	35.5, CH_2_	a 2.22, m
				b 1.99, m
25	32.2, CH_2_	a 1.79, m	26.6, CH_2_	1.41, m
		b 1.43, m		
26	76.2, CH	3.47, m	64.0, CH	2.90, m
27	84.4, C		61.2, C	
28	37.1, CH_2_	a 2.08, m	39.4, CH_2_	a 1.99, m
		b 1.69, m		b 1.01, m
29	29.4, CH_2_	1.76, m	24.3, CH_2_	a 2.22, m
				b 1.98, m
30	79.3, CH	3.89, dd (10.2, 4.2)	130.6, CH	5.14, dd (9.1, 4.8)
31	151.6, C		135.6, C	
32	72.0, CH	4.07, d (9.7)	78.7, CH	3.80, d (10.0)
33	76.4, CH	4.39, d (9.7)	71.8, CH	4.66, d (10.0)
34	130.2, C		130.7, C	
35	132.7, C		132.7, C	
36	38.9, CH_2_	a 2.45, m	38.7, CH_2_	a 2.55, m
		b 1.62, m		b 1.65, m
37	20.2, CH_3_	1.54, s	19.6, CH_3_	1.71, s
38	18.1, CH_3_	1.70, s	18.9, CH_3_	1.59, s
39	20.3, CH_3_	1.08, s	16.3, CH_3_	1.30, s
40	112.8, CH_2_	a 5.47, s	11.4, CH_3_	1.61, s
		b 5.21, s		
41	52.3, CH_3_	3.63, s	52.3, CH_3_	3.64, s

**Table 2:** ^1^H-NMR (400 MHz) data of **3**–**7** (δ in ppm, *J* in Hz).

No.	3 ^a^	4 ^a^	5 ^a^	6 ^a^	7 ^b^
2	6.29, d (12.1)	5.45, d (15.5)	5.69, d (15.9)	5.50, d (16.2)	5.57, d (15.7)
3	7.42, dd (12.1, 1.1)	5.53, d (15.5)	5.64, d (15.9)	5.56, d (16.2)	5.66, d (15.7)
5		a 1.93, m	a 1.75, m	1.74, m	a 1.89, m
		b 1.79, m	b 1.59, ddd (13.4, 10.7, 7.0)		b 1.68, m
6	5.64, s	2.65, m	a 2.79, ddd (20.4, 10.9, 7.0)	a 2.65, m	1.54, m
			b 2.35, ddd (20.4, 10.7, 2.4)	b 2.35, m	
7					4.37, m
9	a 2.09, m	a 2.72, m	a 1.92, m	a 2.06, m	a 2.26, m
	b 2.04, m	b 2.18, m	b 1.81, m	b 1.74, m	b 1.99, m
10	a 2.74, m	2.41, m	a 3.37, m	a 2.04, m	a 2.58, m
	b 2.64, m		b 2.18, m	b 1.72, m	b 2.49, m
11	5.99, t (6.4)	6.09, m	5.73, m	4.87, m	6.34, t (4.3)
12				2.69, m	
13	a 2.73, m	2.37, m	2.46, m	1.82, m	a 2.99, m
	b 2.13, m				b 2.13, m
14	2.48, t (6.2)	1.73, m	a 2.05, ddd (13.9, 6.6, 2.1)	a 2.04, m	a 2.00, m
			b 1.77, m	b 1.82, m	b 1.73, m
15	2.52, m	1.72, m	1.85, m	1.89, m	1.80, m
16	1.12, d (6.8)	0.92, d (6.8)	0.96, d (6.7)	0.98, d (6.8)	0.84, d (6.8)
17	1.12, d (6.8)	0.92, d (6.8)	0.98, d (6.7)	1.00, d (6.8)	0.89, d (6.8)
18	1.96, d (1.1)	1.29, s	1.26, s	1.27, s	1.31, s
19	1.43, s	1.43, s	1.23, s	1.31, s	1.32 s
21	3.66, s				

^a^ in methanol-*d*_4_; ^b^ in CDCl_3_.

**Table 3:** ^13^C-NMR (100 MHz) data of **3**–**7** (δ in ppm).

No.	3 ^a^	4 ^a^	5 ^a^	6 ^a^	7 ^b^
1	164.1, C	78.2, C	88.1, C	89.6, C	76.1, C
2	119.3, CH	131.8, CH	128.7, CH	129.8, CH	134.6, CH
3	136.6, CH	134.9, CH	139.8, CH	139.9, CH	132.4, CH
4	121.7, C	73.6, C	73.5, C	74.0, C	75.2, CH
5	187.7, C	34.3, CH_2_	38.1, CH_2_	36.9, CH_2_	34.3, CH_2_
6	100.4, CH	34.3, CH_2_	36.1, CH_2_	34.5, CH_2_	25.7, CH_2_
7	209.6, C	212.0, C	217.8, C	215.1, C	66.1, CH
8	91.3, C	88.1, C	79.8, C	90.0, C	83.6, C
9	40.5, CH_2_	37.4, CH_2_	42.1, CH_2_	36.7, CH_2_	34.8, CH_2_
10	25.8, CH_2_	24.8, CH_2_	25.9, CH_2_	27.5, CH_2_	27.4, CH_2_
11	152.1, CH	133.8, CH	150.9, CH	80.8, CH	141.0, CH
12	130.1, C	137.5, C	125.2, C	44.1, CH	134.1, C
13	36.5, CH_2_	26.5, CH_2_	25.1, CH_2_	17.9, CH_2_	31.0, CH_2_
14	31.6, CH_2_	33.0, CH_2_	29.1, CH_2_	28.2, CH_2_	39.7, CH_2_
15	38.6, CH	38.8, CH	38.3, CH	38.0, CH	36.3, CH
16	21.8, CH_3_	18.1, CH_3_	17.5, CH_3_	17.6, CH_3_	17.2, CH_3_
17	21.4, CH_3_	17.0, CH_3_	16.9, CH_3_	17.0, CH_3_	16.0, CH_3_
18	12.2, CH_3_	31.4, CH_3_	26.9, CH_3_	24.4, CH_3_	33.5, CH_3_
19	20.9, CH_3_	28.0, CH_3_	28.3, CH_3_	24.0, CH_3_	21.6, CH_3_
20	169.2, C	172.1, C	168.9, C	175.3, C	168.4, C
21	51.5, CH_3_				

^a^ in methanol-*d*_4_; ^b^ in CDCl_3_.

## Supplementary Materials

The following are available online at www.mdpi.com/1660-3397/15/4/85/s1, HRESIMS, 1D and 2D NMR spectra of **1**–**7**, ^1^H and ^13^C-NMR spectra of known compounds **8** and **9**.
