# Supplementary material for: Biscembranoids and Cembranoids from the Soft Coral Sarcophyton elegans
Source: Mar Drugs. 2017 Mar 23;15(4):85. doi: 10.3390/md15040085 (PMC5408232; doi:10.3390/md15040085)

Biscembranoids and cembranoids from the South China Sea Soft Coral *Sarcophyton elegans*

Wei Li, Yi-Hong Zou, Man-Xi Ge, Lan-Lan Lou, Yun-Shao Xu, Abrar Ahmed, Yun-Yun Chen, Jun-Sheng Zhang, Gui-Hua Tang, Sheng Yin*

School of Pharmaceutical Sciences, Sun Yat-sen University, Guangzhou, Guangdong 510006, People's Republic of China

E-mail: yinsh2@mail.sysu.edu.cn; Fax: +86-20-39943090; Tel: +86-20-39943090.

Contents:

S1 ^{1}H NMR spectrum of **1** in Methanol-d_4

S2 ^{13}C NMR spectrum of **1** in Methanol-d_4

S3 HSQC spectrum of **1** in Methanol-d_4

S4 HMBC spectrum of **1** in Methanol-d_4

S5 ^{1}H–^{1}H COSY spectrum of **1** in Methanol-d_4

S6 NOESY spectrum of **1** in Methanol-d_4

S7 HRESIMS spectrum of **1**

S8 ^{1}H NMR spectrum of **2** in Methanol-d_4

S9 ^{13}C NMR spectrum of **2** in Methanol-d_4

S10 HSQC spectrum of **2** in Methanol-d_4

S11 HMBC spectrum of **2** in Methanol-d_4

S12 ^{1}H–^{1}H COSY spectrum of **2** in Methanol-d_4

S13 NOESY spectrum of **2** in Methanol-d_4

S14 HRESIMS spectrum of **2**

S15 ^{1}H NMR spectrum of **3** in Methanol-d_4

S16 ^{13}C NMR spectrum of **3** in Methanol-d_4

S17 HSQC spectrum of **3** in Methanol-d_4

S18 HMBC spectrum of **3** in Methanol-d_4

S19 ^{1}H–^{1}H COSY spectrum of **3** in Methanol-d_4

S20 NOESY spectrum of **3** in Methanol-d_4

S21 HRESIMS spectrum of **3**

S22 ^{1}H NMR spectrum of **4** in Methanol-d_4

S23 ^{13}C NMR spectrum of **4** in Methanol-d_4

S24 HSQC spectrum of **4** in Methanol-d_4

S25 HMBC spectrum of **4** in Methanol-d_4

S26 ^{1}H–^{1}H COSY spectrum of **4** in Methanol-d_4

S27 NOESY spectrum of **4** in Methanol-d_4

S28 HRESIMS spectrum of **4**

S29 ^{1}H NMR spectrum of **5** in Methanol-d_4

S30 ^{13}C NMR spectrum of **5** in Methanol-d_4

S31 HSQC spectrum of **5** in Methanol-d_4

S32 HMBC spectrum of **5** in Methanol-d_4

S33 ^{1}H–^{1}H COSY spectrum of **5** in Methanol-d_4

S34 NOESY spectrum of **5** in Methanol-d_4

S35 HRESIMS spectrum of **5**

S36 ^{1}H NMR spectrum of **6** in Methanol-d_4

S37 ^{13}C NMR spectrum of **6** in Methanol-d_4

S38 HSQC spectrum of **6** in Methanol-d_4

S39 HMBC spectrum of **6** in Methanol-d_4

S40 ^{1}H–^{1}H COSY spectrum of **6** in Methanol-d_4

S41 NOESY spectrum of **6** in Methanol-d_4

S42 HRESIMS spectrum of **6**

S43 ^{1}H NMR spectrum of **7** in $CDCl_3$

S44 ^{13}C NMR spectrum of **7** in $CDCl_3$

S45 HSQC spectrum of **7** in $CDCl_3$

S46 HMBC spectrum of **7** in $CDCl_3$

S47 $^{1}H–^{1}H$ COSY spectrum of **7** in $CDCl_3$

S48 NOESY spectrum of **7** in $CDCl_3$

S49 HRESIMS spectrum of **7**

S50 ^{1}H NMR spectrum of **8** in Methanol-d_4

S51 ^{13}C NMR spectrum of **8** in Methanol-d_4

S52 ^{1}H NMR spectrum of **8** in $CDCl_3$

S53 ^{13}C NMR spectrum of **8** in $CDCl_3$

S54 ^{1}H NMR spectrum of **9** in Methanol-d_4

S55 ^{13}C NMR spectrum of **9** in Methanol-d_4

S56 Selected NOESY correlations of **2**.

S1 ^{1}H NMR spectrum of **1** in Methanol-d_4

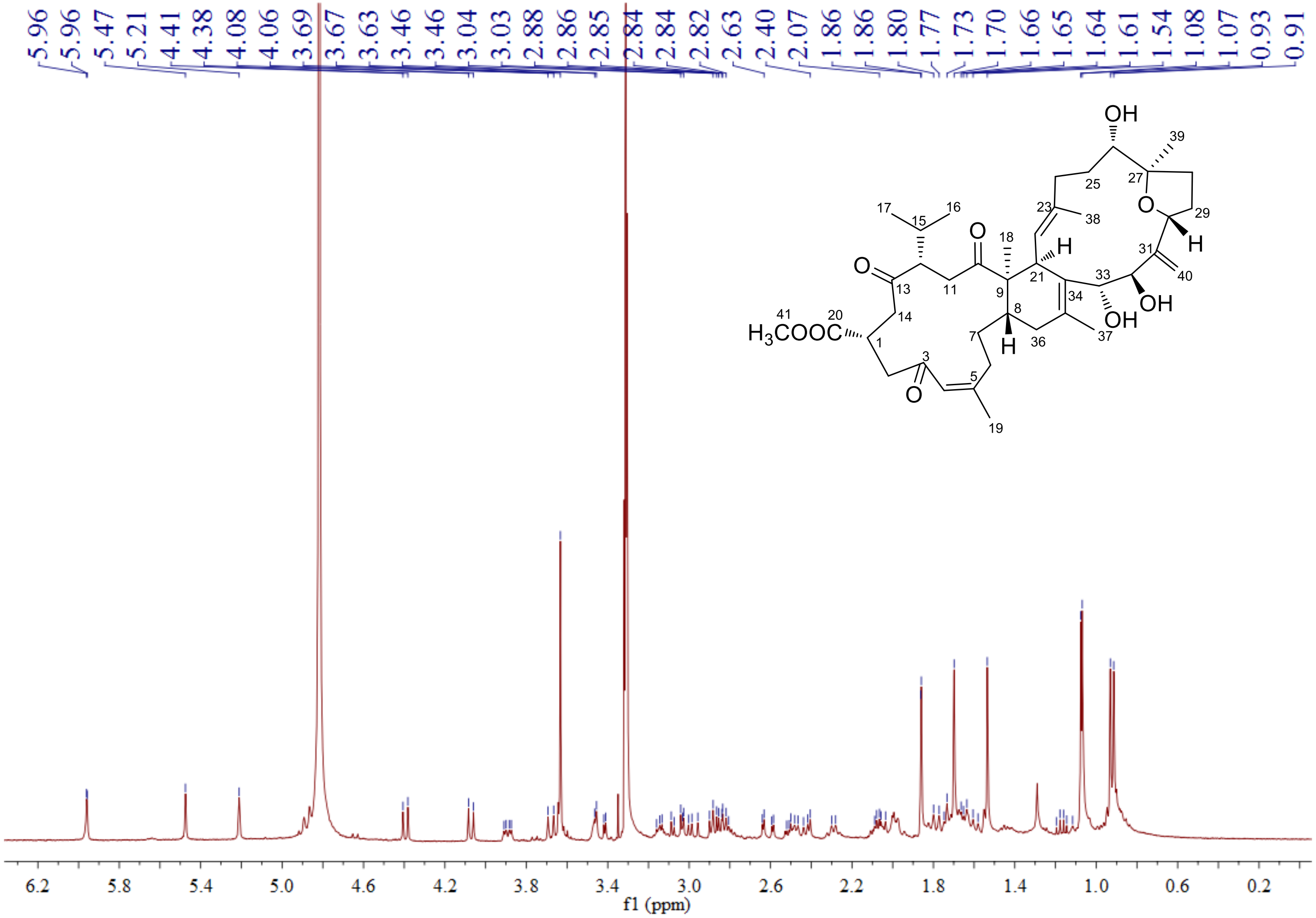

S2 ^{13}C NMR spectrum of **1** in Methanol-d_4

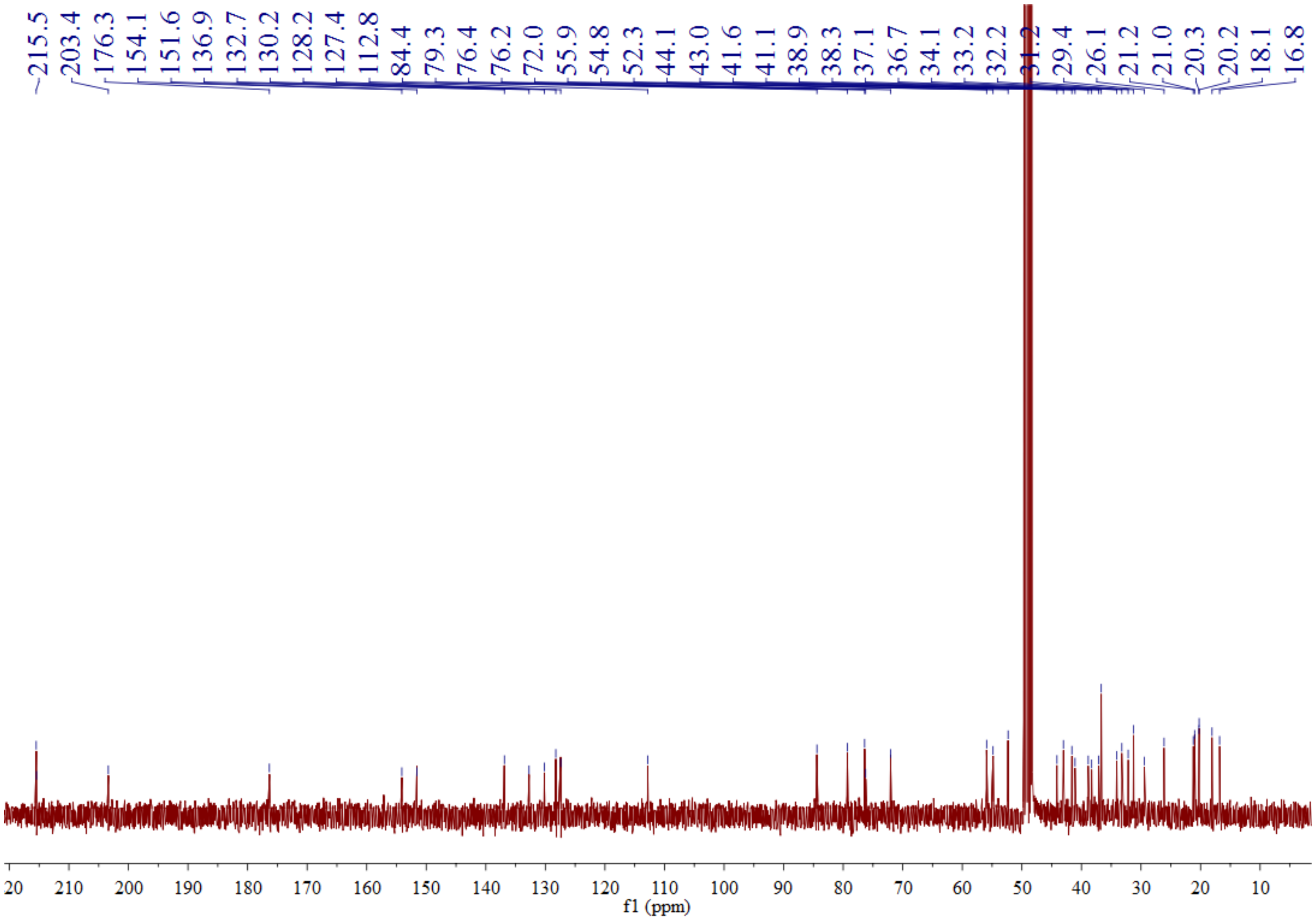

S3 HSQC spectrum of **1** in Methanol-d_4

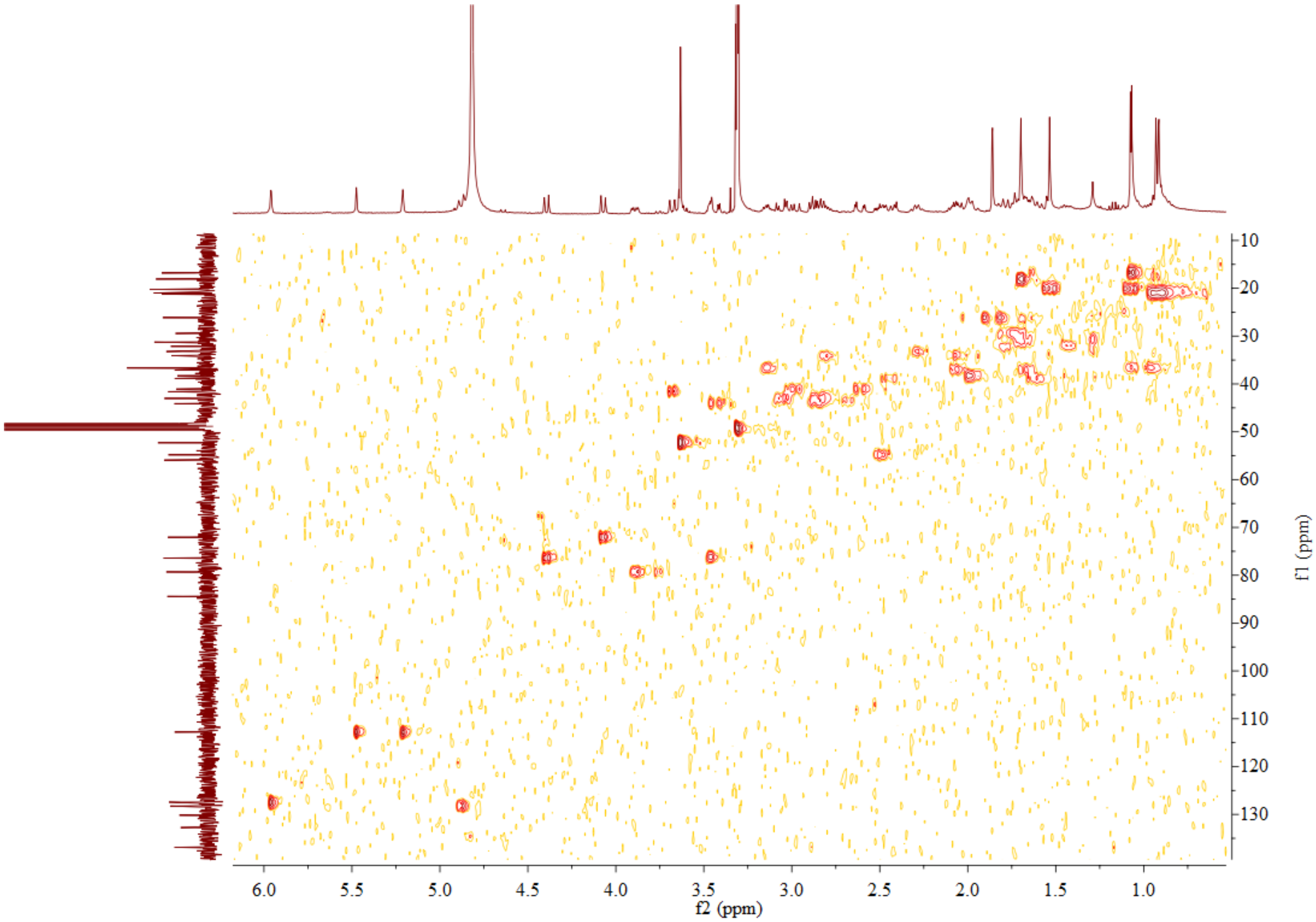

S4 HMBC spectrum of **1** in Methanol-d_4

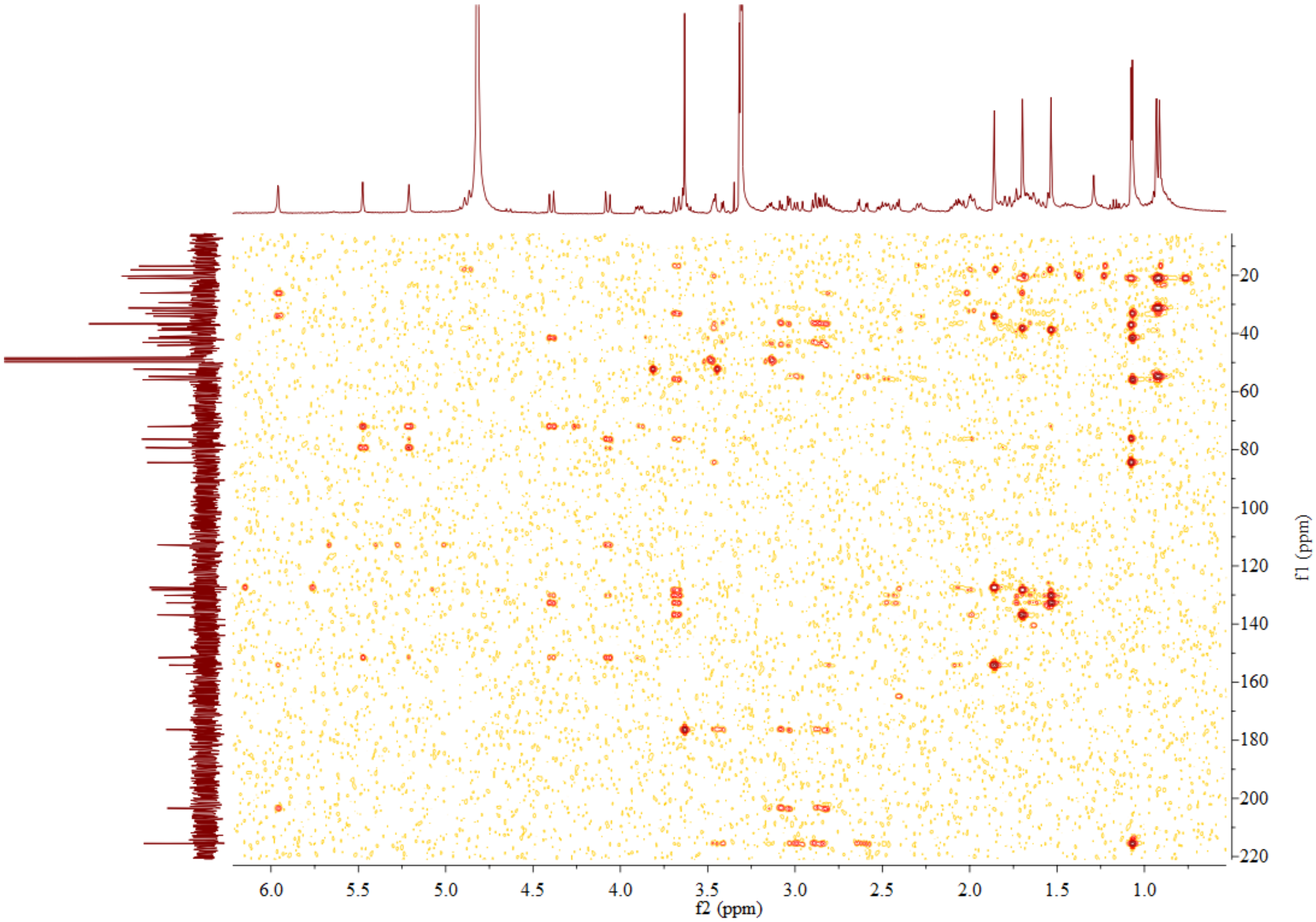

S5 ^1H–^1H COSY spectrum of **1** in Methanol-d_4

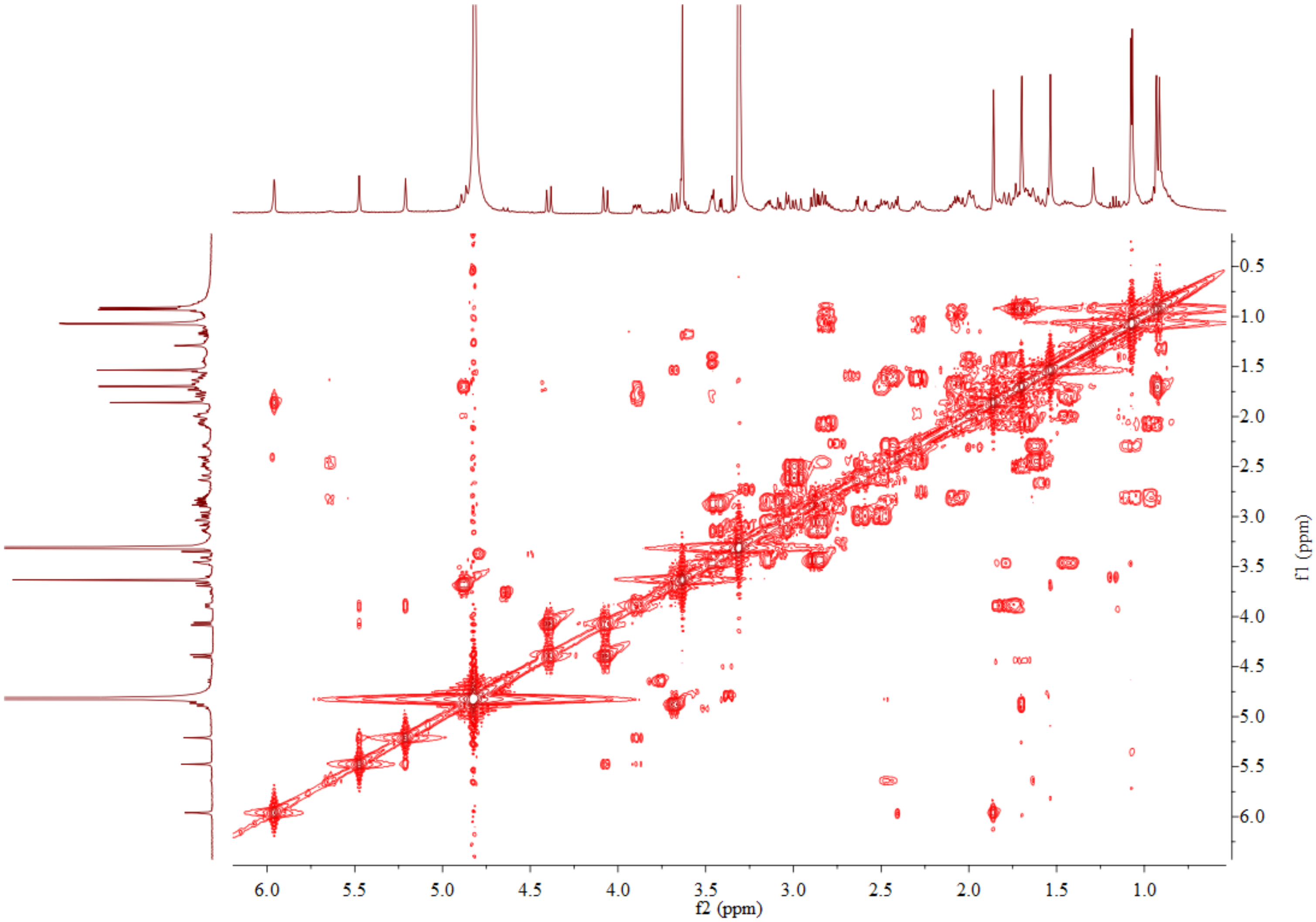

S6 NOESY spectrum of **1** in Methanol-d_4

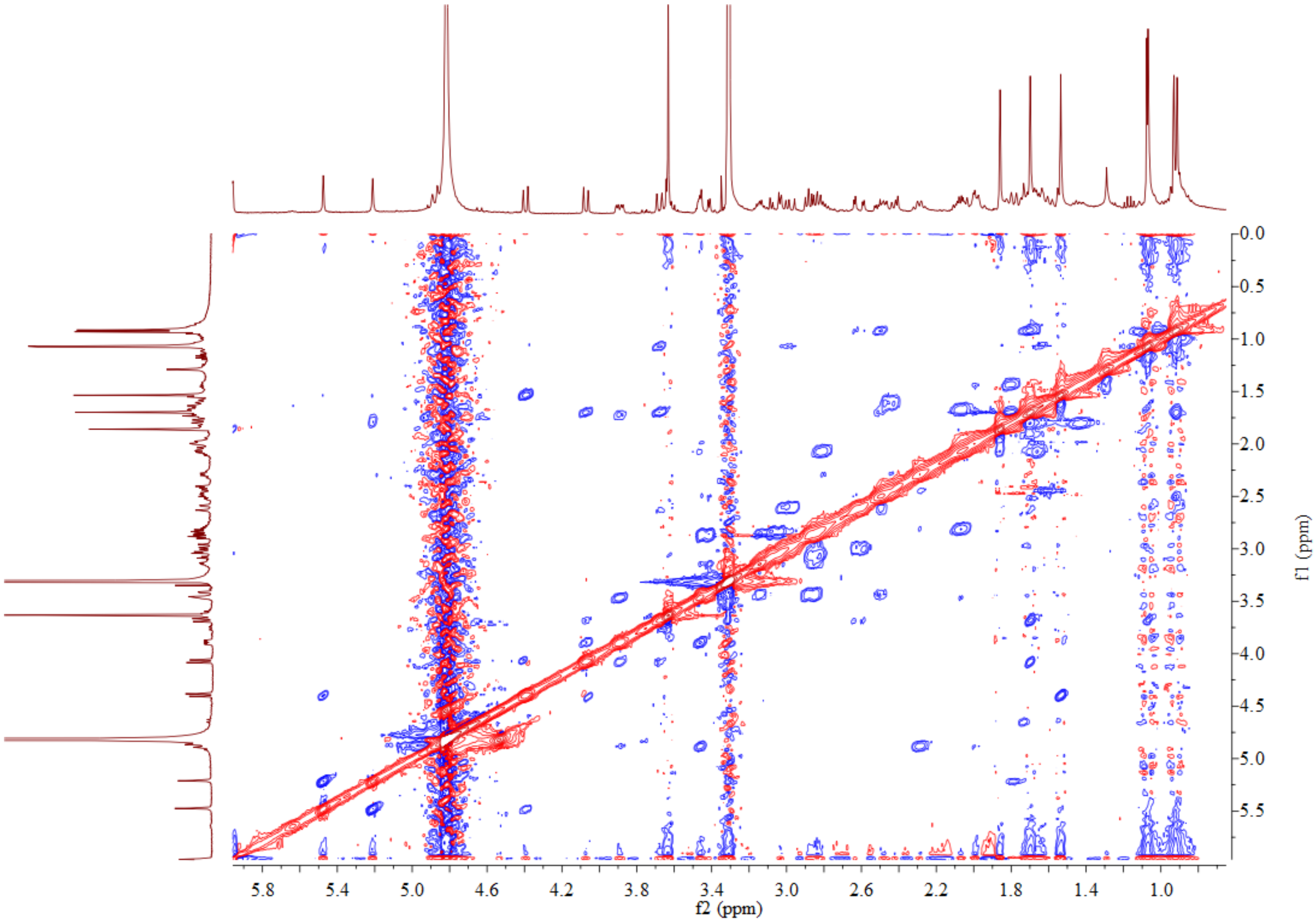

S7 HRESIMS spectrum of **1**

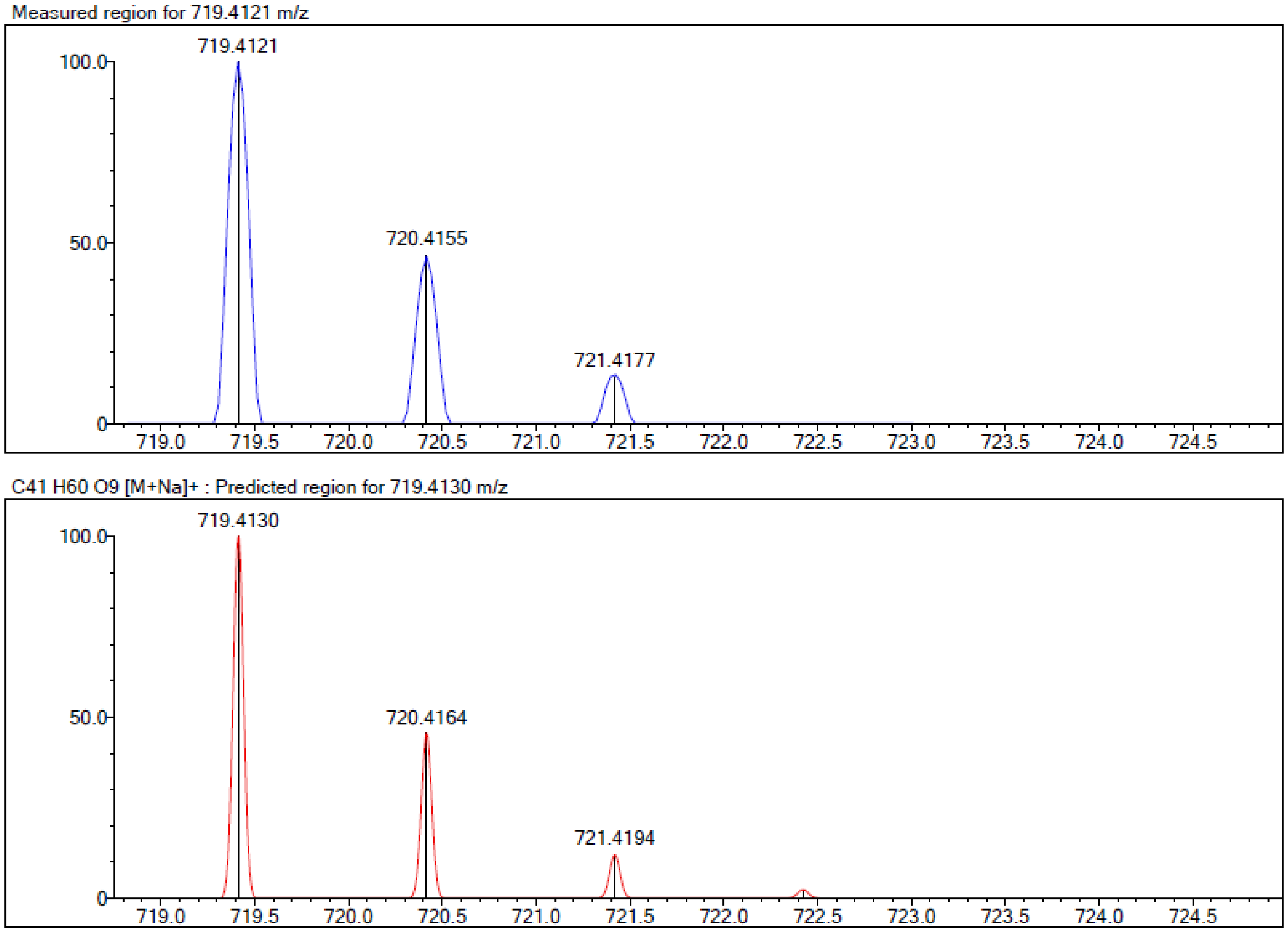

Rank	Score	Formula (M)	Ion	Meas. m/z	Pred. m/z	Df. (mDa)	Df. (ppm)	Iso	DBE
1	99.38	C41 H60 O9	[M+Na]+	719.4121	719.4130	-0.9	-1.25	100.00	12.0

S8 ^{1}H NMR spectrum of **2** in Methanol-d_4

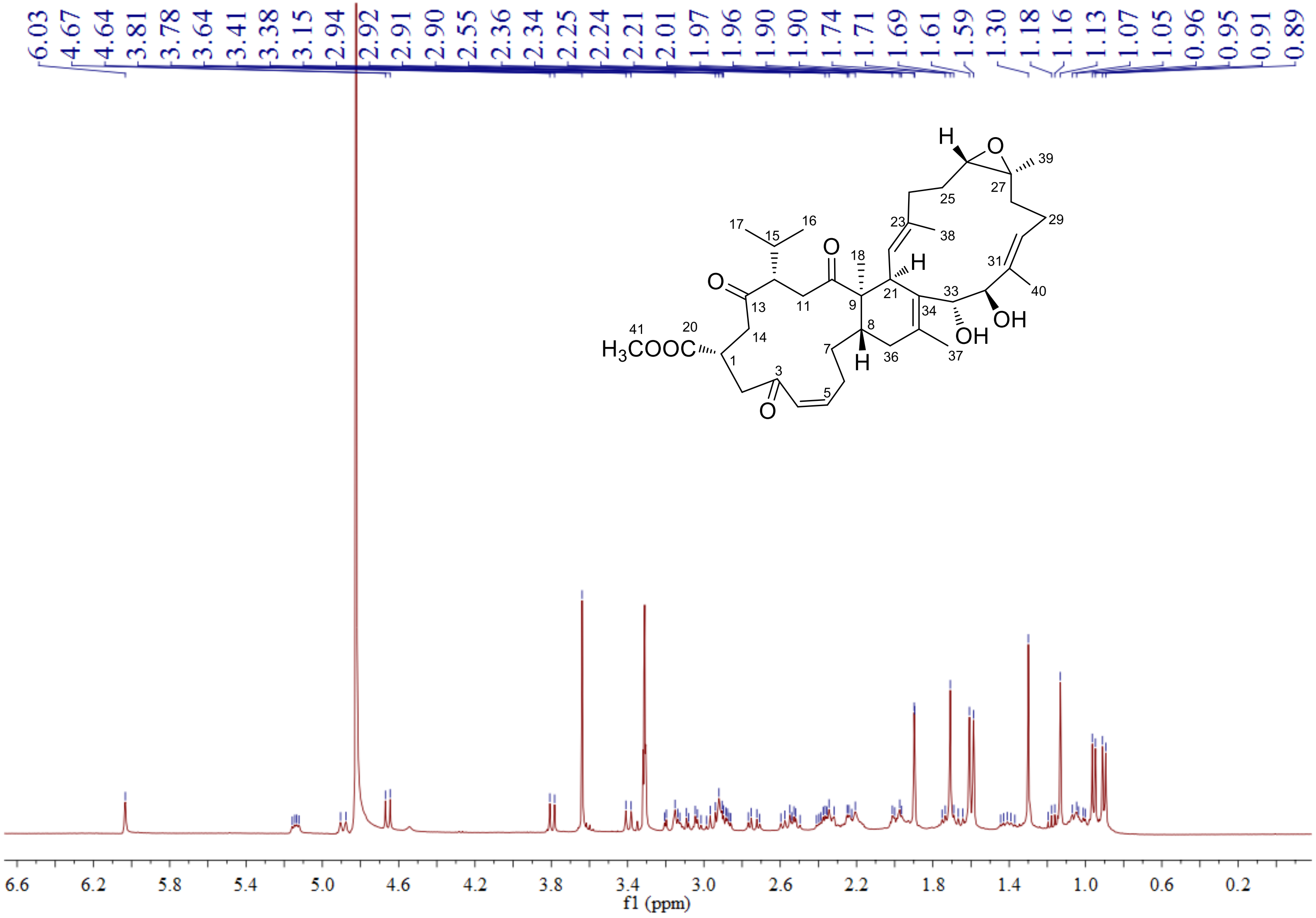

S9 ^{13}C NMR spectrum of **2** in Methanol-d_4

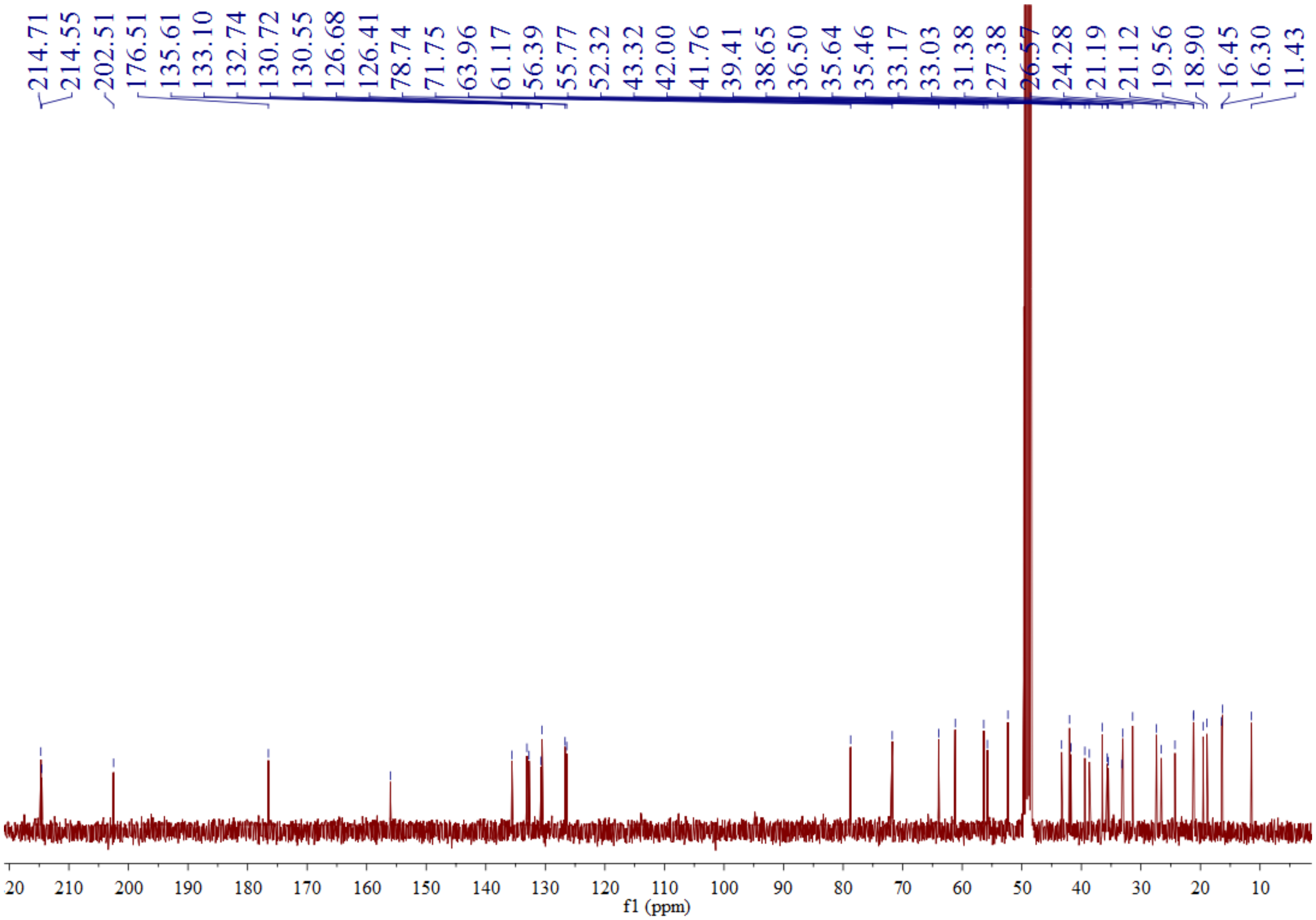

S10 HSQC spectrum of **2** in Methanol-d_4

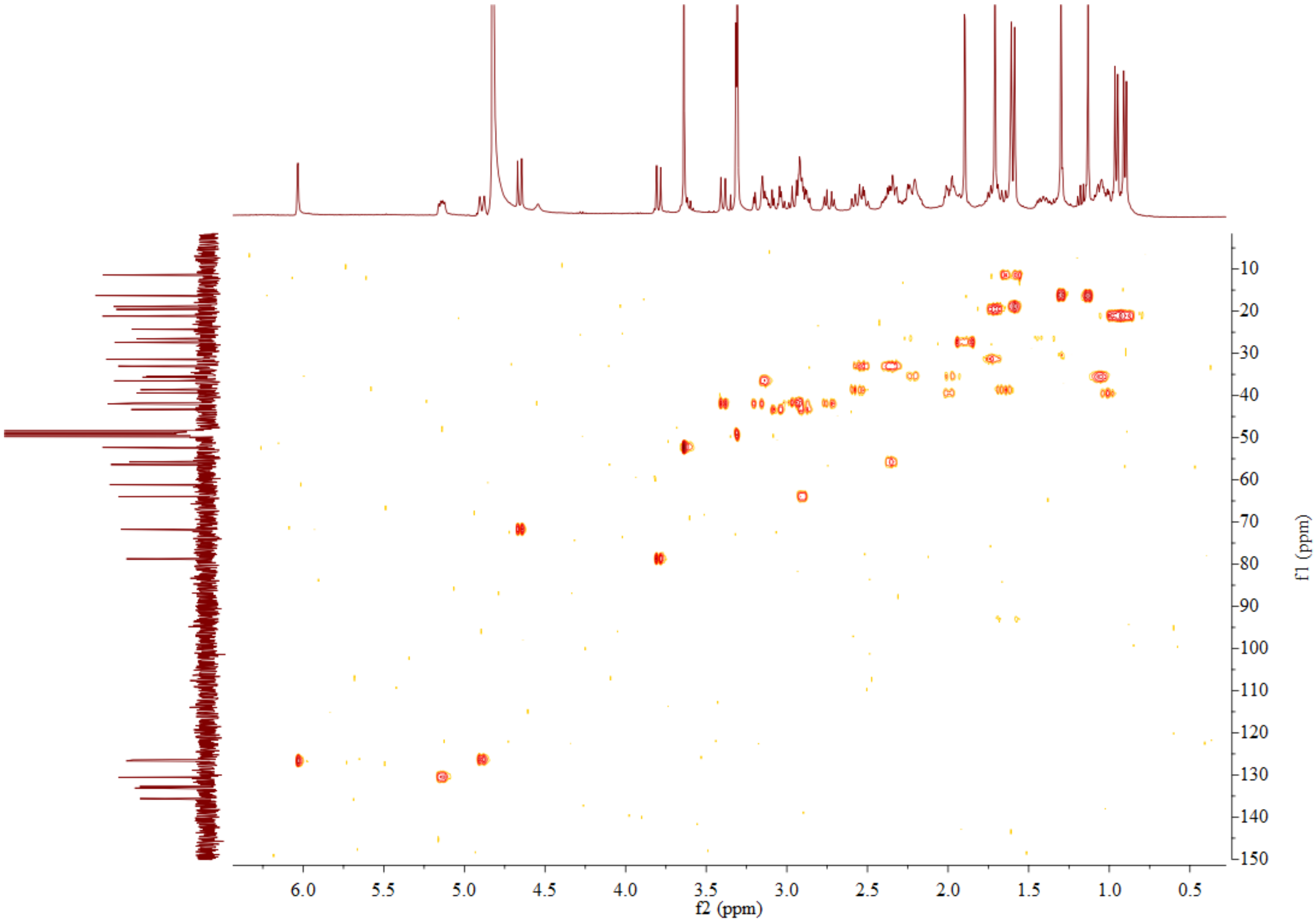

S11 HMBC spectrum of **2** in Methanol-d_4

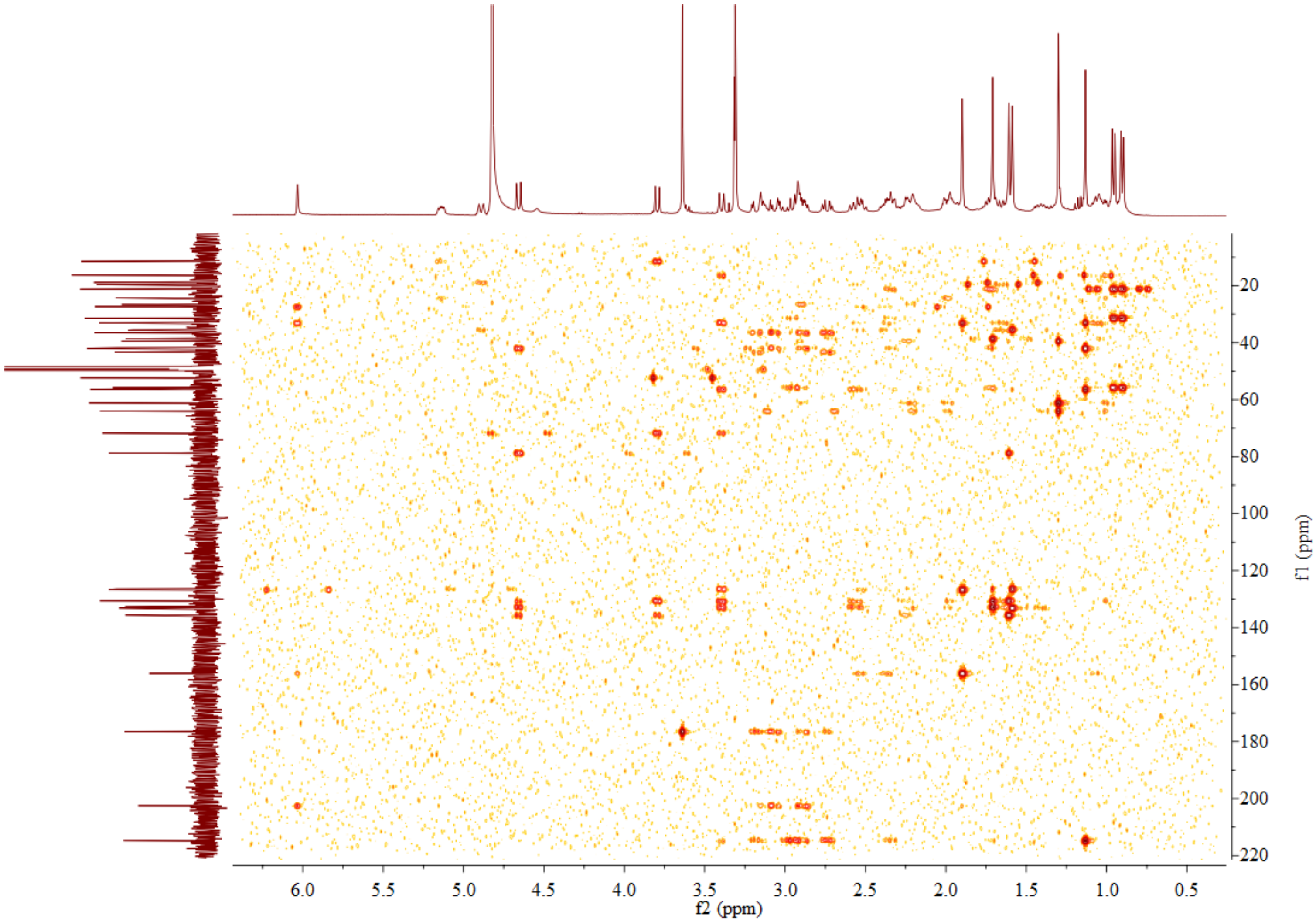

S12 ^{1}H–^{1}H COSY spectrum of **2** in Methanol-d_4

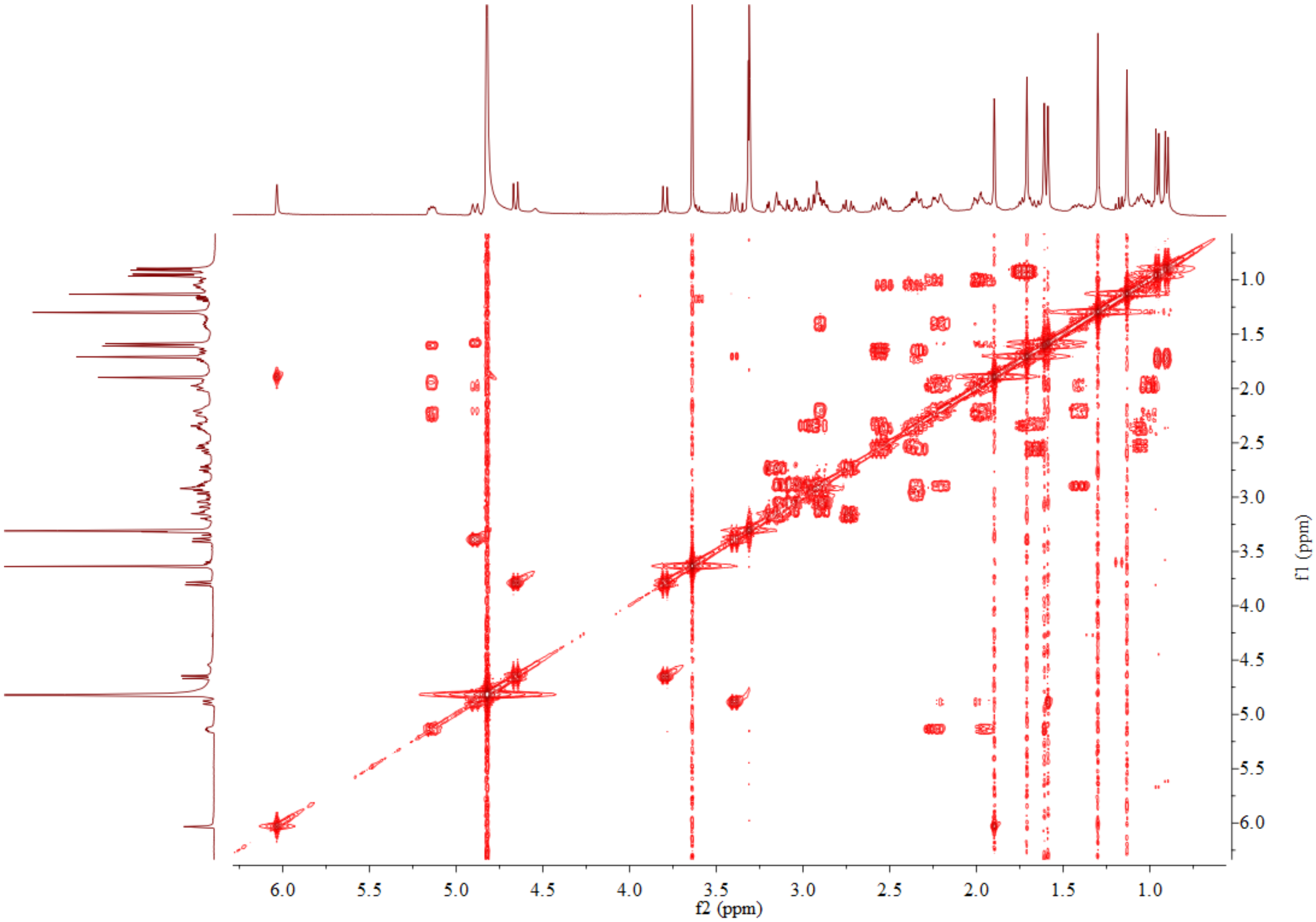

S13 NOESY spectrum of **2** in Methanol-d_4

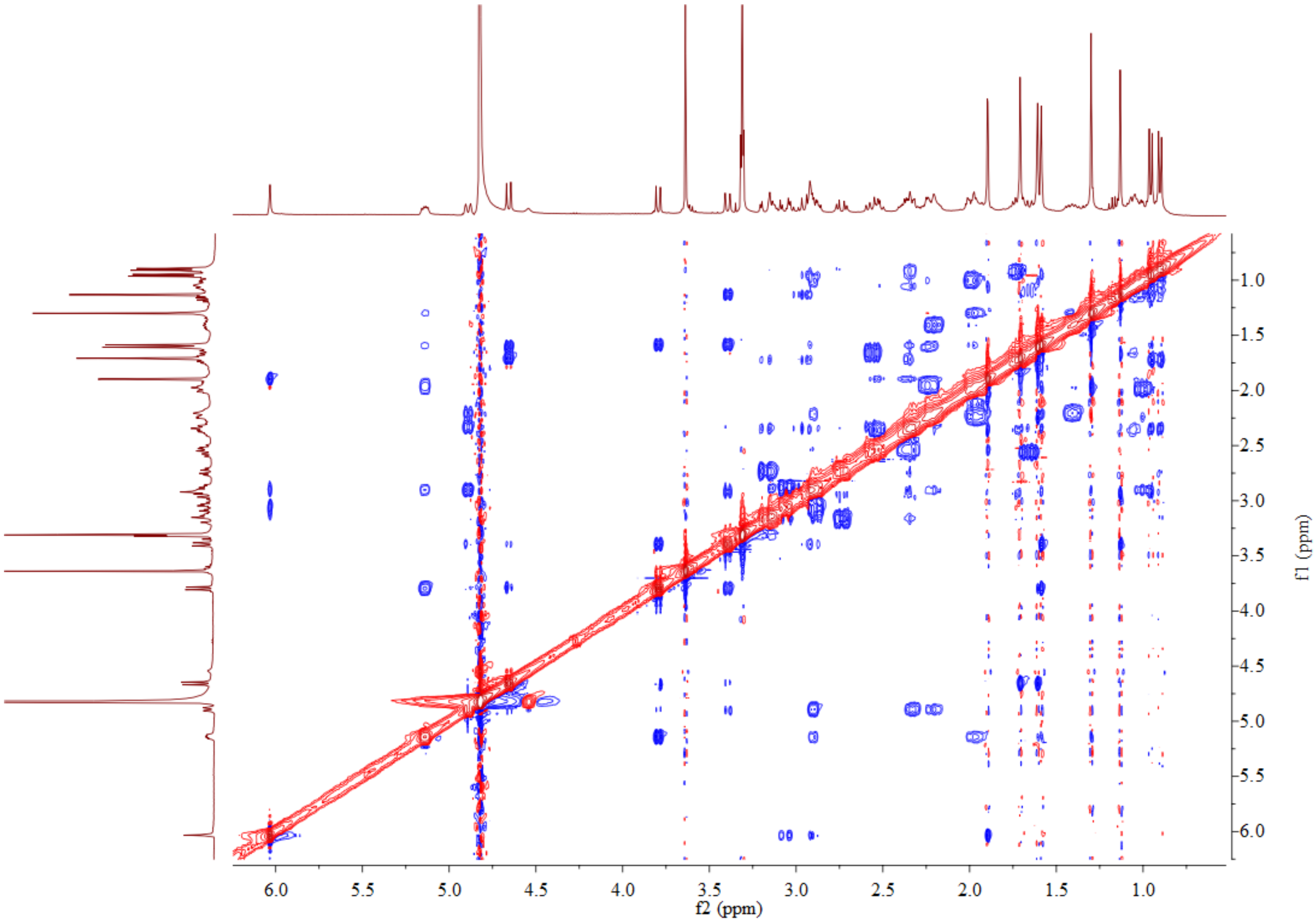

S14 HRESIMS spectrum of **2**

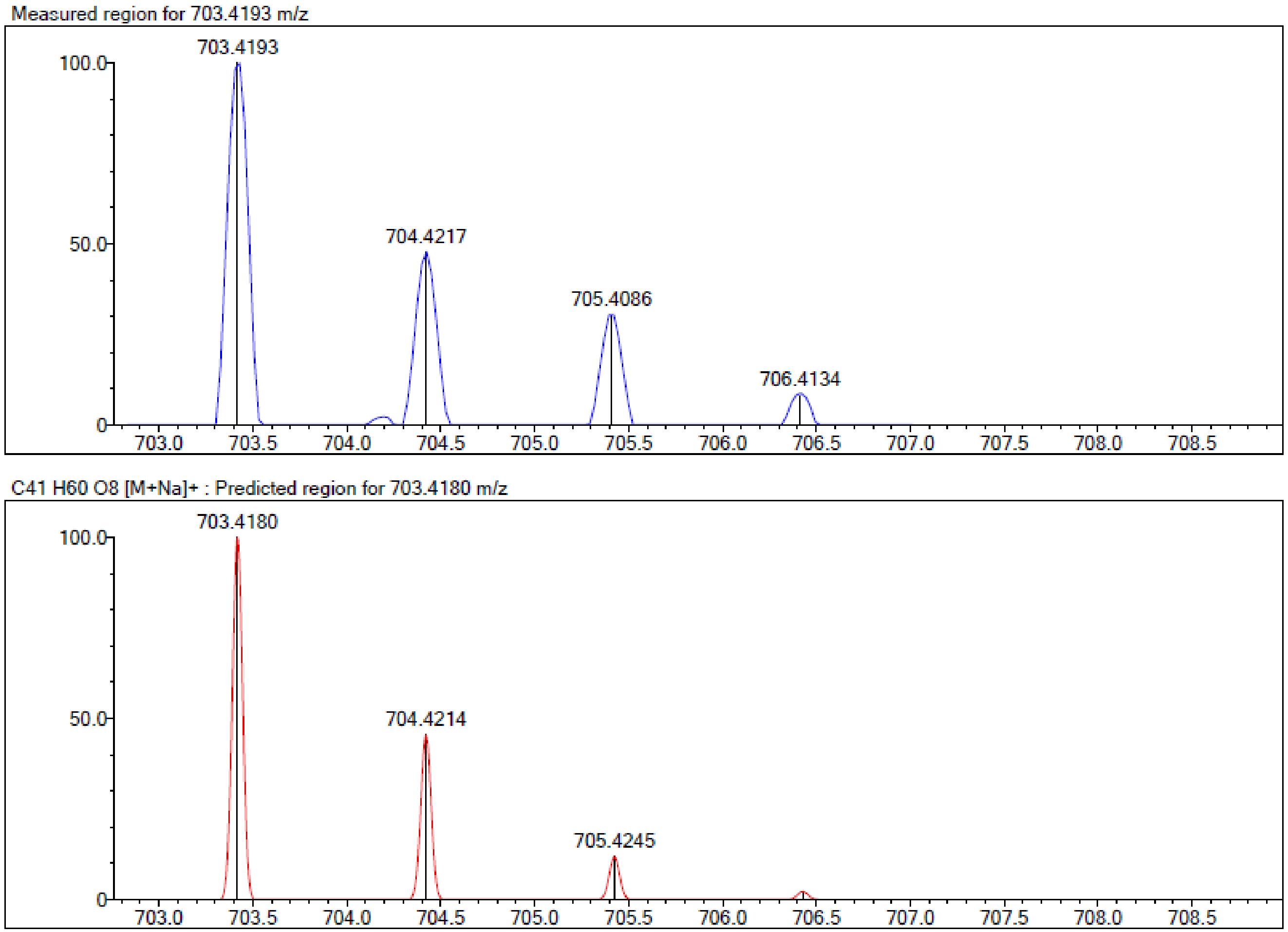

Rank	Score	Formula (M)	Ion	Meas. m/z	Pred. m/z	Df. (mDa)	Df. (ppm)	Iso	DBE
1	45.26	C41 H60 O8	[M+Na]+	703.4193	703.4180	1.3	1.85	46.24	12.0

S15 ^{1}H NMR spectrum of **3** in Methanol-d_4

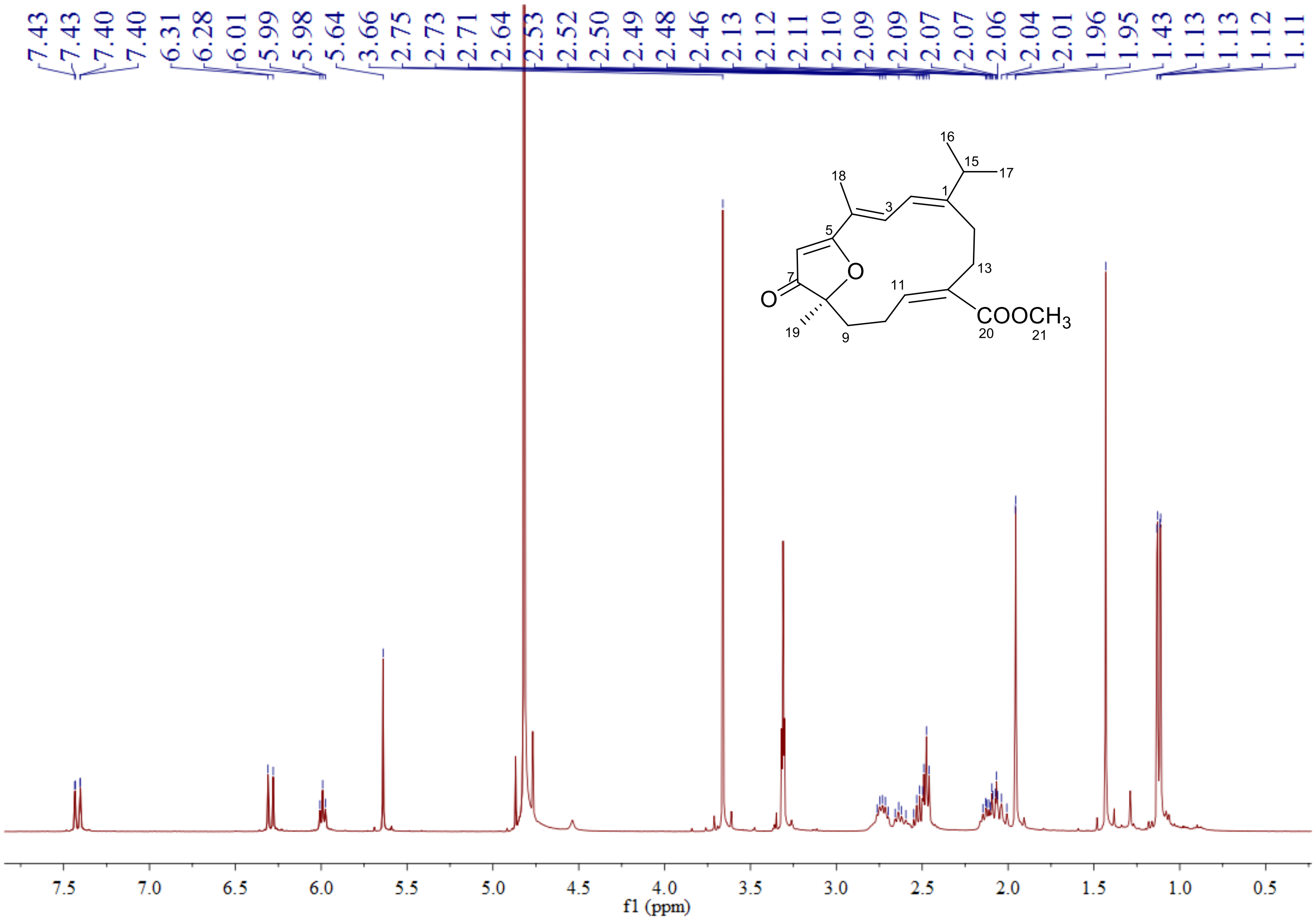

S16 ^{13}C NMR spectrum of **3** in Methanol-d_4

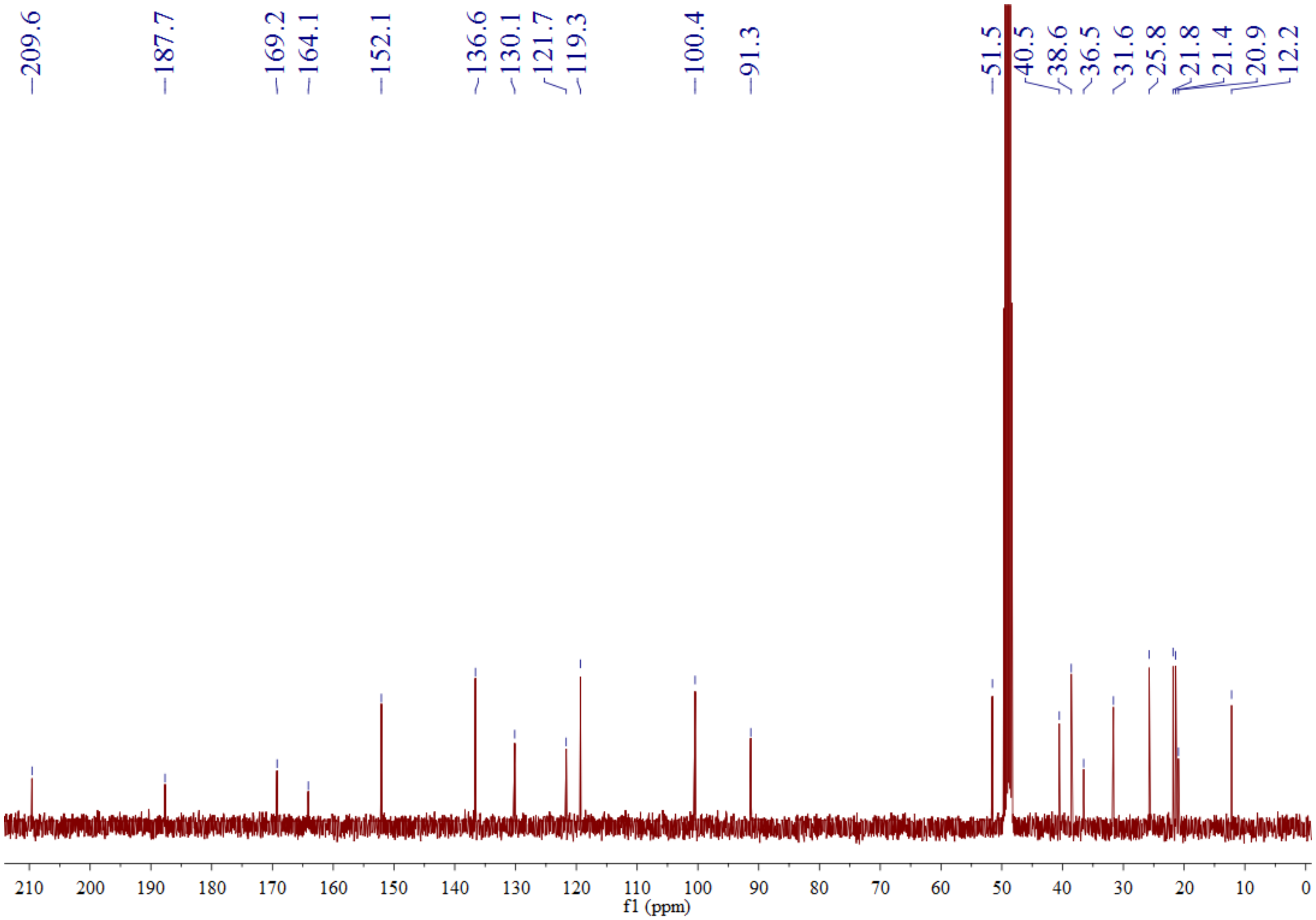

S17 HSQC spectrum of **3** in Methanol-d_4

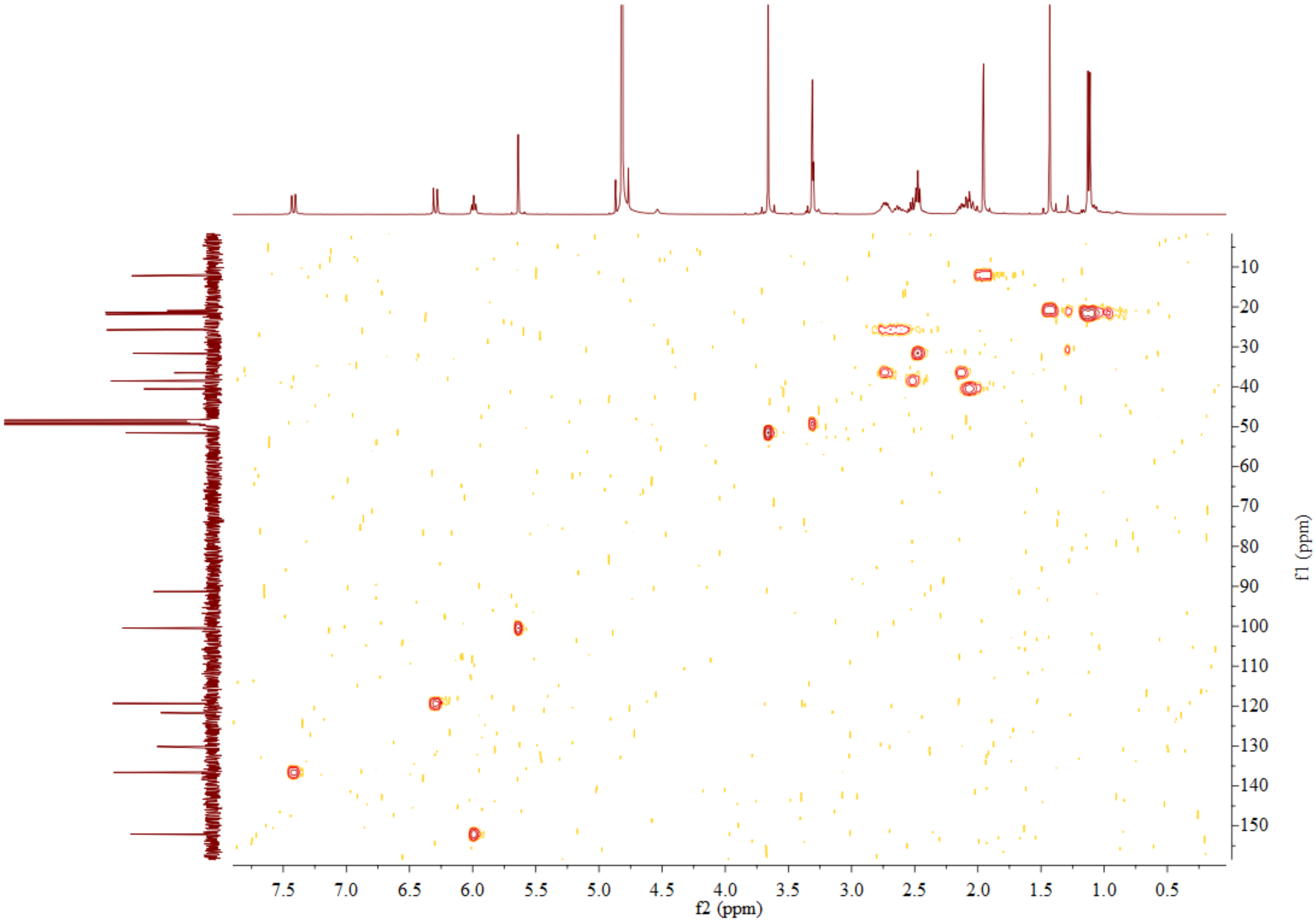

S18 HMBC spectrum of **3** in Methanol-d_4

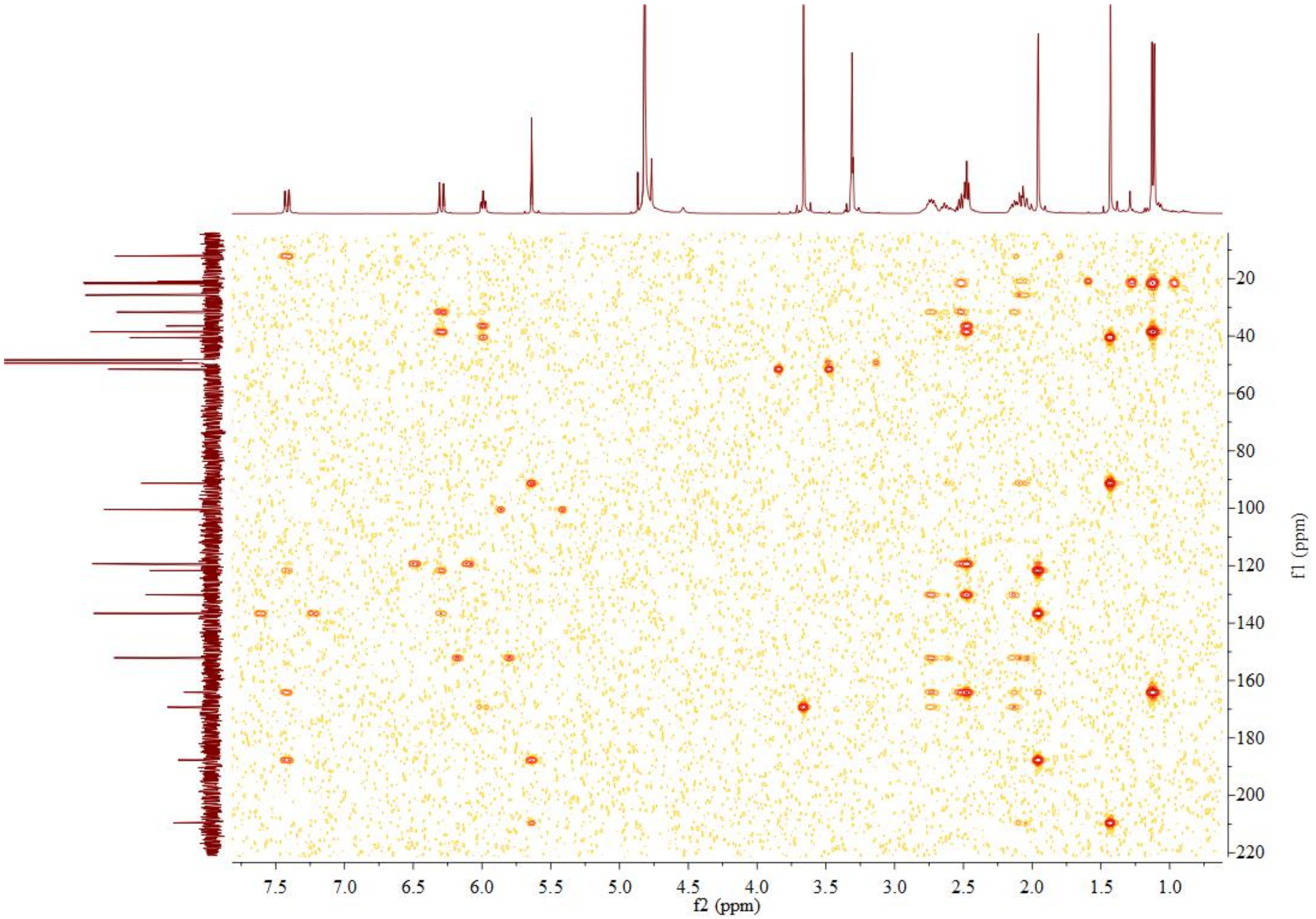

S19 $^{1}H–^{1}H$ COSY spectrum of **3** in Methanol-d_4

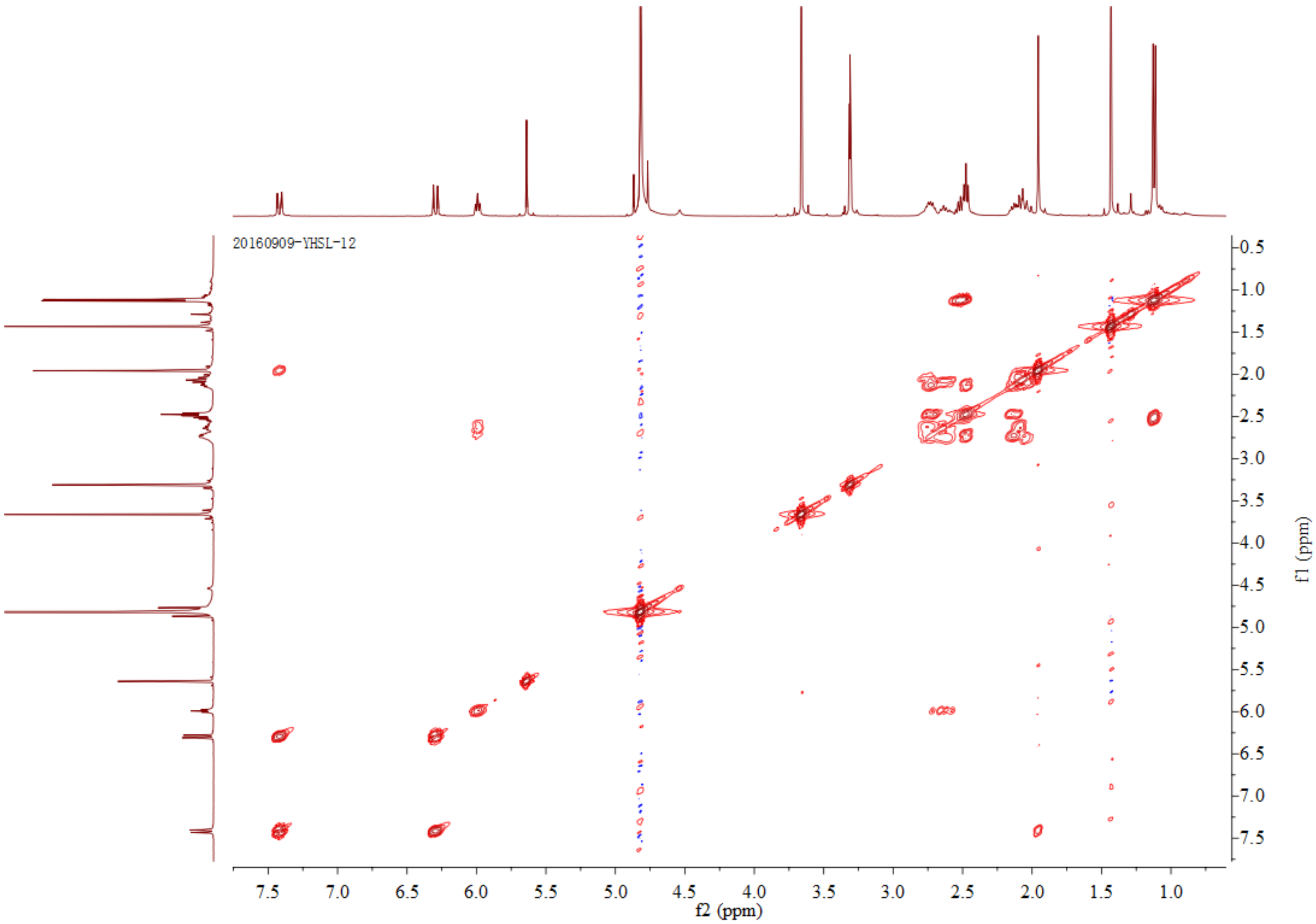

S20 NOESY spectrum of **3** in Methanol-d_4

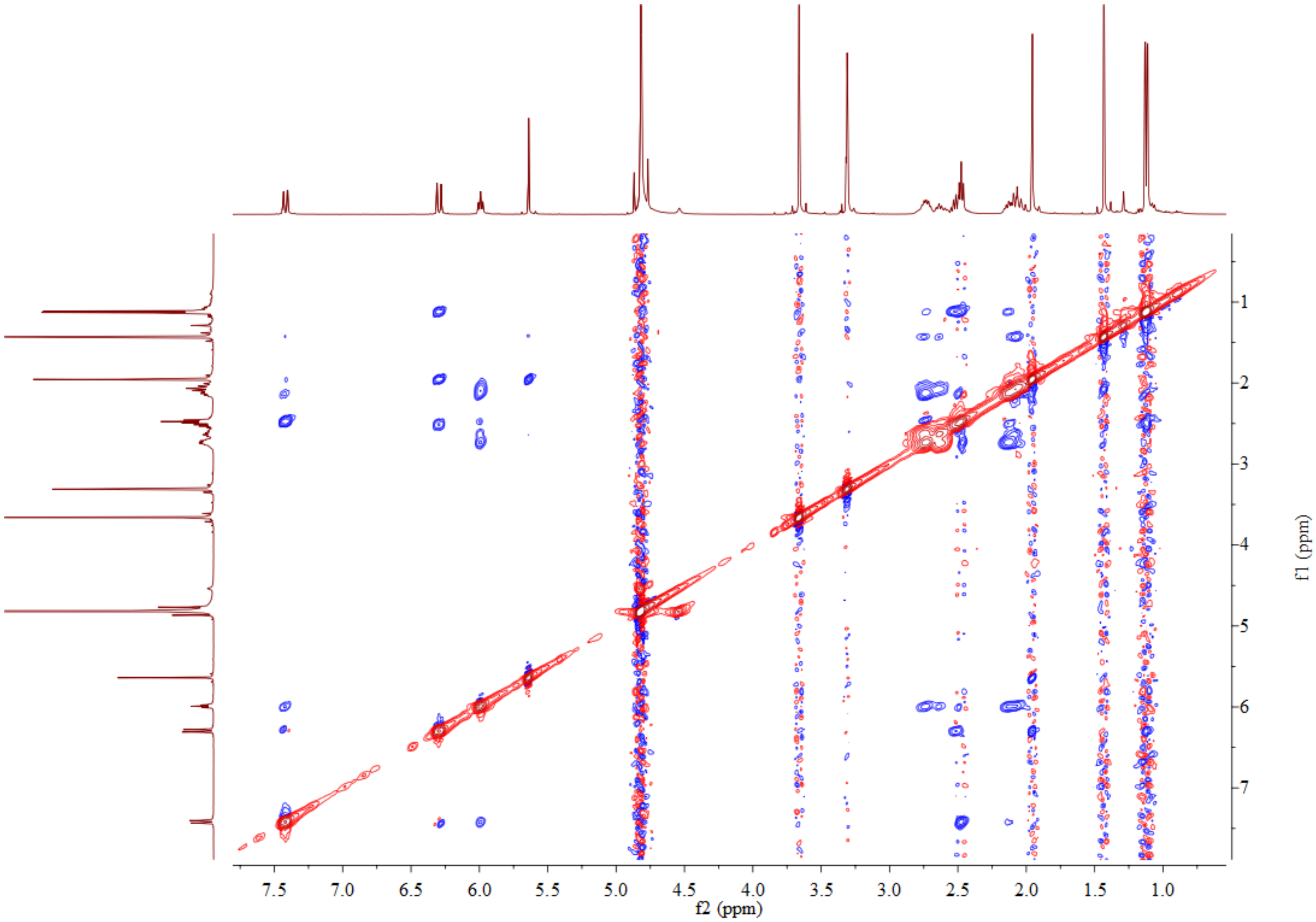

S21 HRESIMS spectrum of **3**

Measured region for 367.1871 m/z

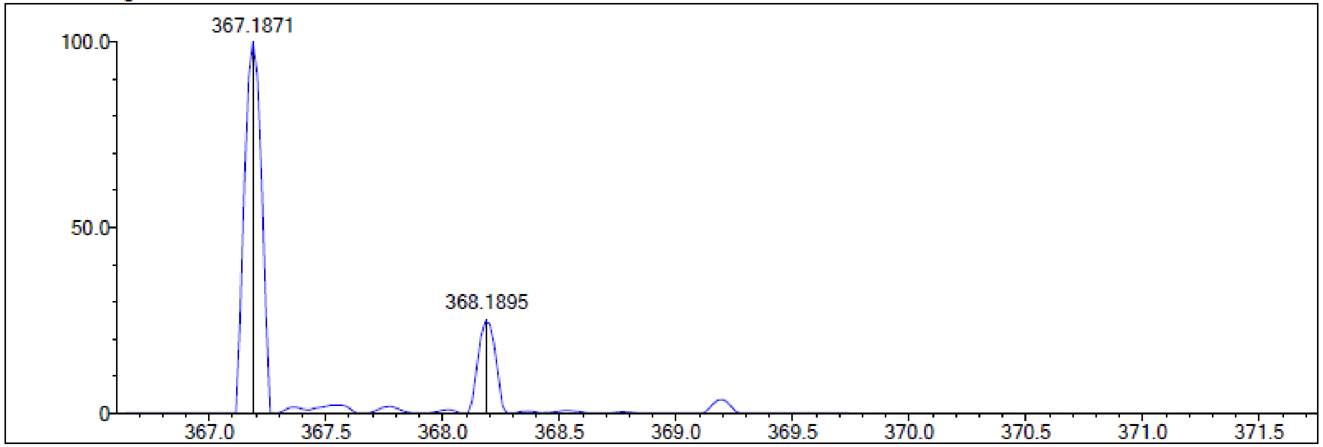

C21 H28 O4 [M+Na]+ : Predicted region for 367.1880 m/z

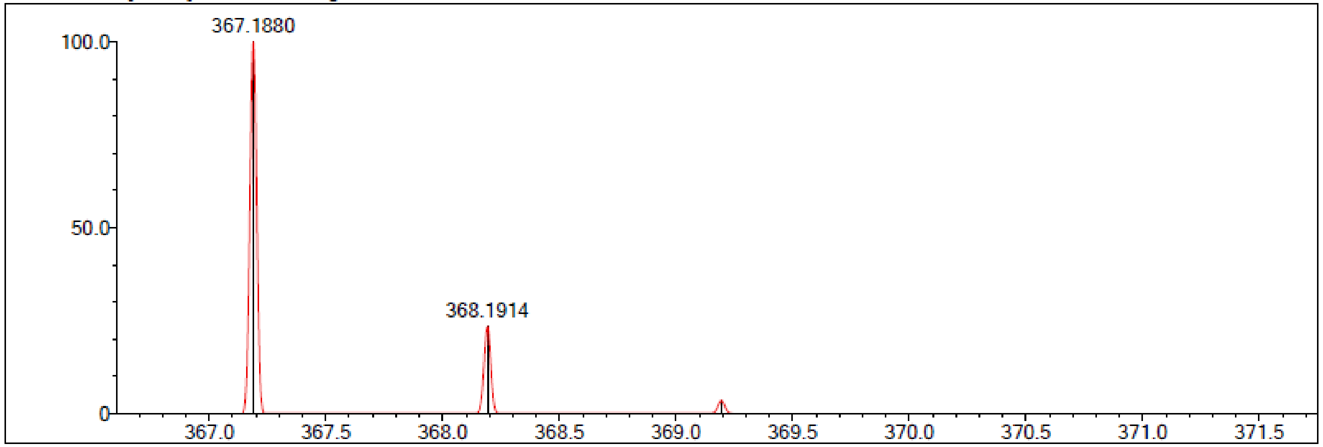

Rank	Score	Formula (M)	Ion	Meas. m/z	Pred. m/z	Df. (mDa)	Df. (ppm)	Iso	DBE
1	81.94	C21 H28 O4	[M+Na]+	367.1871	367.1880	-0.9	-2.45	85.02	8.0

S22 ^{1}H NMR spectrum of **4** in Methanol-d_4

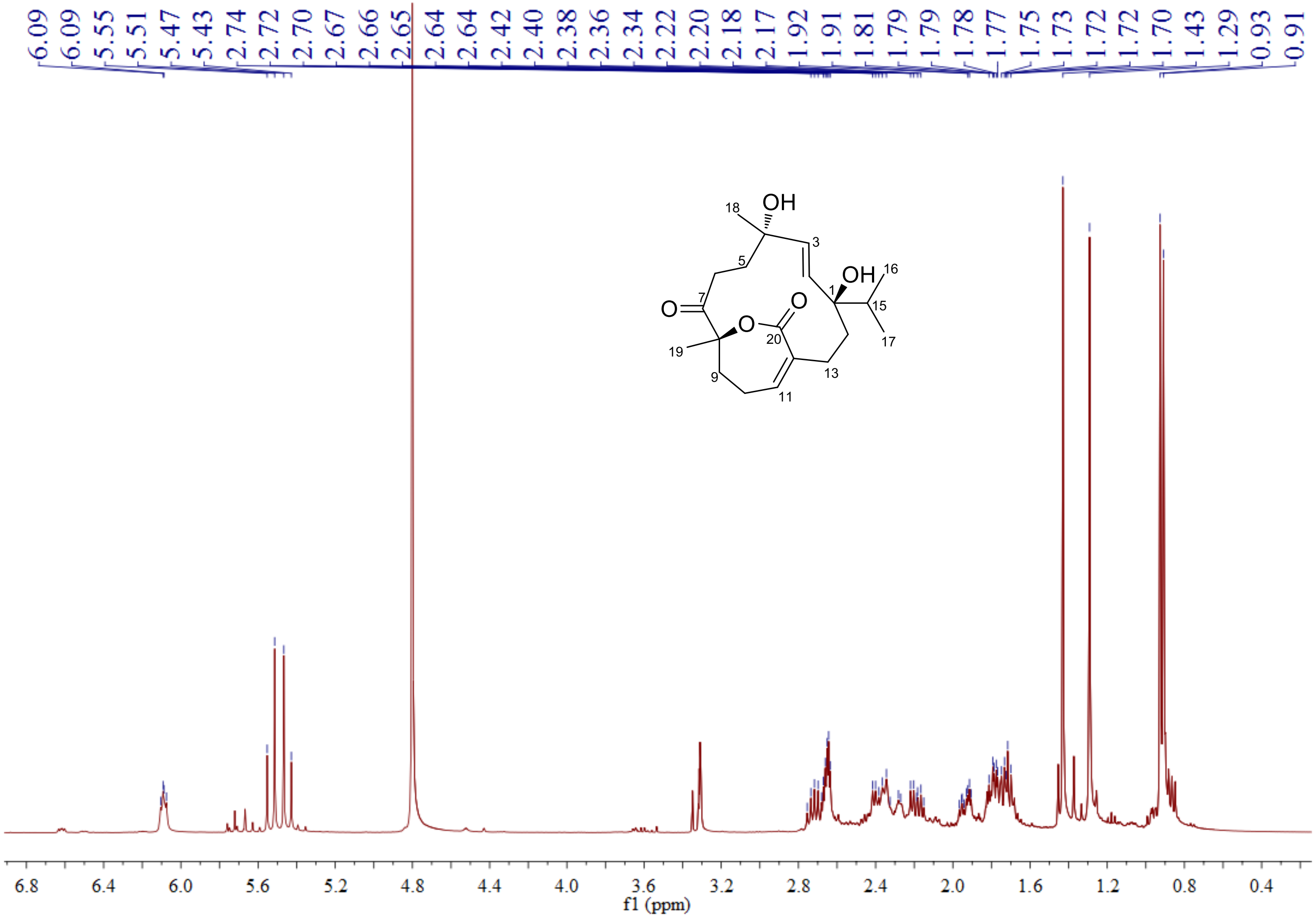

S23 ^{13}C NMR spectrum of **4** in Methanol-d_4

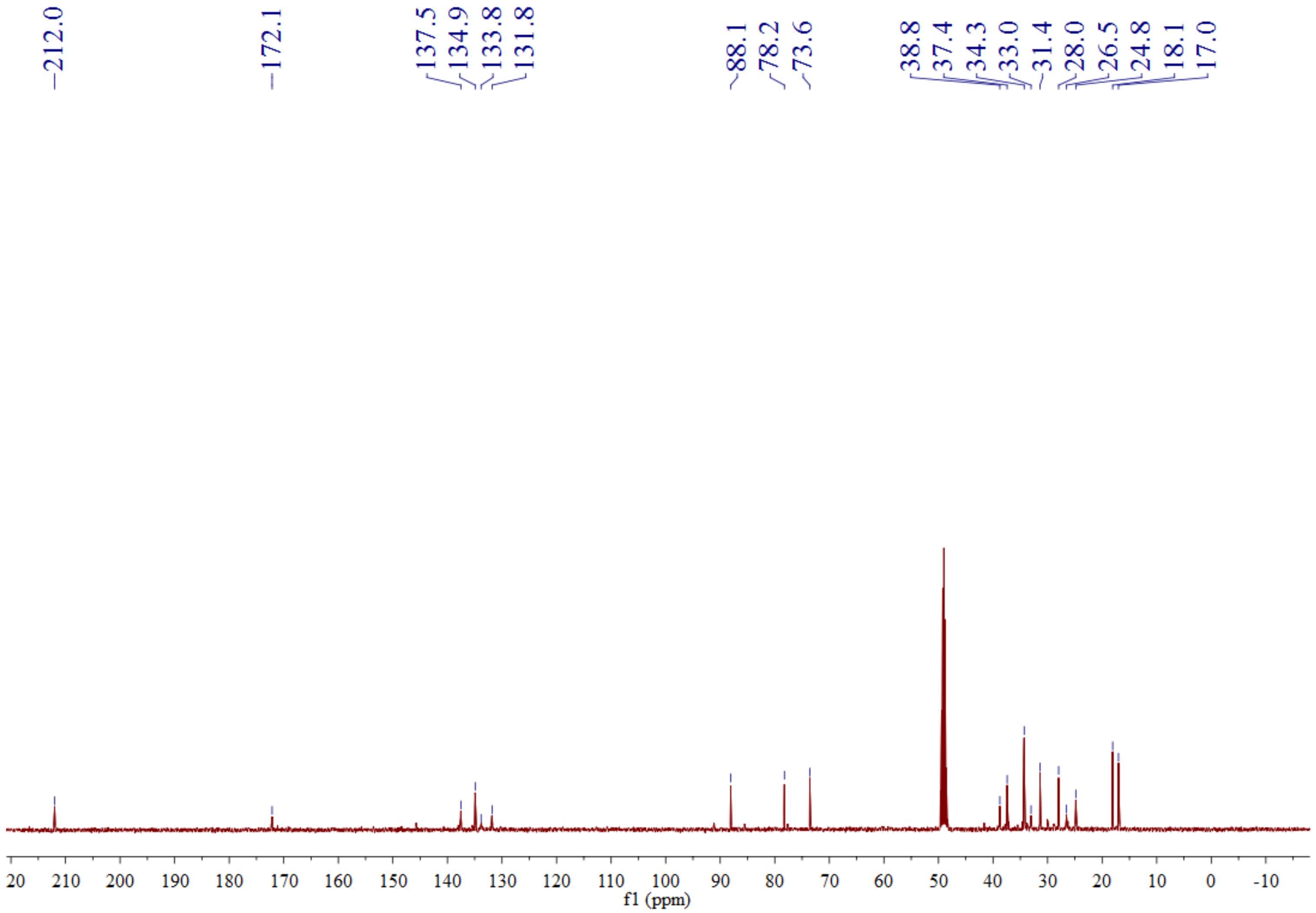

S24 HSQC spectrum of **4** in Methanol-d_4

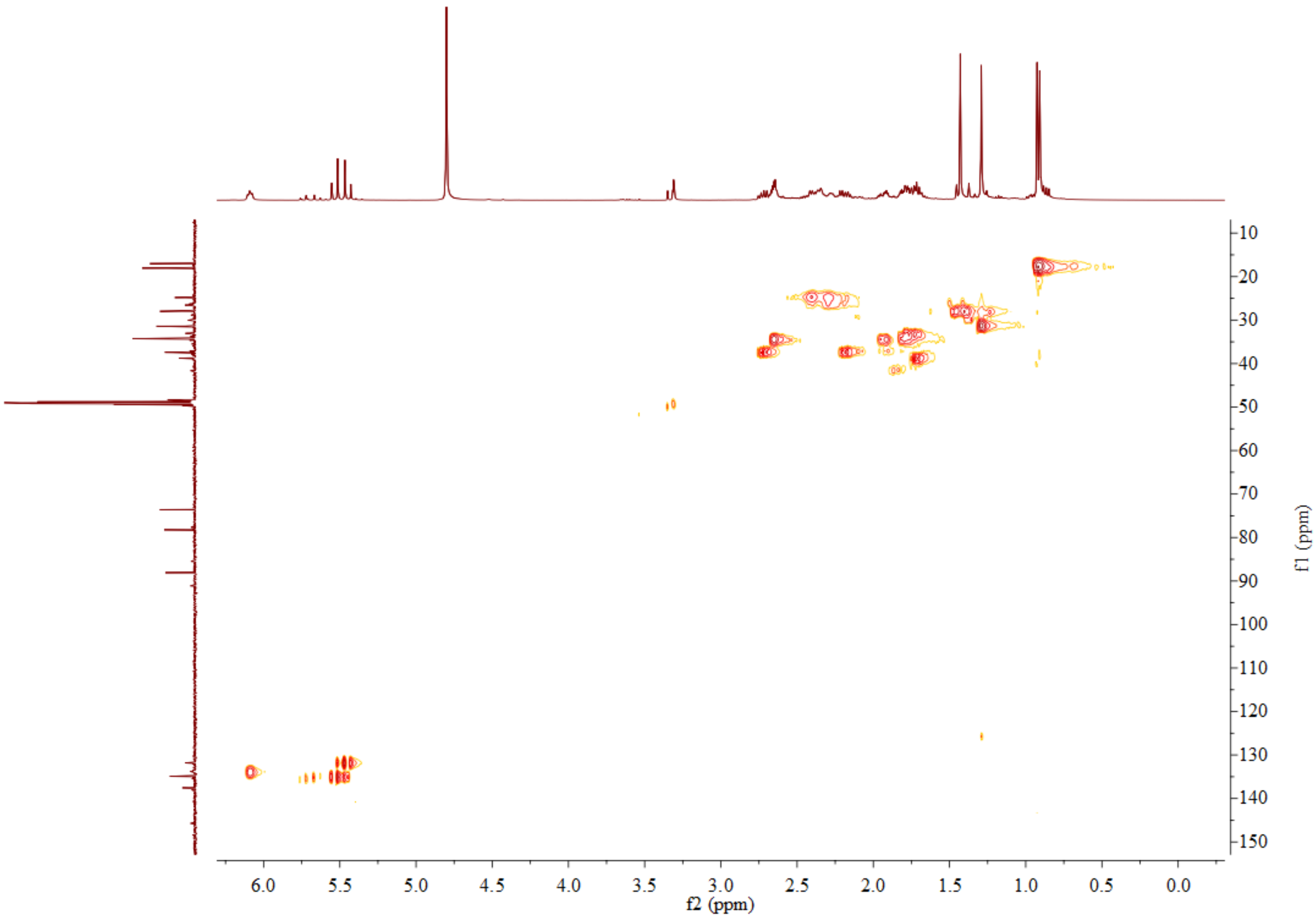

S25 HMBC spectrum of **4** in Methanol-d_4

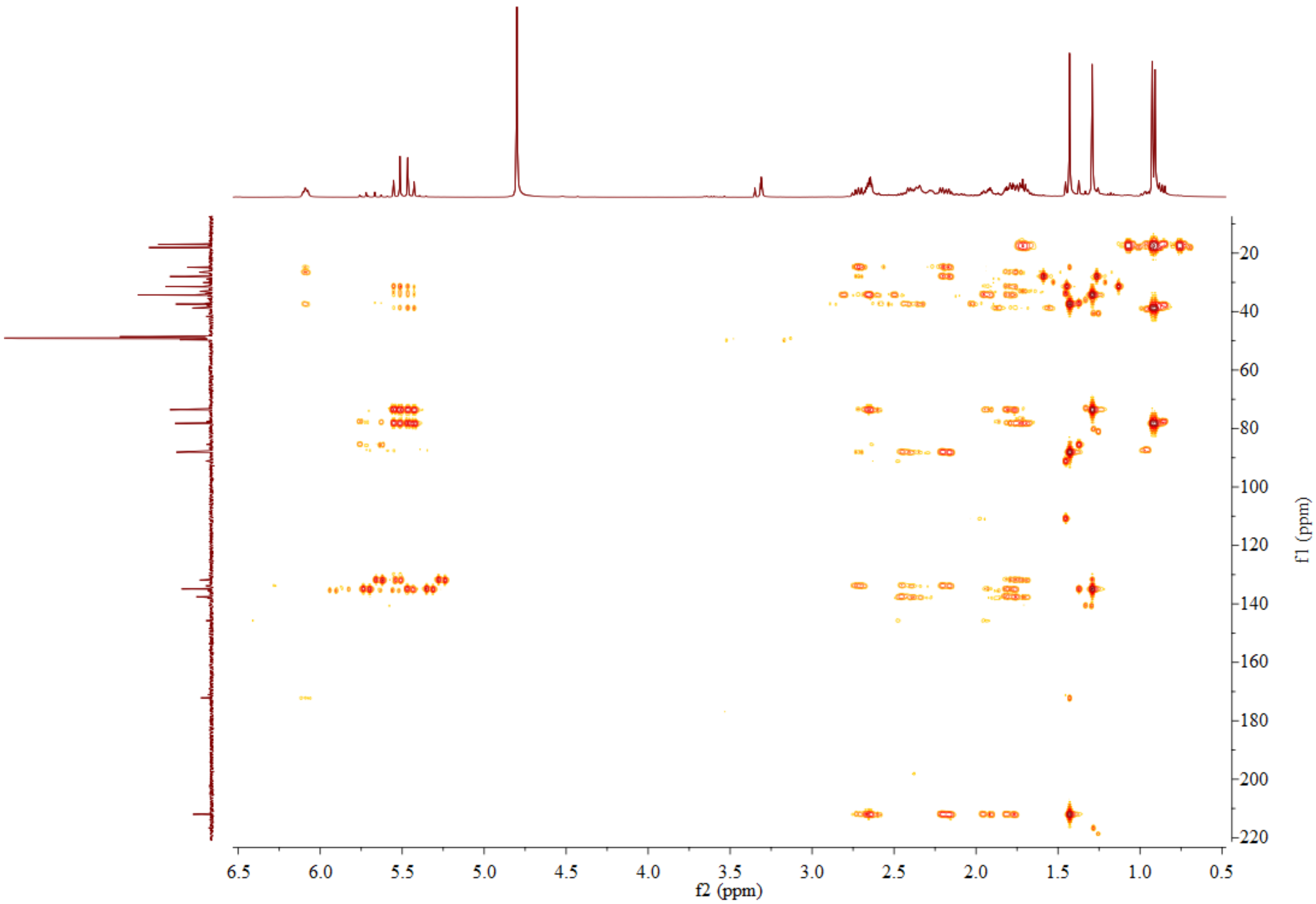

S26 ^{1}H–^{1}H COSY spectrum of **4** in Methanol-d_4

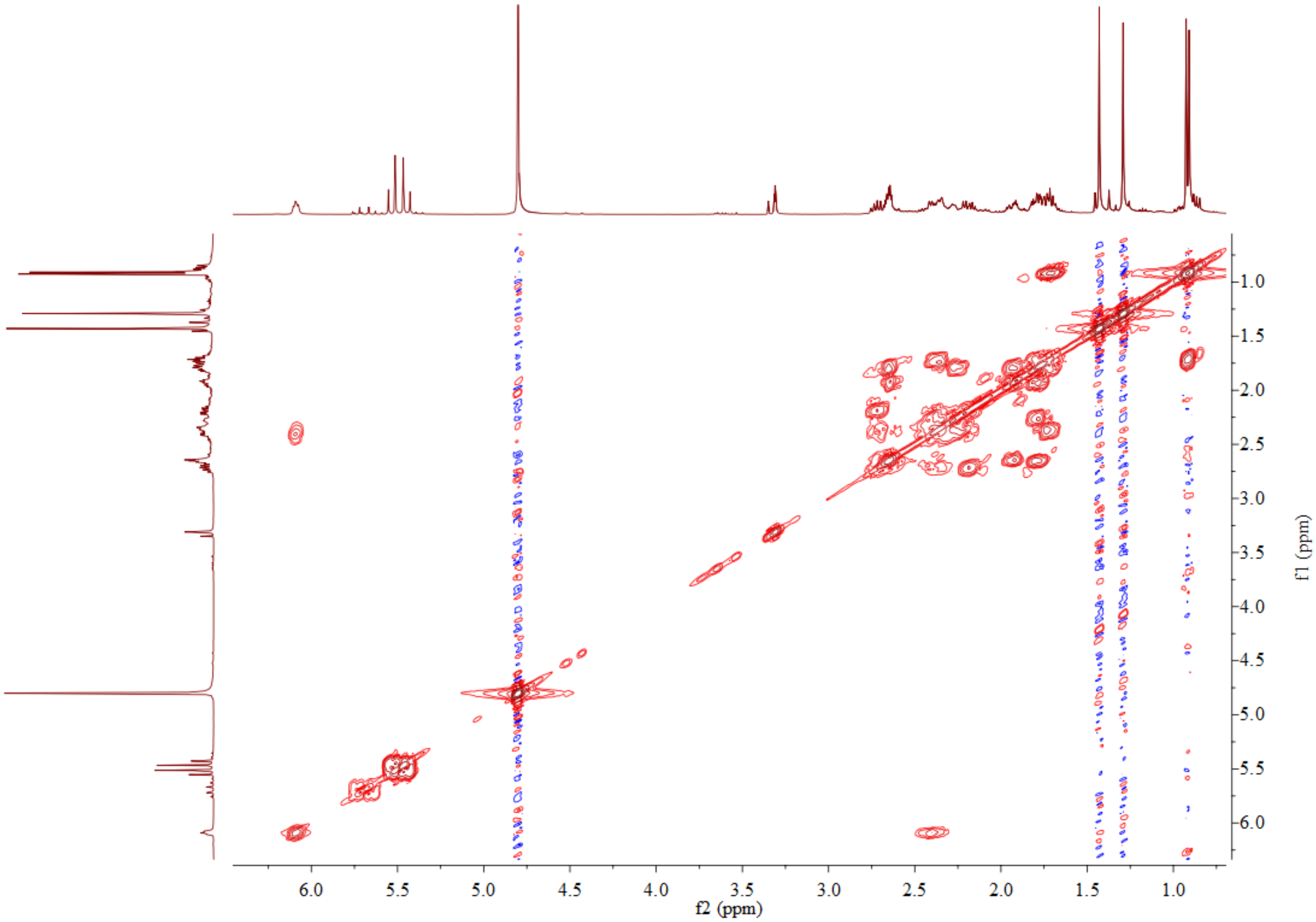

S27 NOESY spectrum of **4** in Methanol-d_4

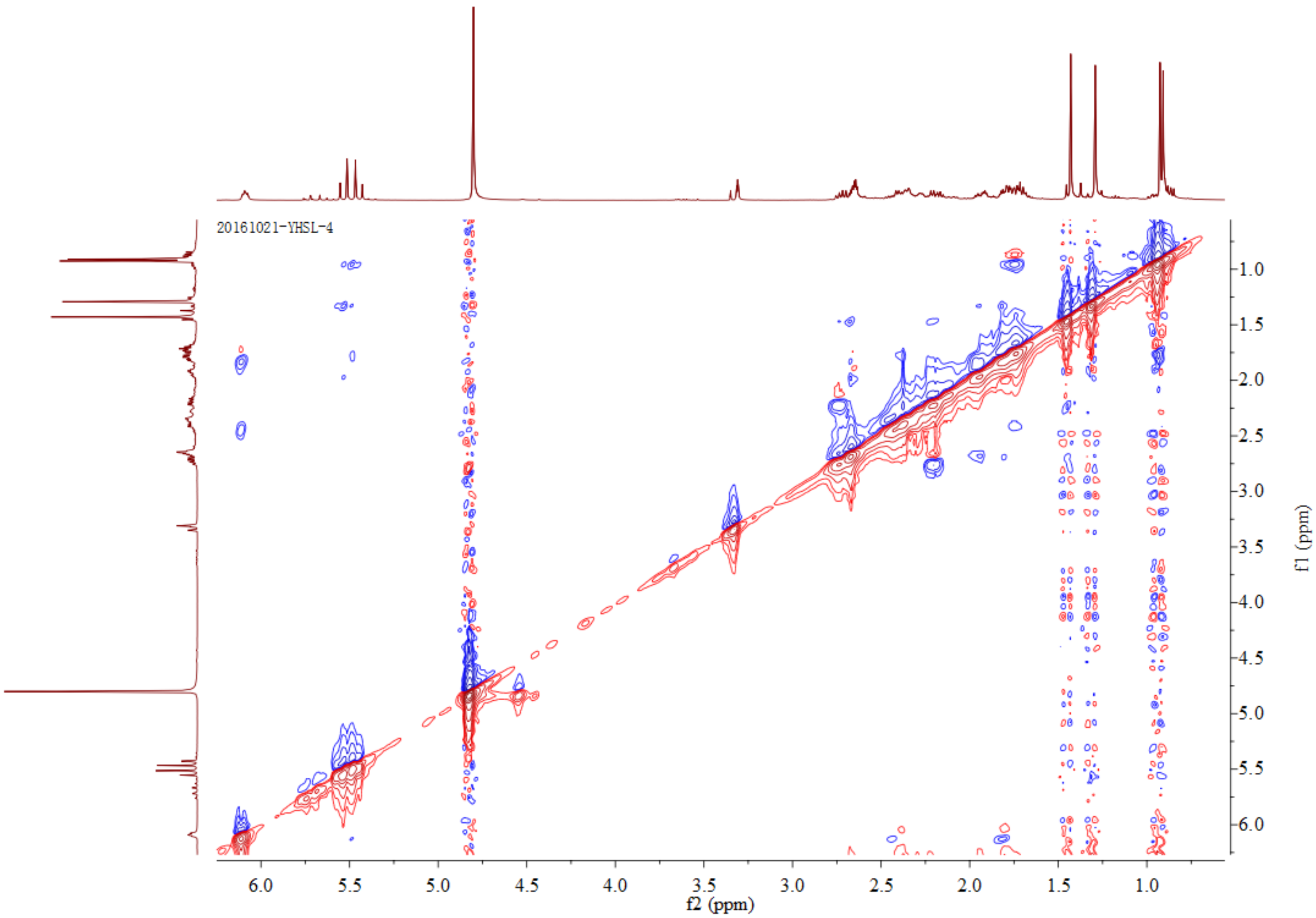

S28 HRESIMS spectrum of **4**

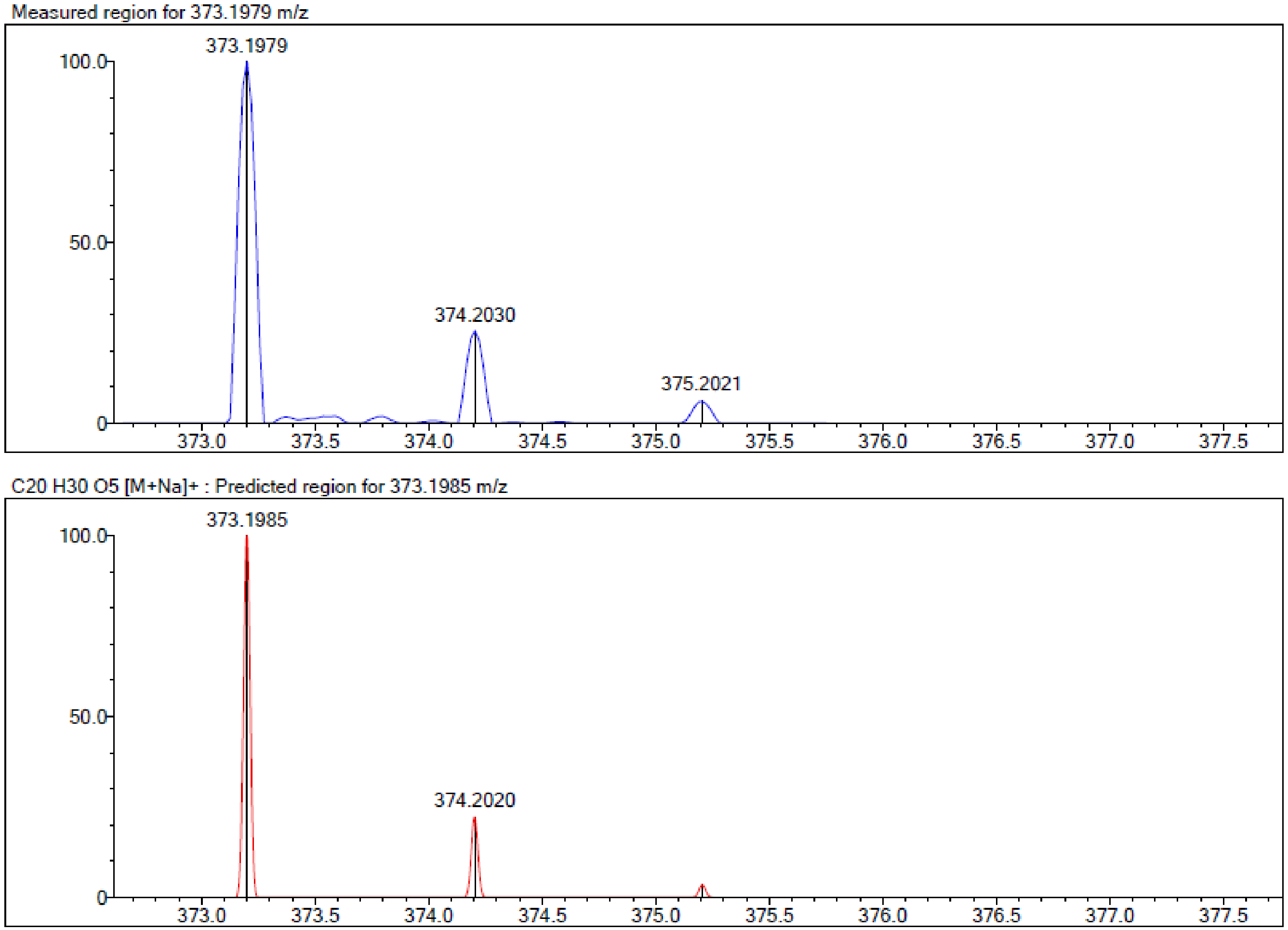

Rank	Score	Formula (M)	Ion	Meas. m/z	Pred. m/z	Df. (mDa)	Df. (ppm)	Iso	DBE
1	73.21	C20 H30 O5	[M+Na]+	373.1979	373.1985	-0.6	-1.61	74.35	6.0

S29 ^{1}H NMR spectrum of **5** in Methanol-d_4

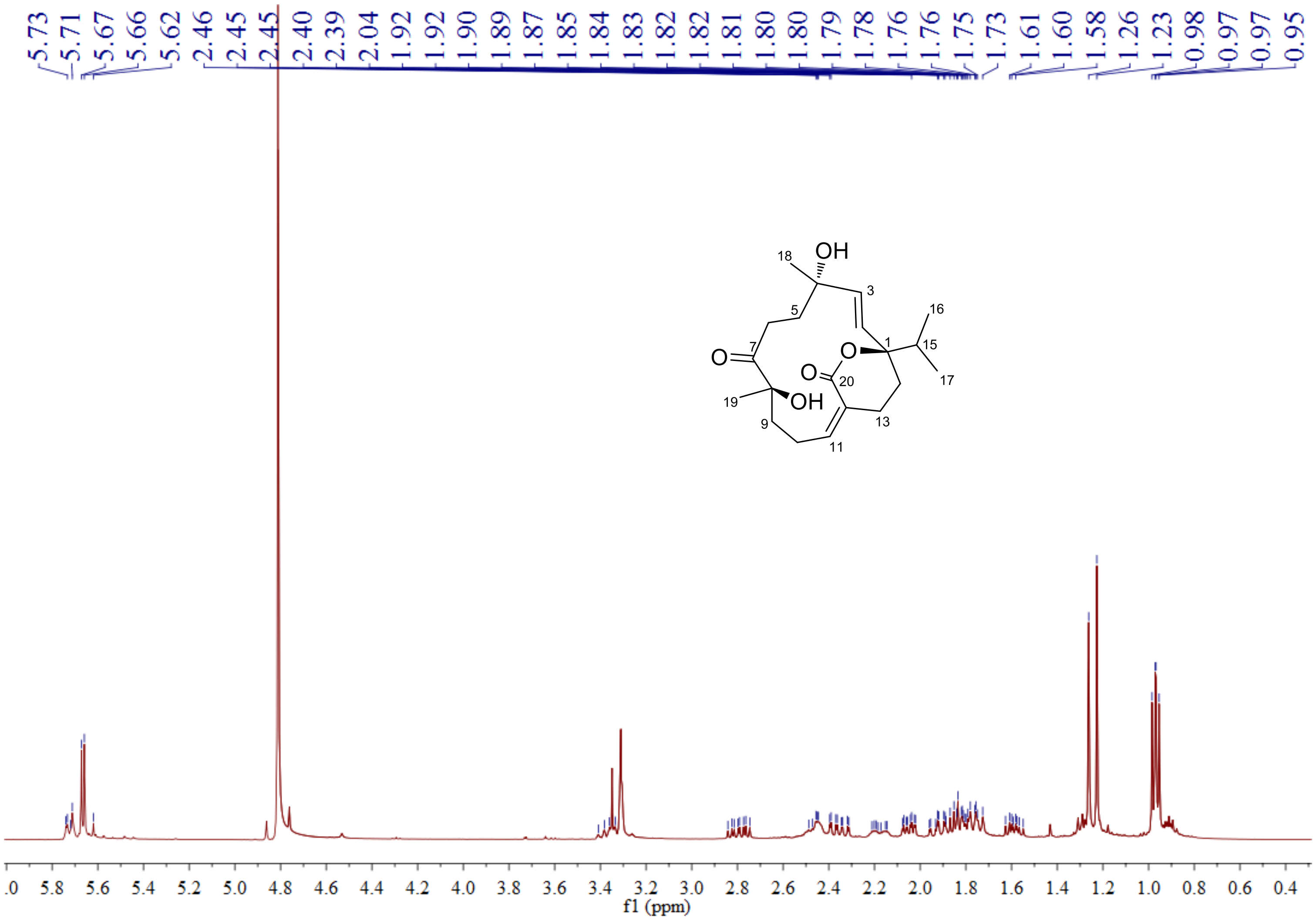

S30 ^{13}C NMR spectrum of **5** in Methanol-d_4

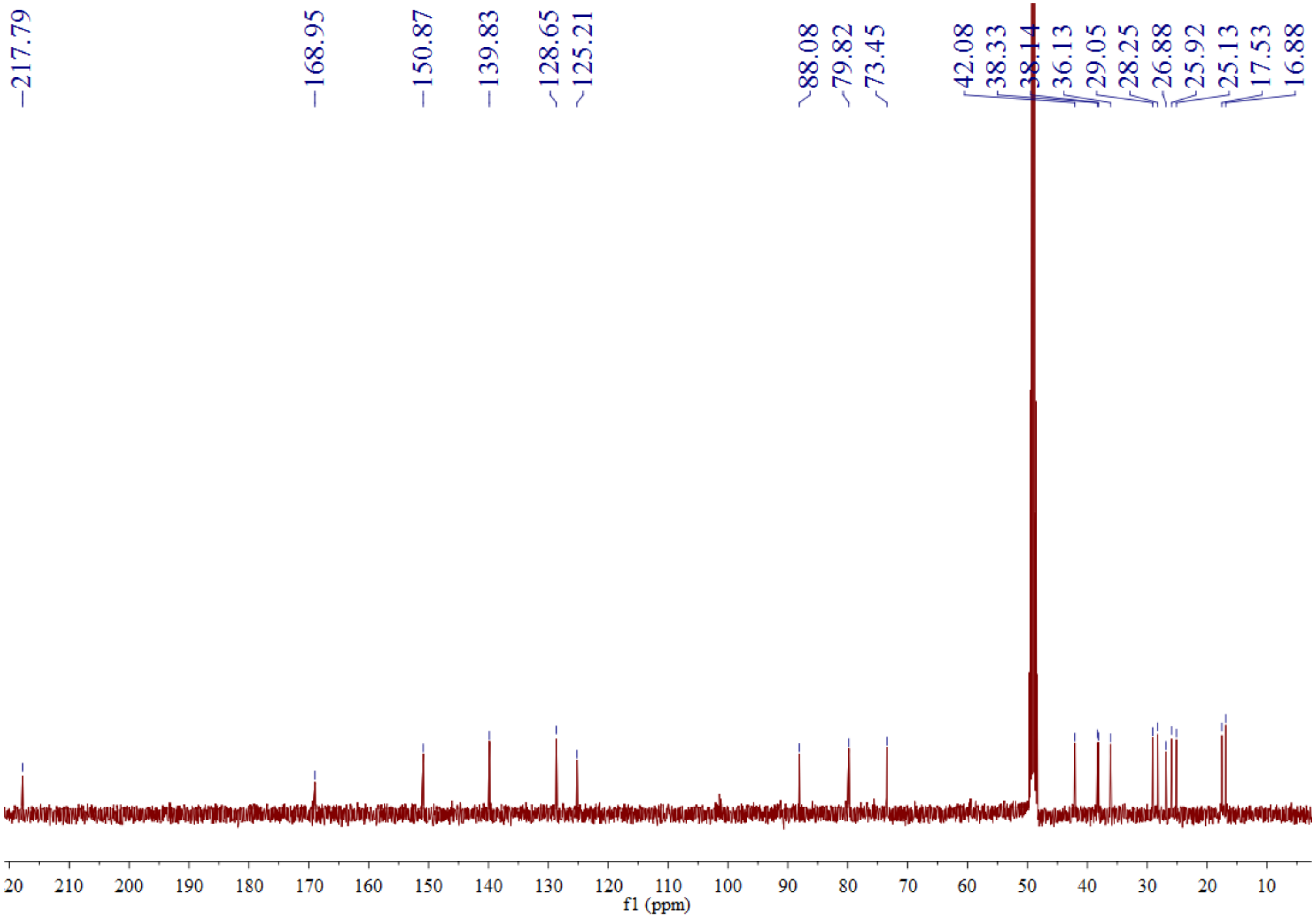

S31 HSQC spectrum of **5** in Methanol-d_4

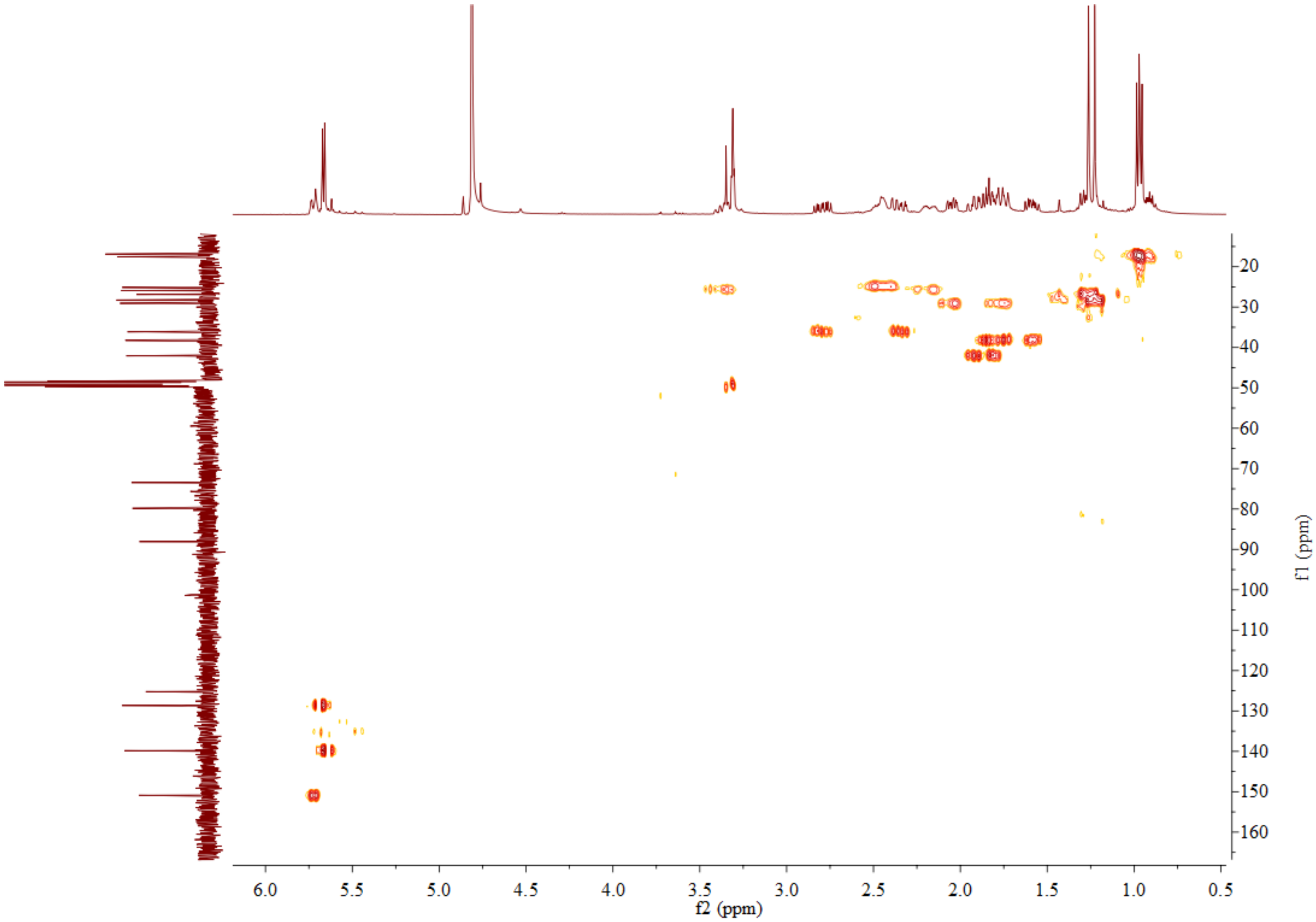

S32 HMBC spectrum of **5** in Methanol-d_4

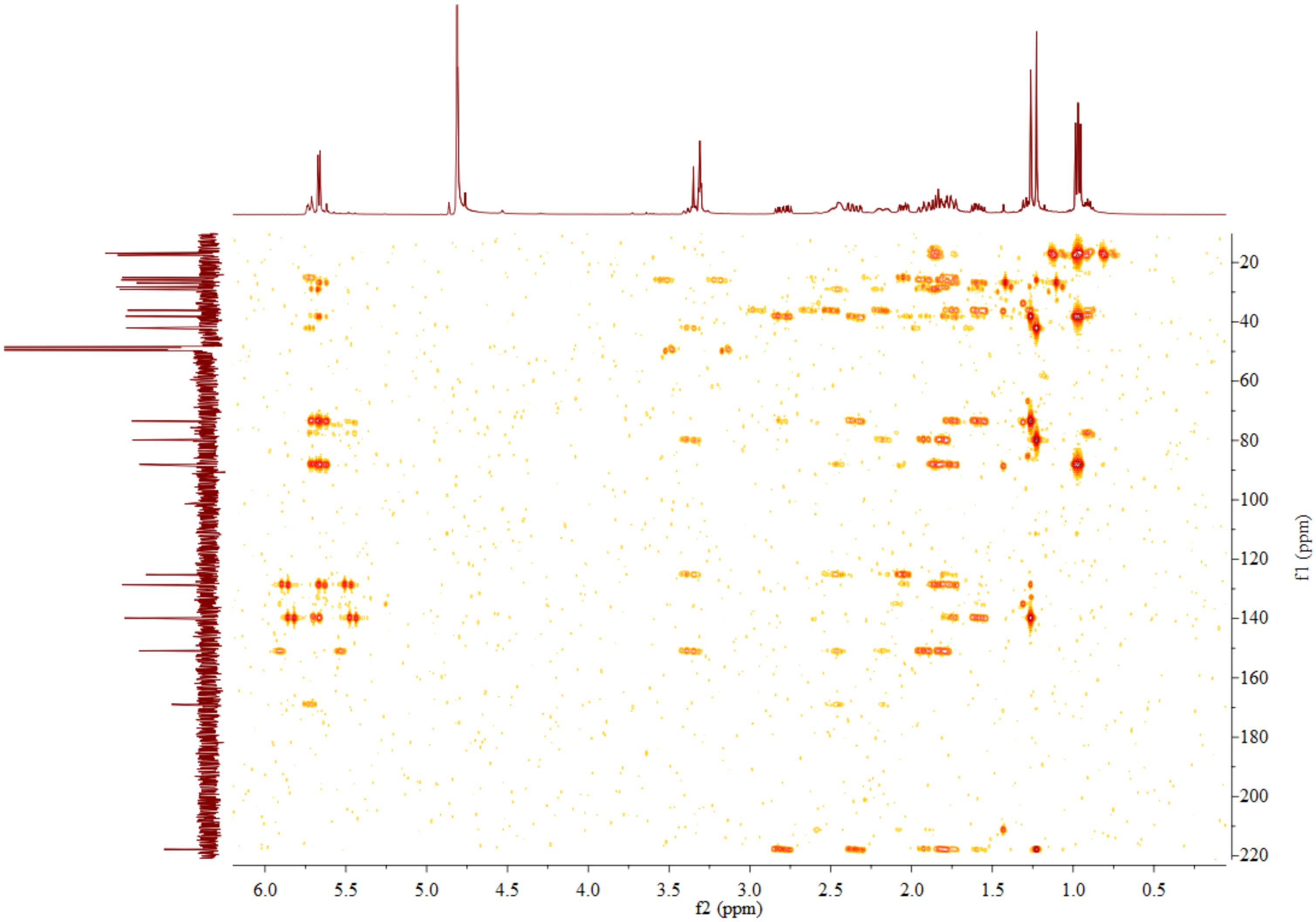

S33 ^{1}H–^{1}H COSY spectrum of **5** in Methanol-d_4

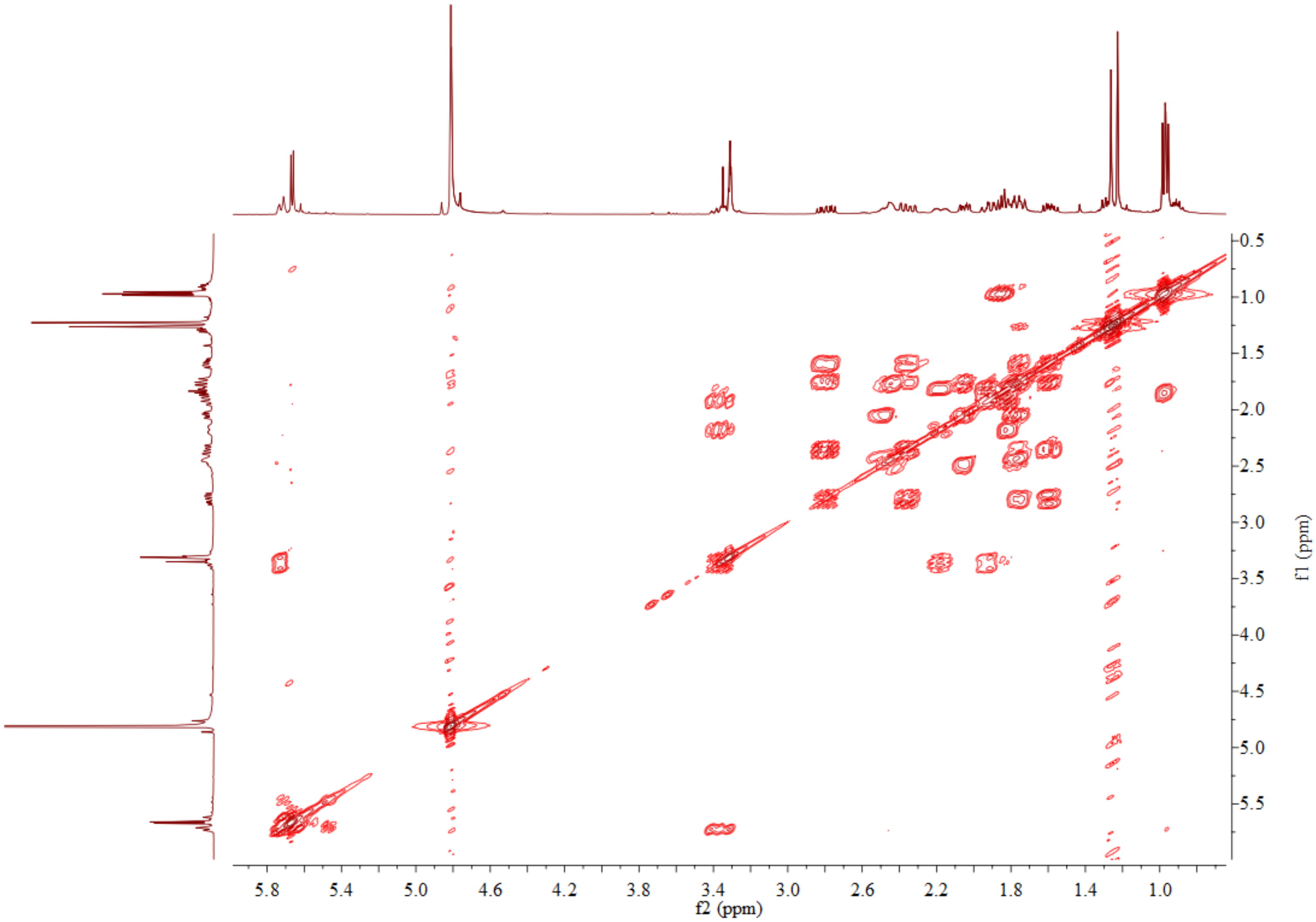

S34 NOESY spectrum of **5** in Methanol-d_4

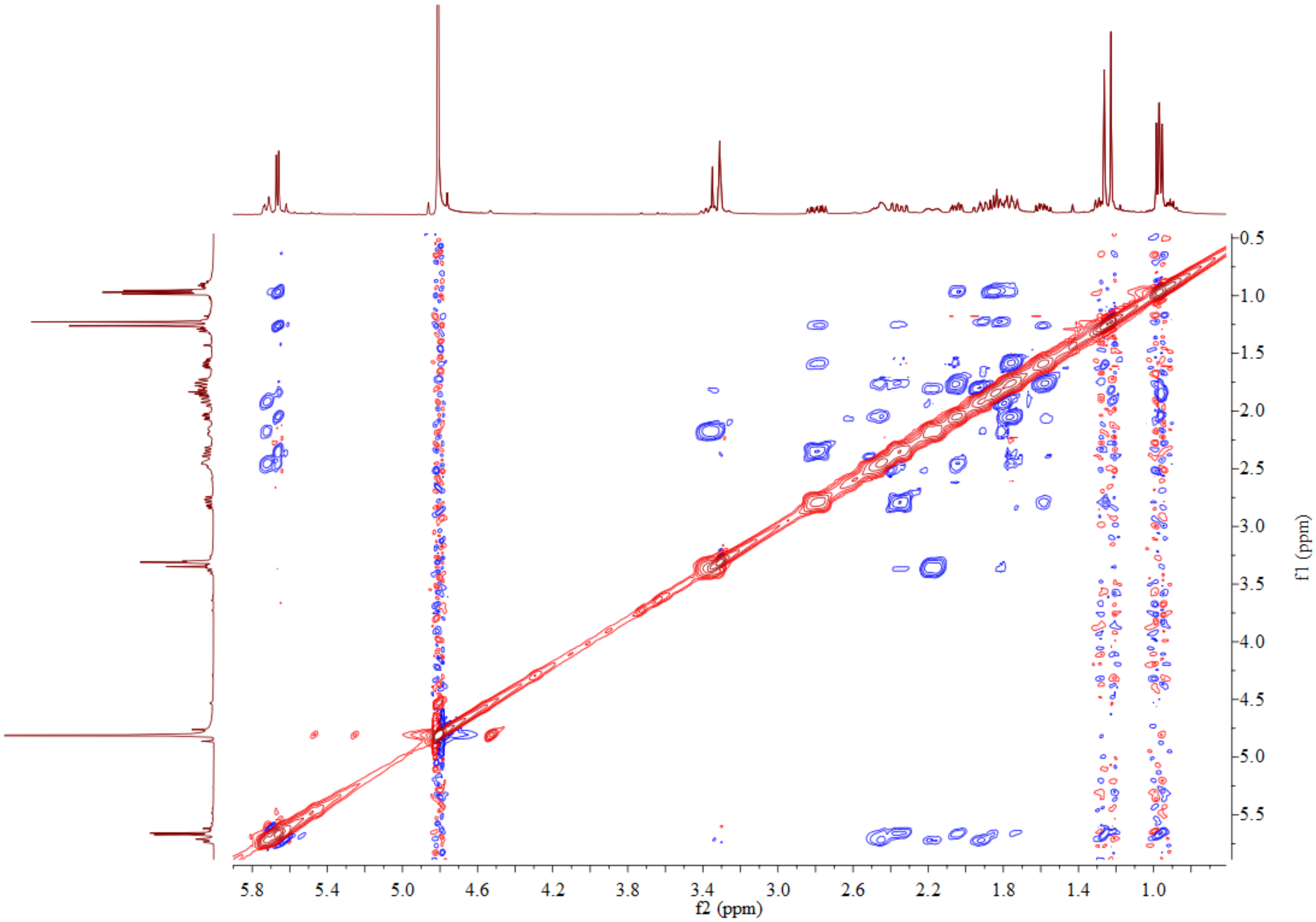

S35 HRESIMS spectrum of **5**

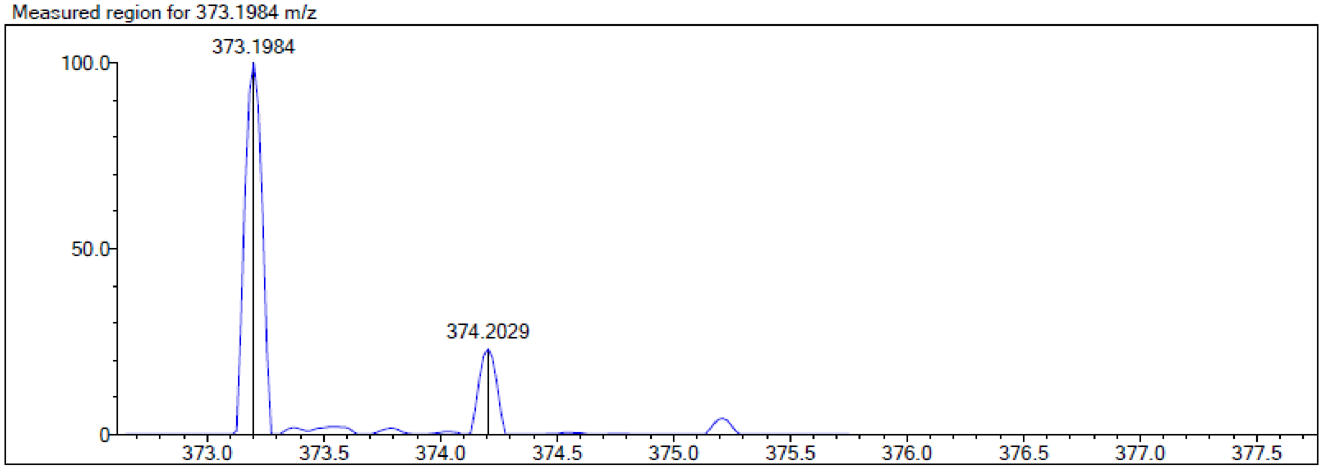

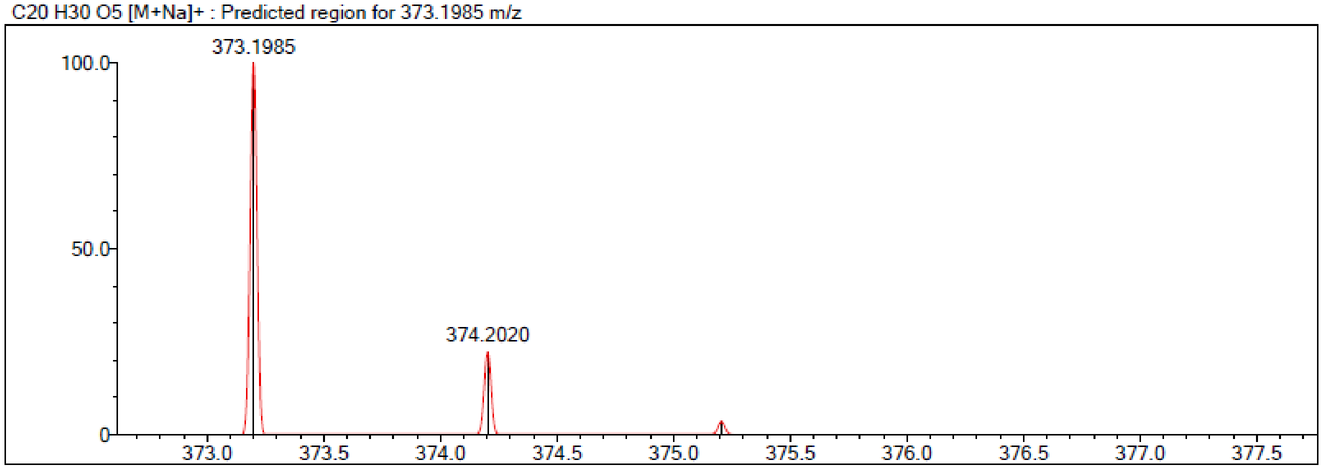

Rank	Score	Formula (M)	Ion	Meas. m/z	Pred. m/z	Df. (mDa)	Df. (ppm)	Iso	DBE
1	87.48	C20 H30 O5	[M+Na]+	373.1984	373.1985	-0.1	-0.27	87.48	6.0

S36 ^{1}H NMR spectrum of **6** in Methanol-d_4

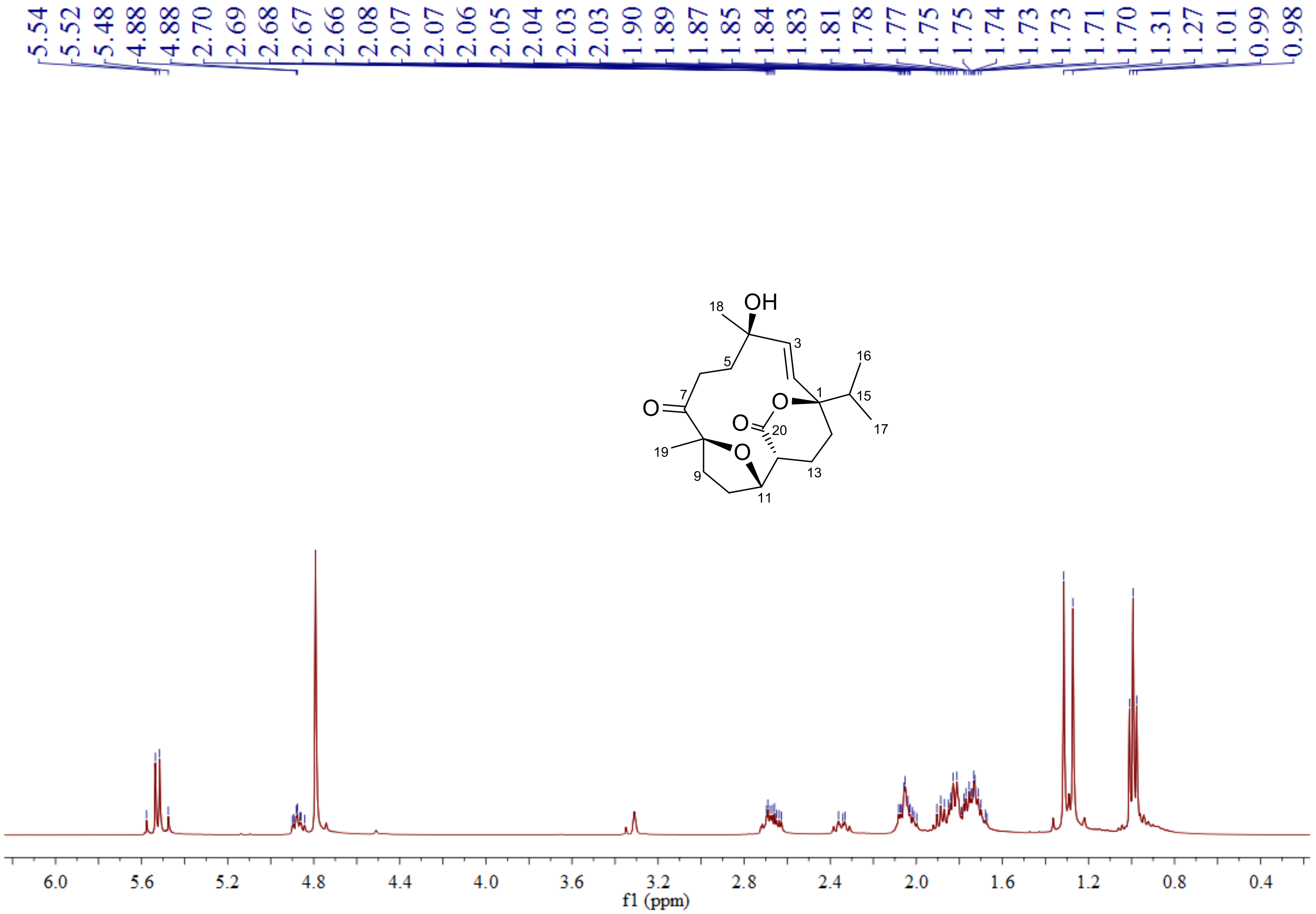

S37 ^{13}C NMR spectrum of **6** in Methanol-d_4

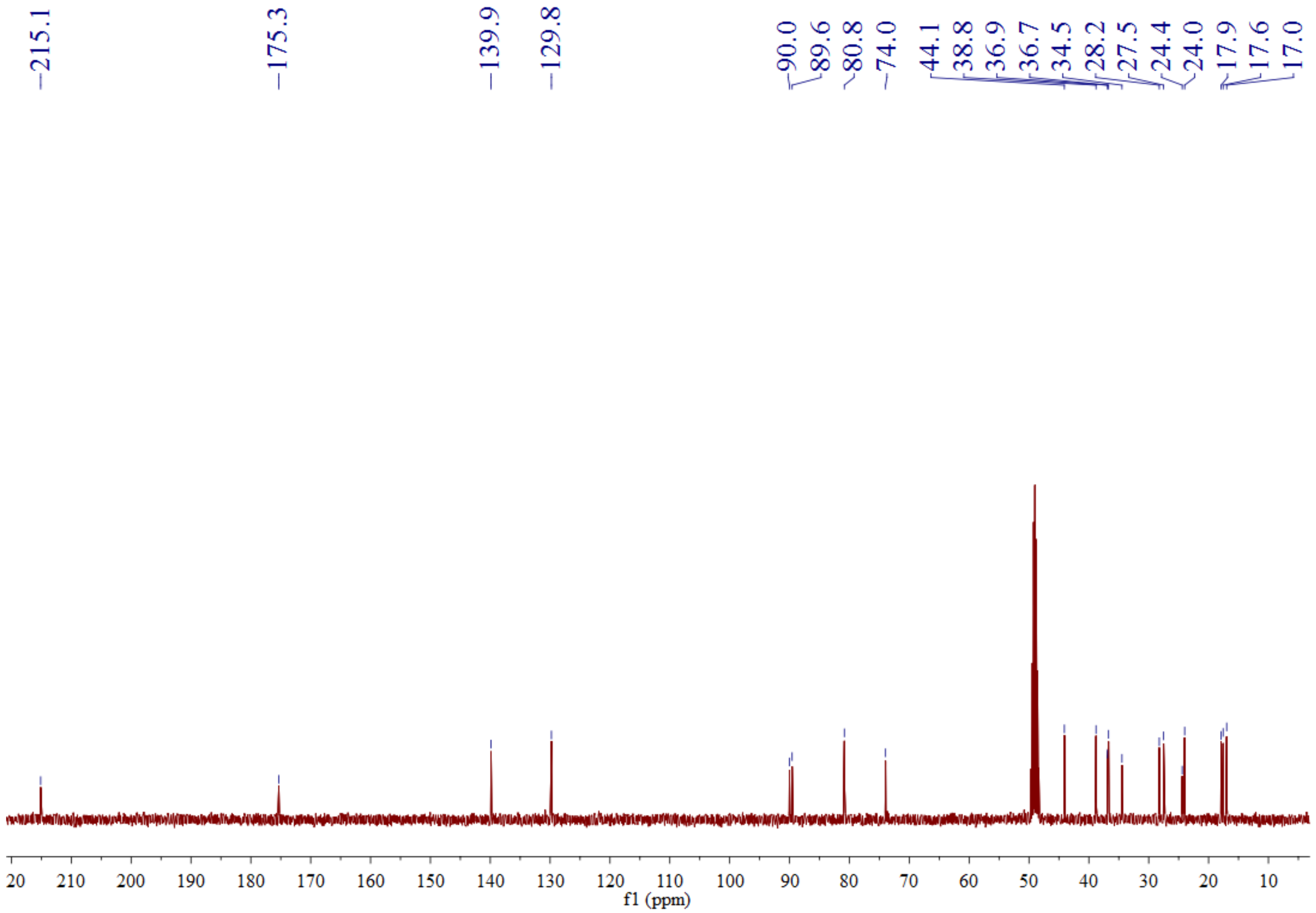

S38 HSQC spectrum of **6** in Methanol-d_4

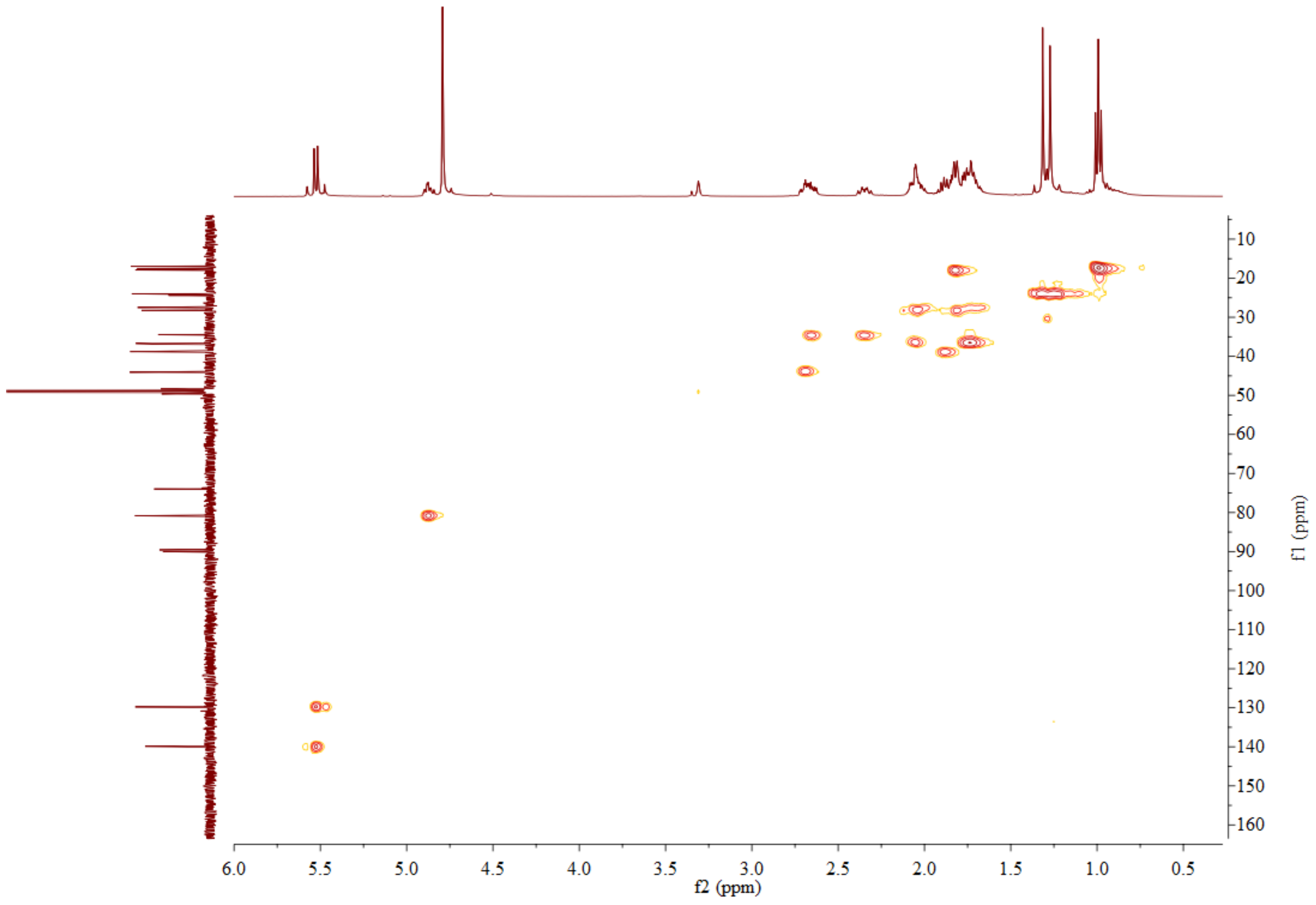

S39 HMBC spectrum of **6** in Methanol-d_4

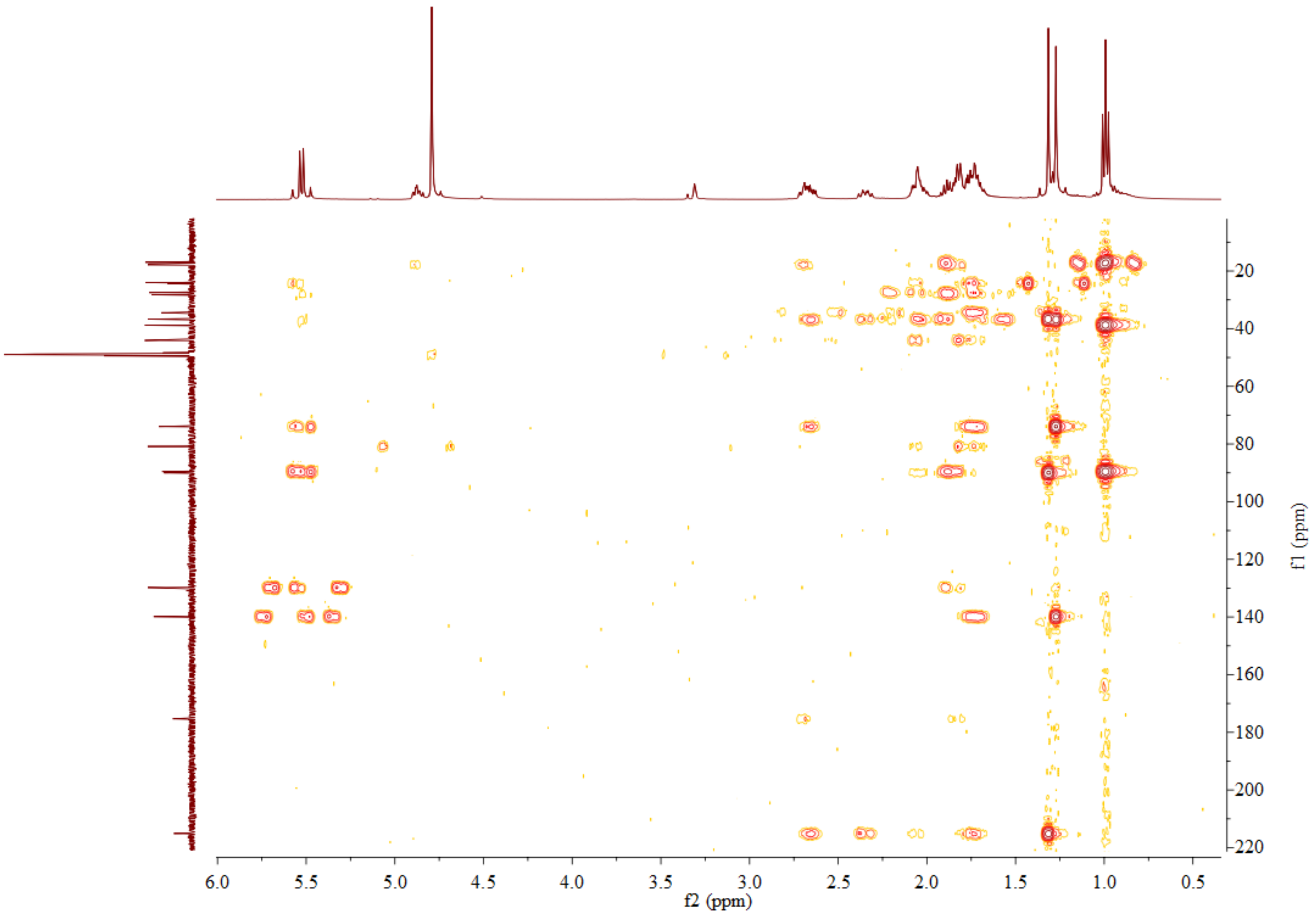

S40 ^{1}H–^{1}H COSY spectrum of **6** in Methanol-d_4

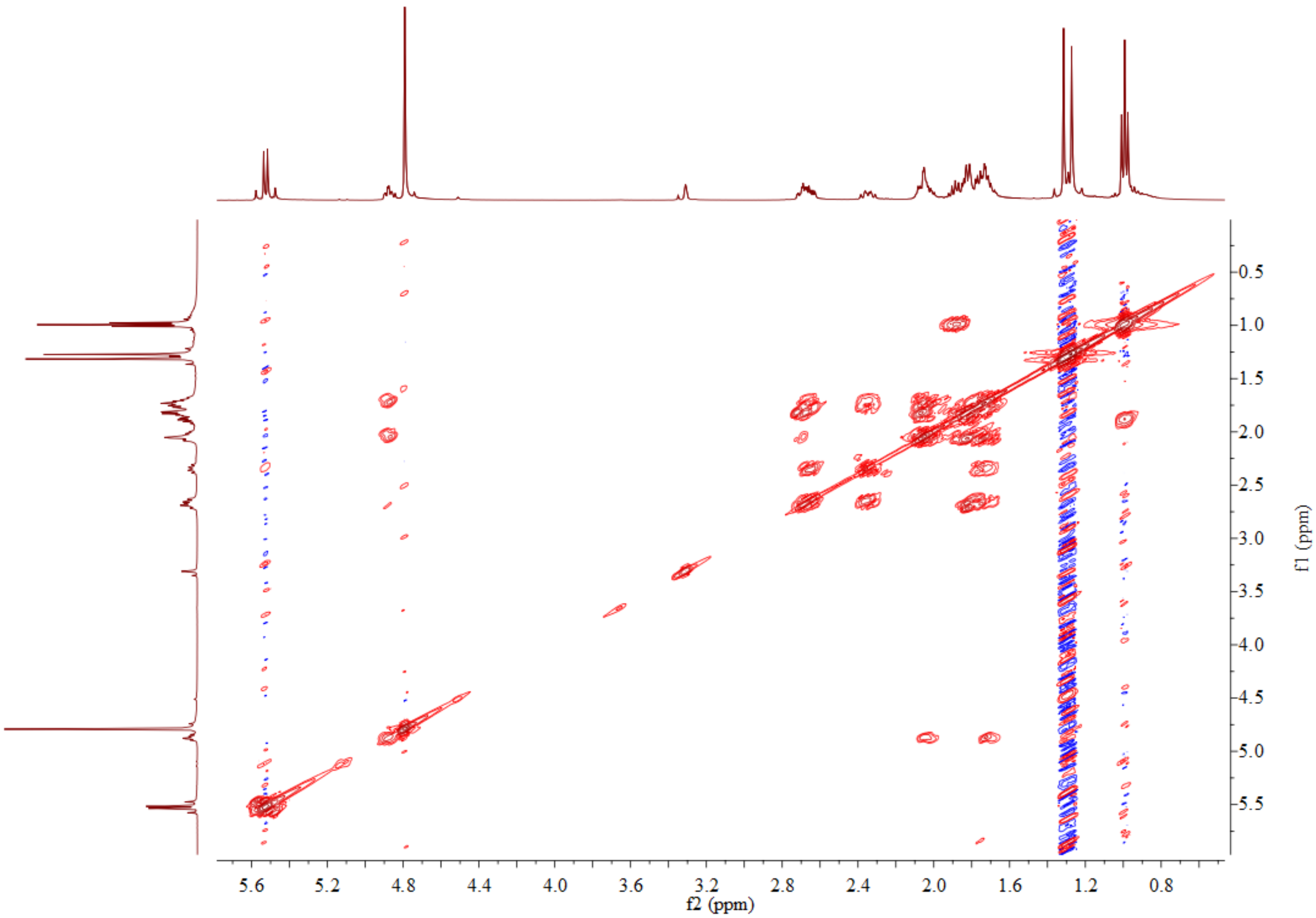

S41 NOESY spectrum of **6** in Methanol-d_4

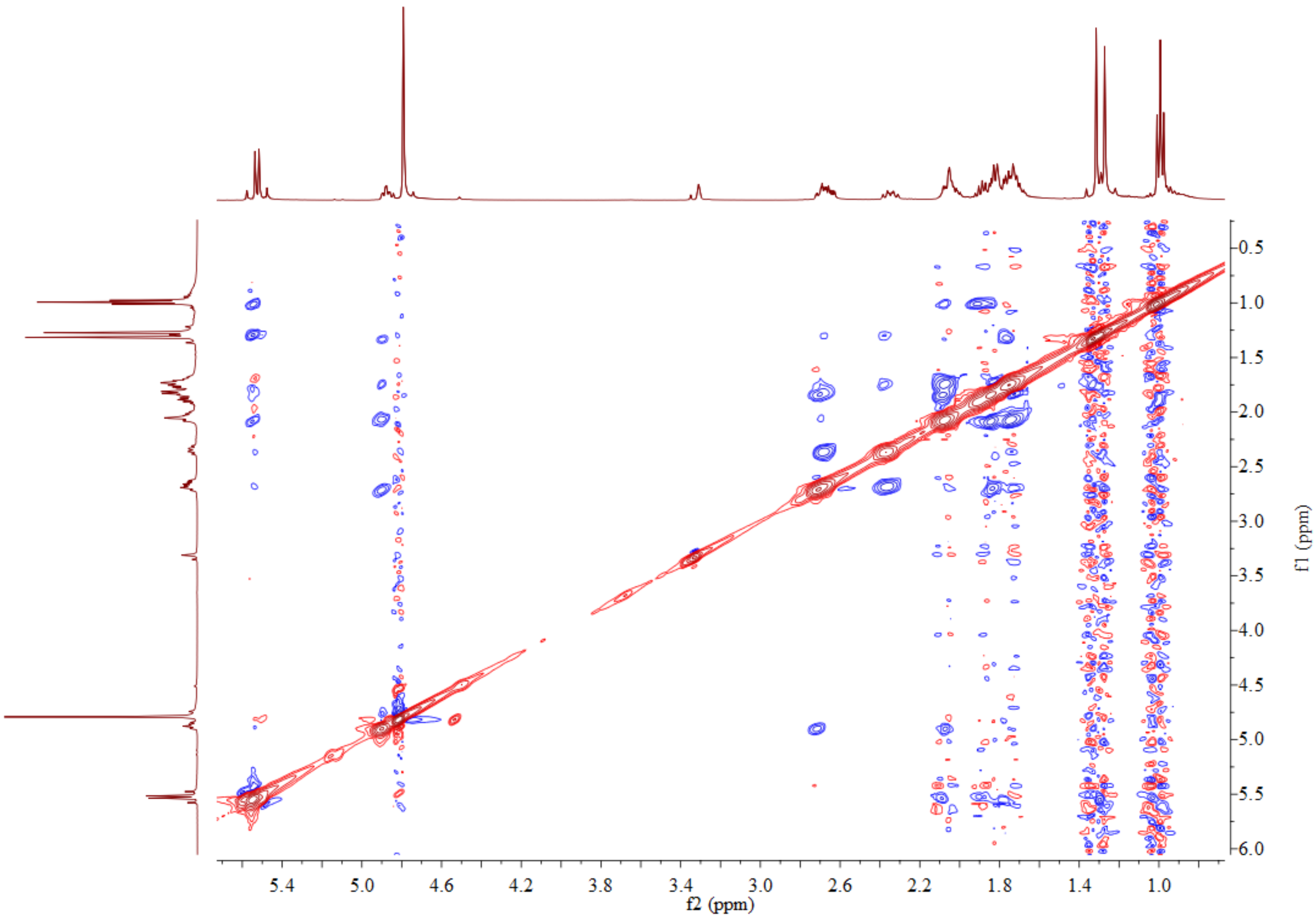

S42 HRESIMS spectrum of **6**

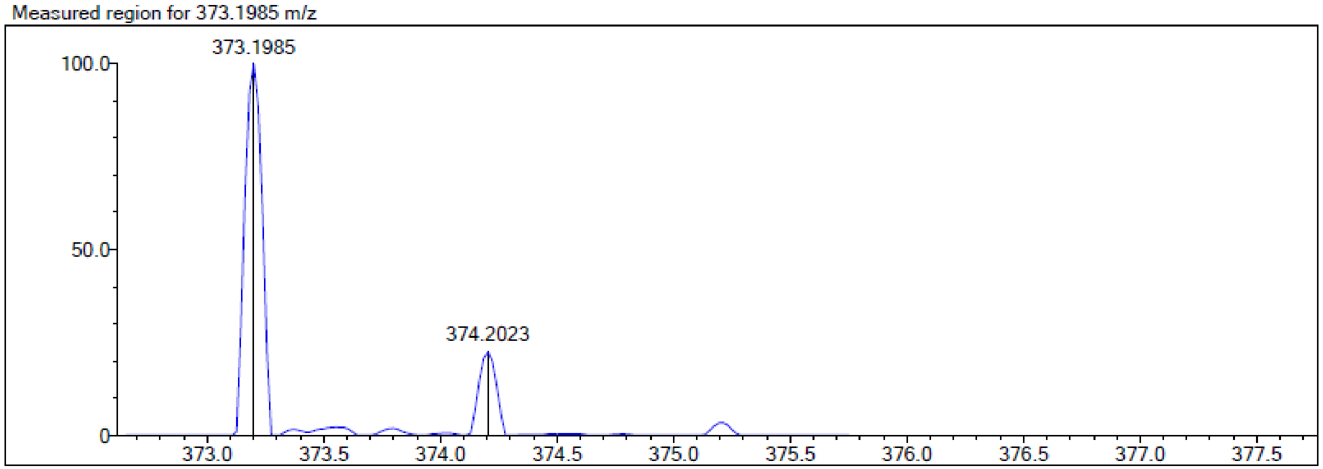

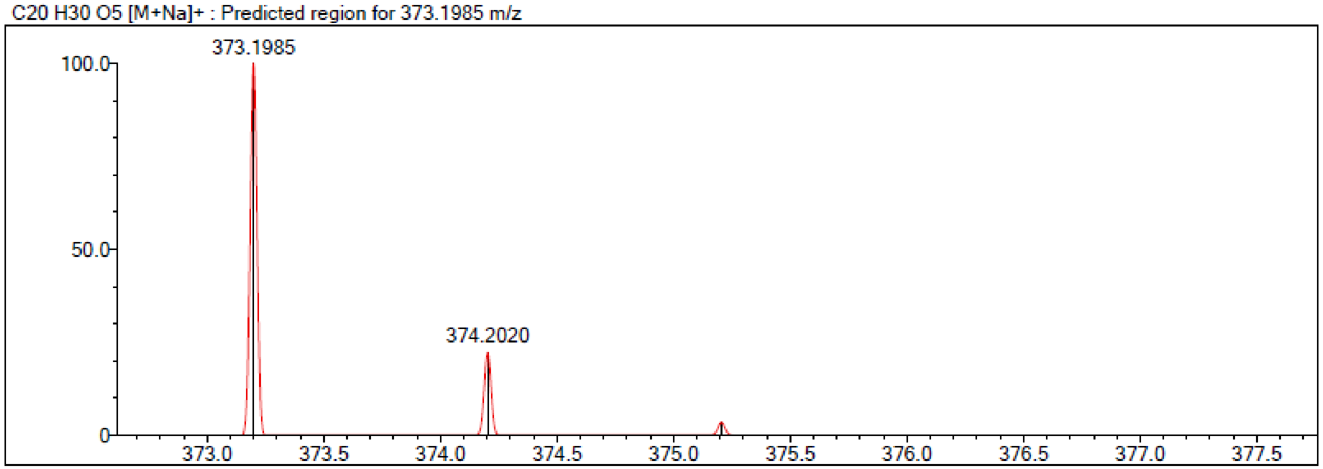

Rank	Score	Formula (M)	Ion	Meas. m/z	Pred. m/z	Df. (mDa)	Df. (ppm)	Iso	DBE
1	88.02	C20 H30 O5	[M+Na]+	373.1985	373.1985	-0.0	0.00	88.02	6.0

S43 ^{1}H NMR spectrum of **7** in $CDCl_3$

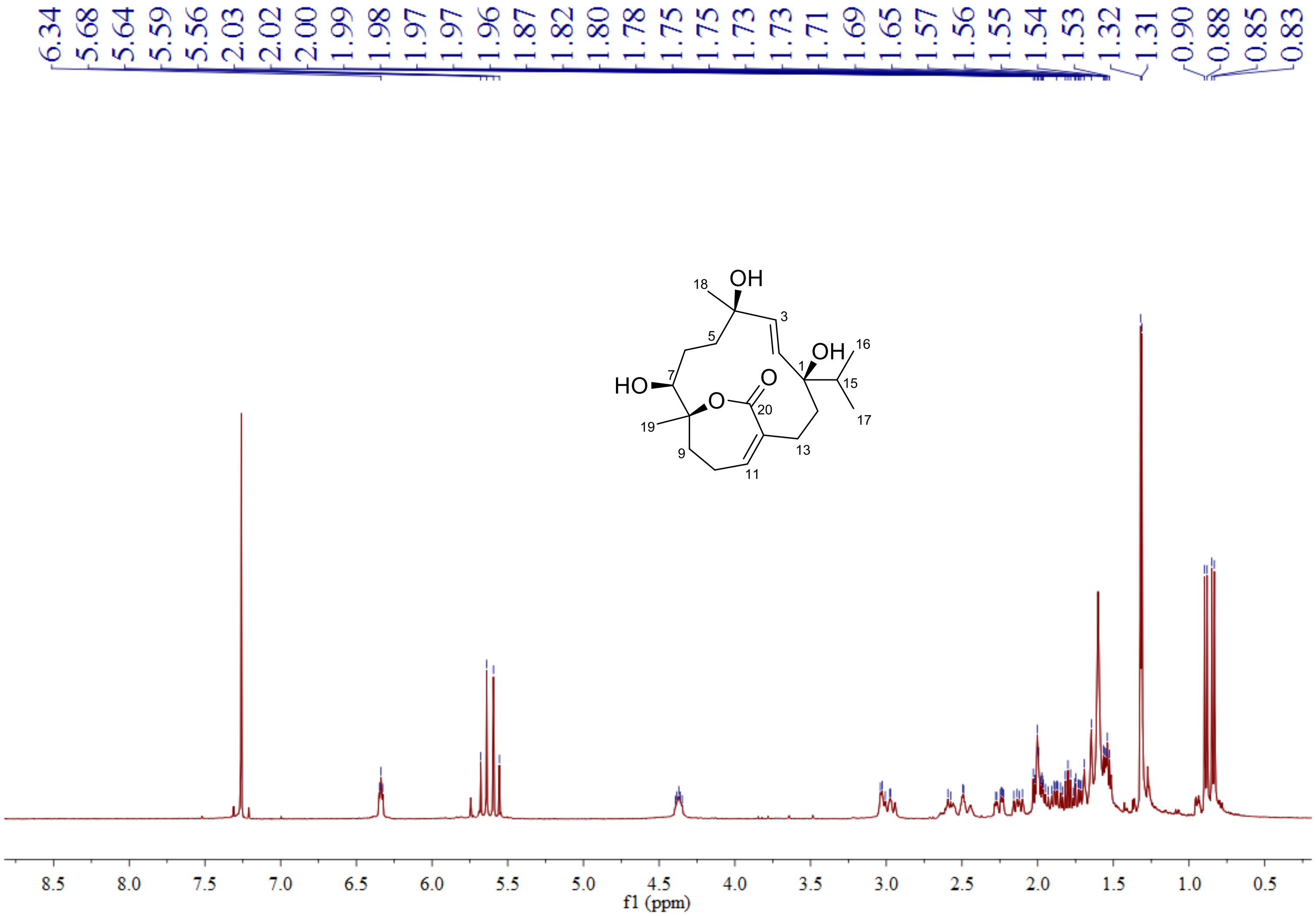

S44 ^{13}C NMR spectrum of **7** in $CDCl_3$

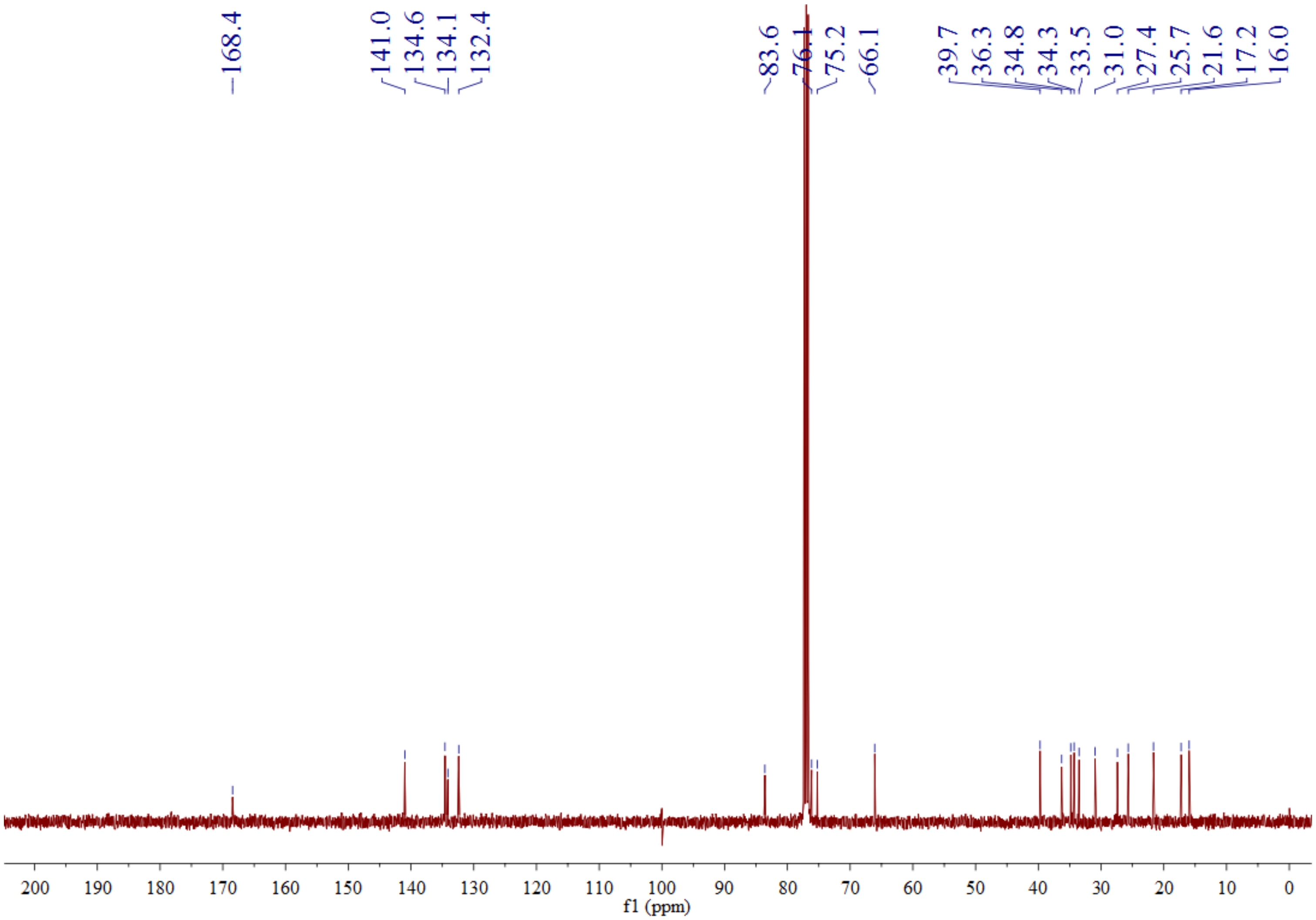

S45 HSQC spectrum of **7** in $CDCl_3$

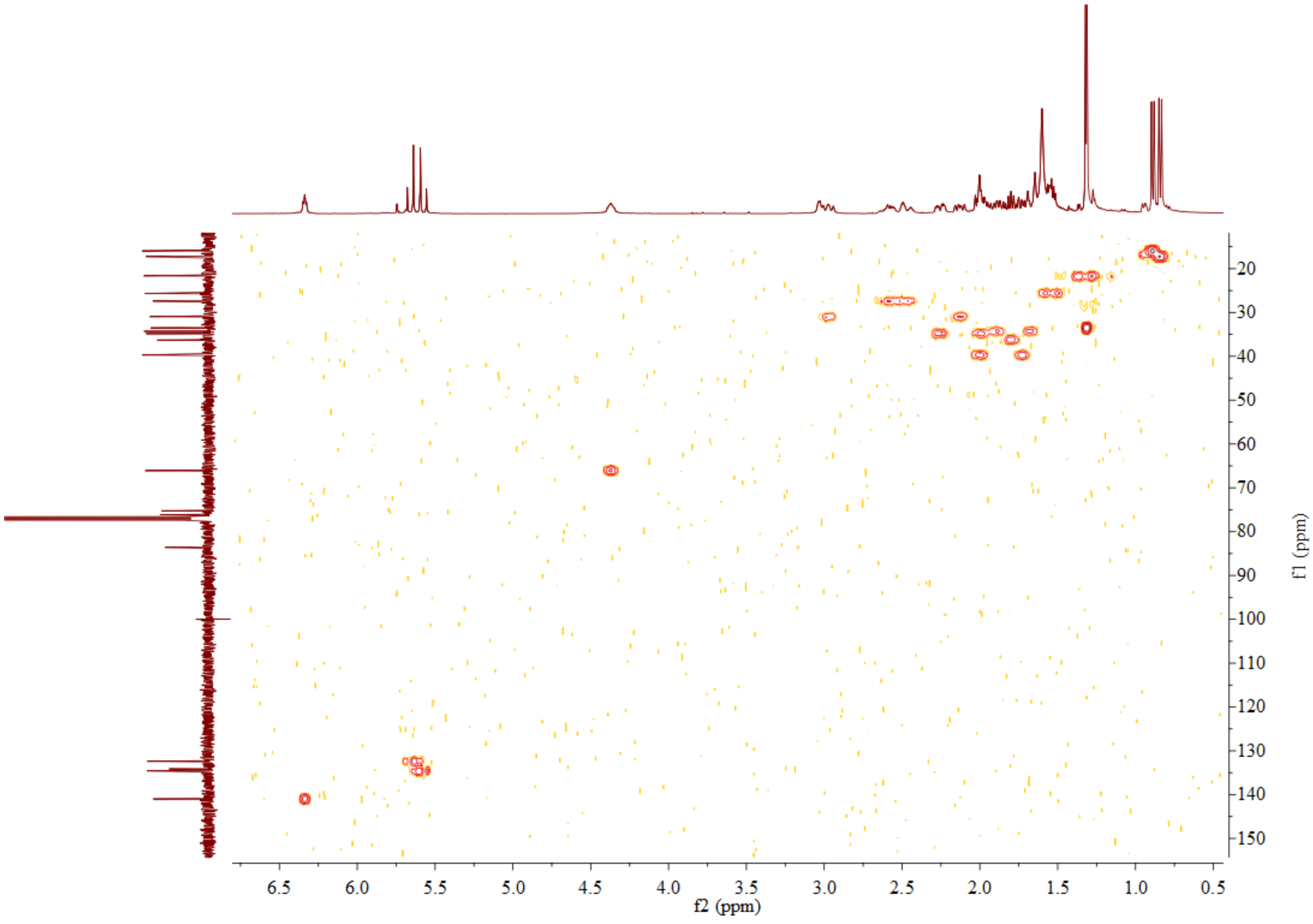

S46 HMBC spectrum of **7** in $CDCl_3$

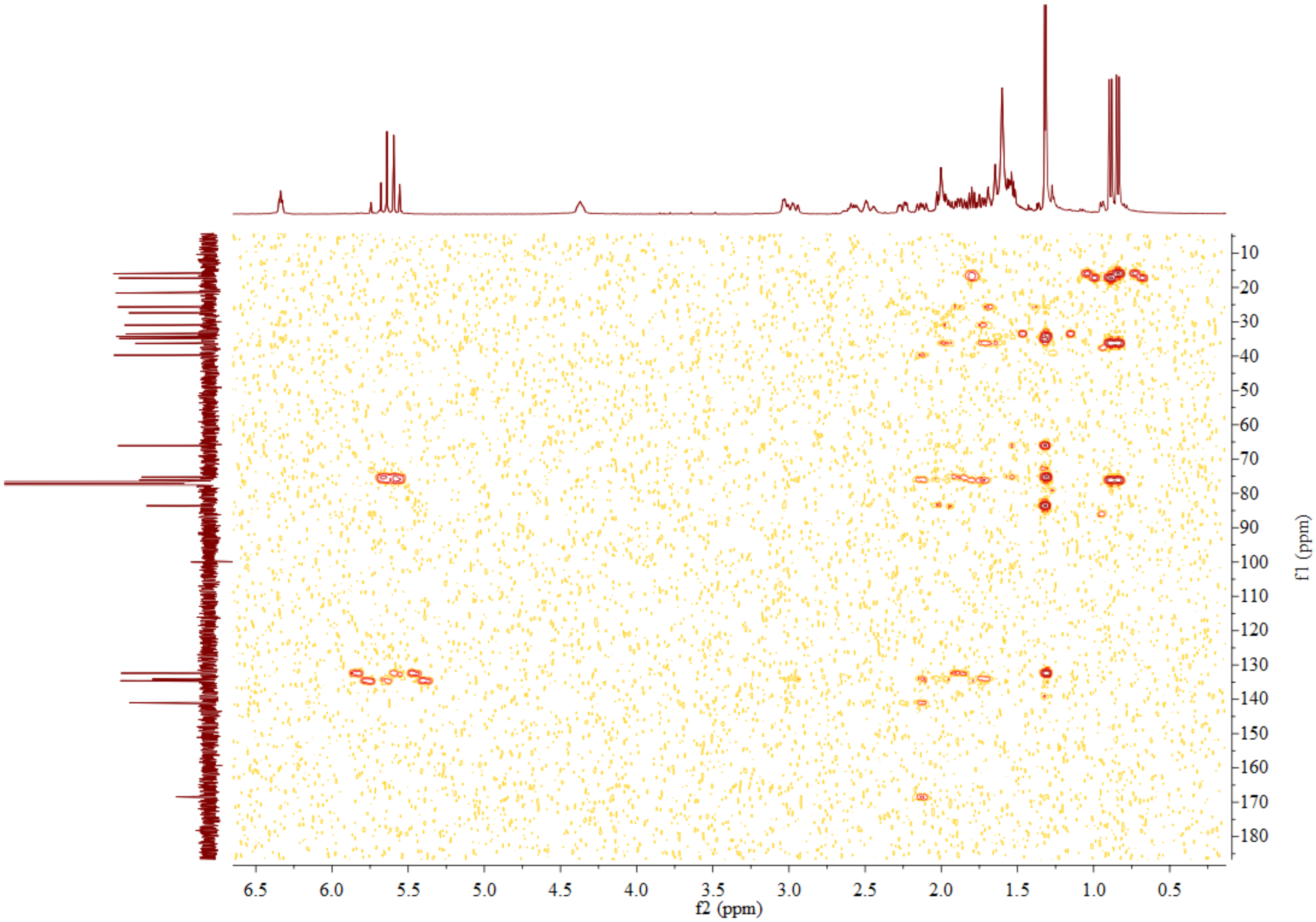

S47 ^{1}H–^{1}H COSY spectrum of **7** in $CDCl_3$

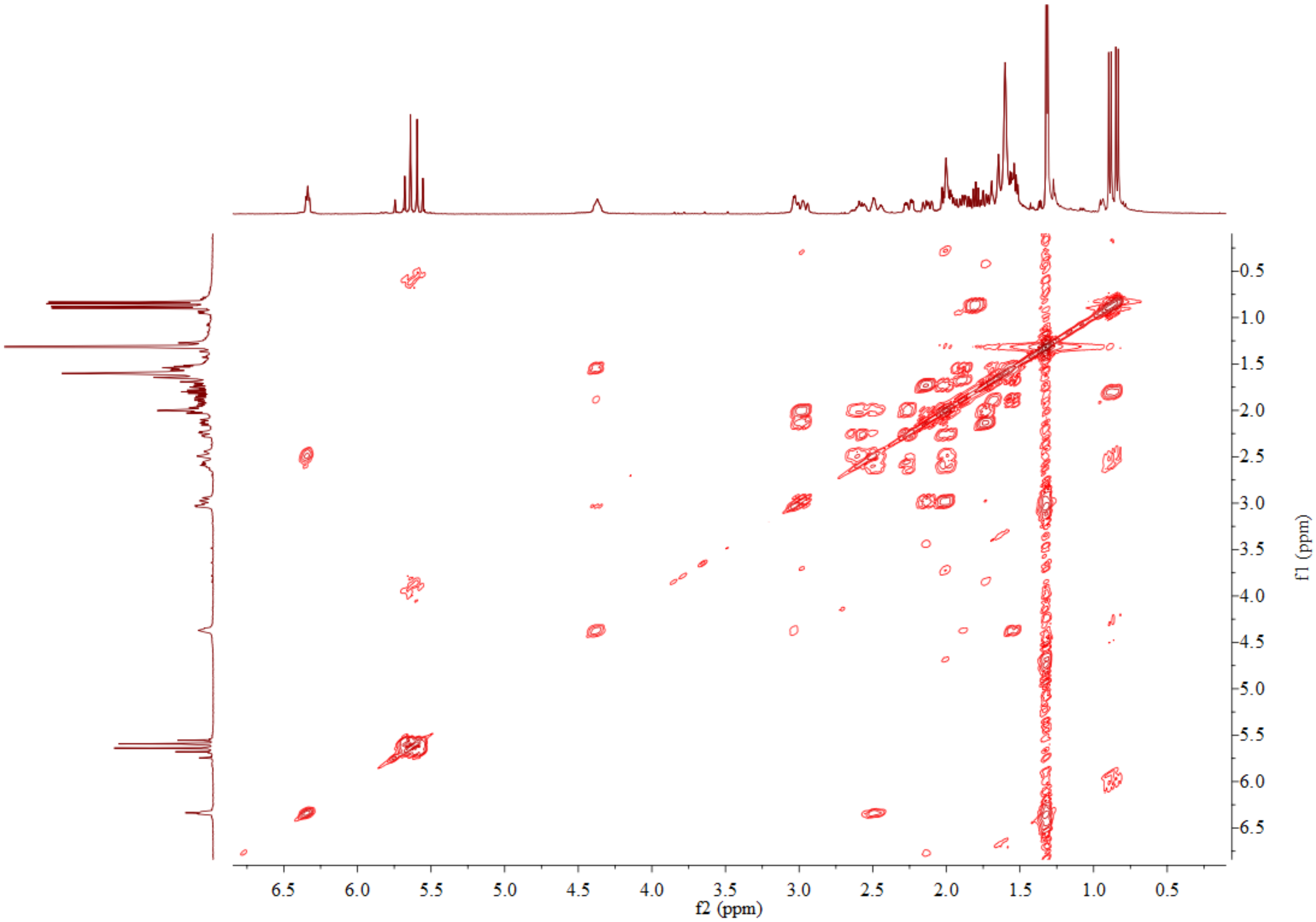

S48 NOESY spectrum of **7** in $CDCl_3$

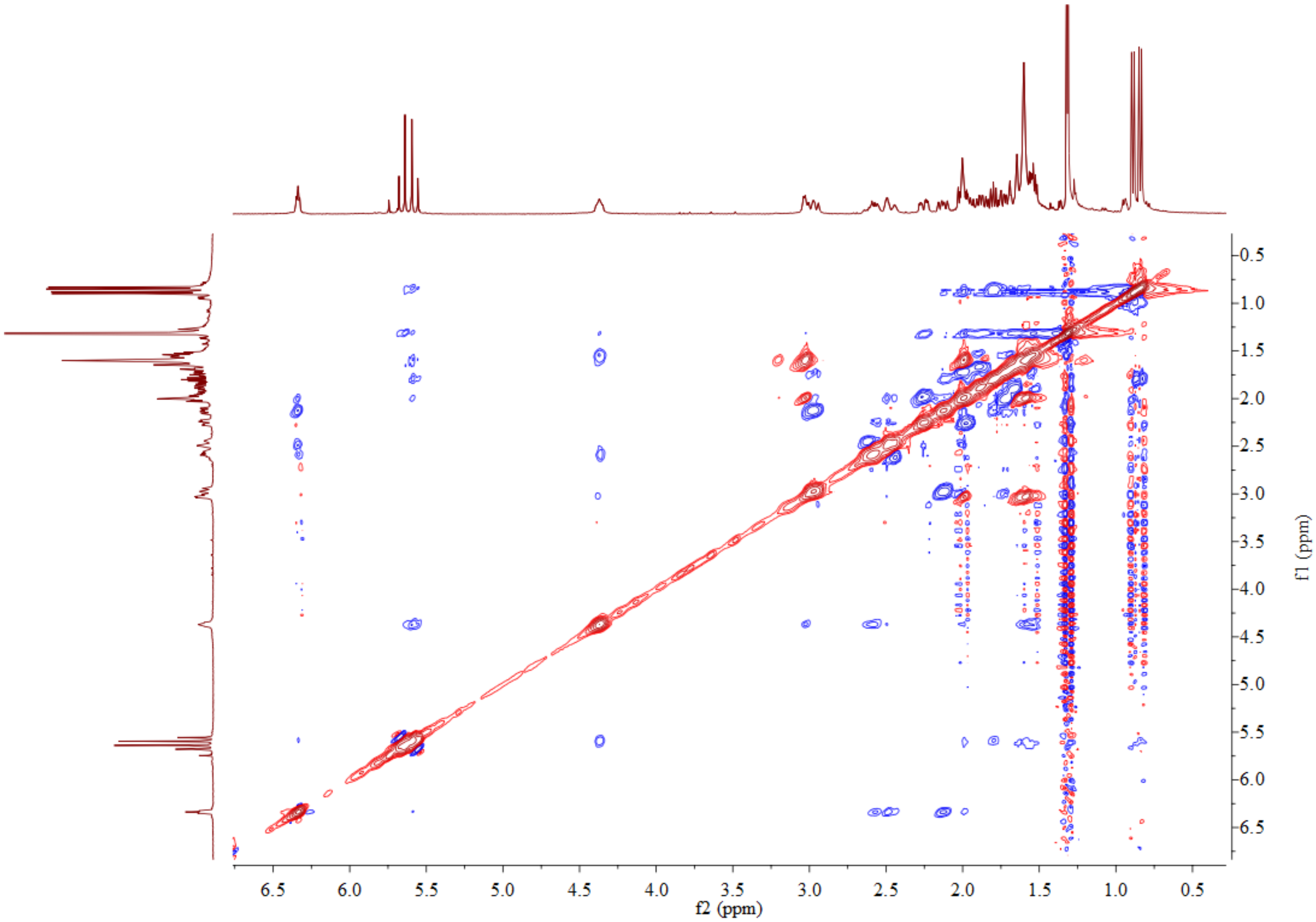

S49 HRESIMS spectrum of **7**

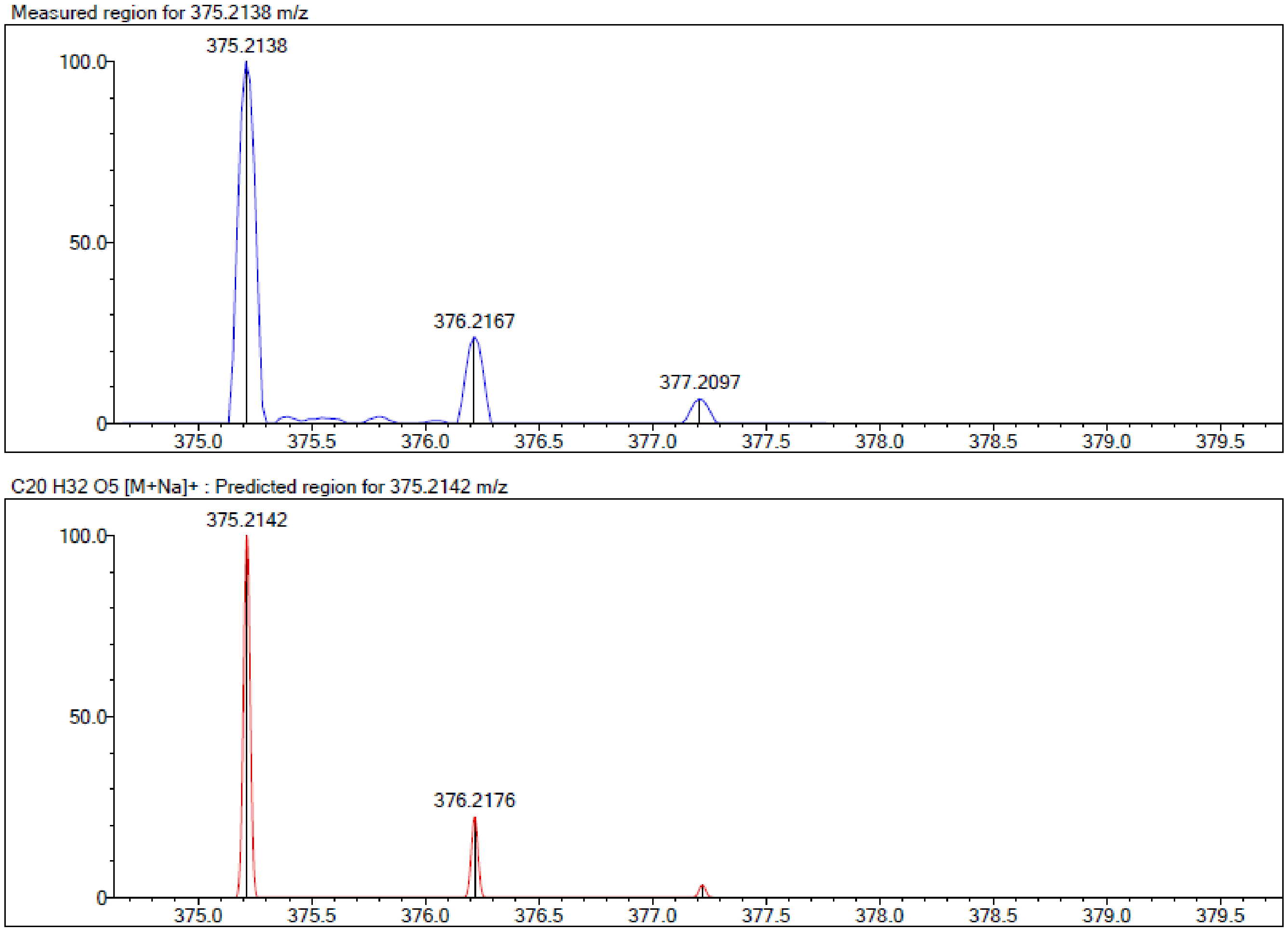

Rank	Score	Formula (M)	Ion	Meas. m/z	Pred. m/z	Df. (mDa)	Df. (ppm)	Iso	DBE
1	67.27	C20 H32 O5	[M+Na]+	375.2138	375.2142	-0.4	-1.07	67.38	5.0

S50 ^{1}H NMR spectrum of **8** in Methanol-d_4

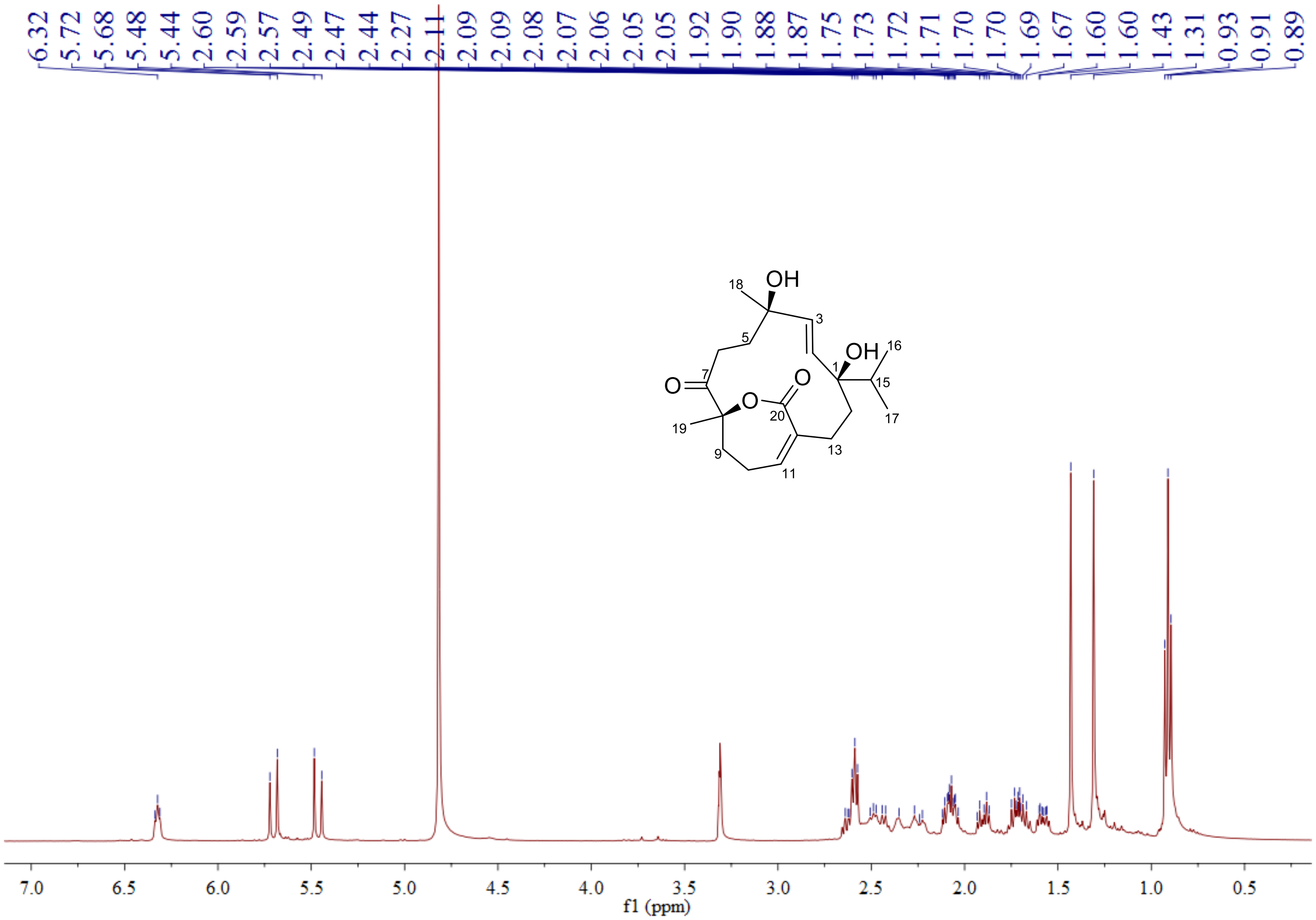

S51 ^{13}C NMR spectrum of **8** in Methanol-d_4

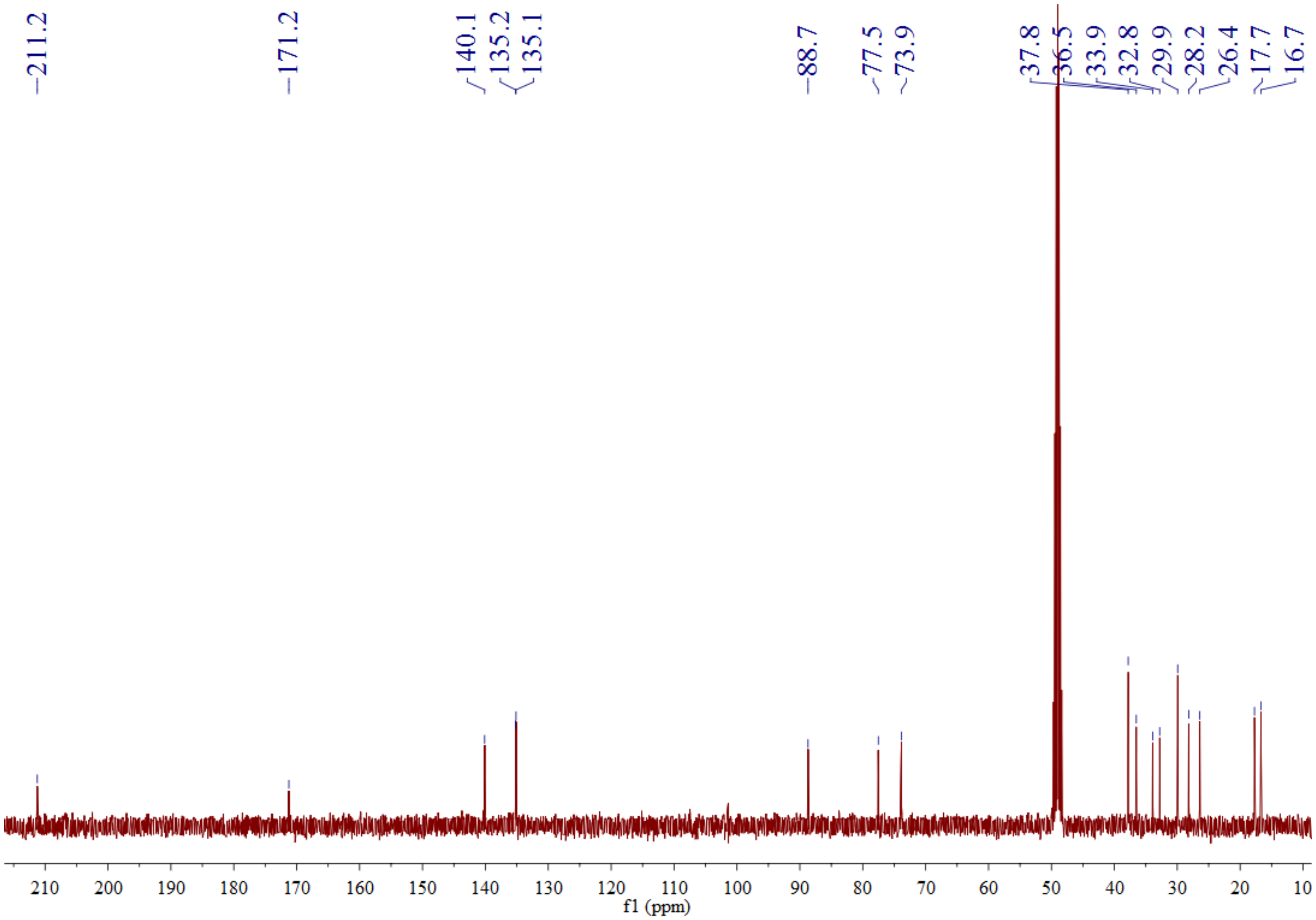

S52 ^{1}H NMR spectrum of **8** in $CDCl_3$

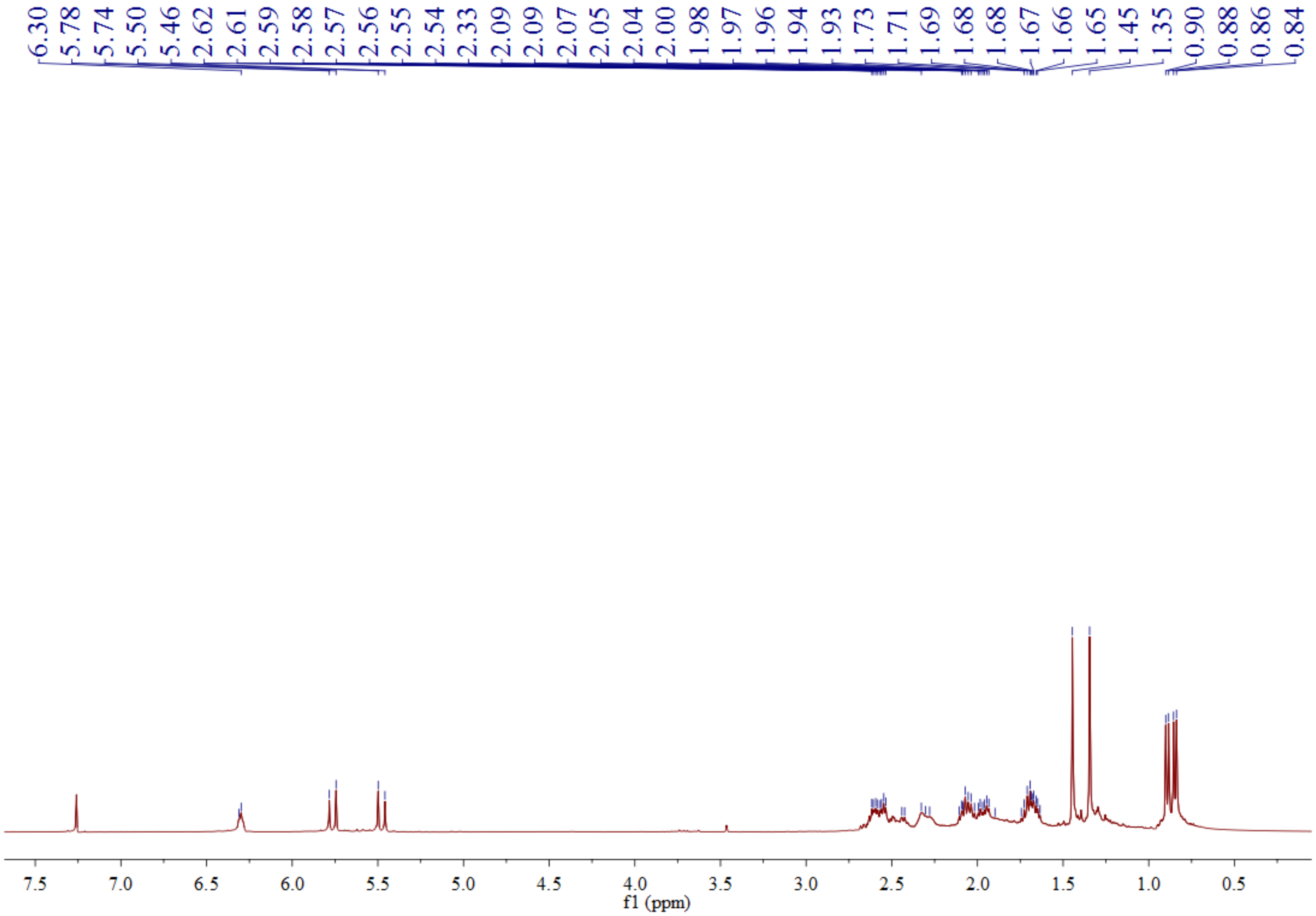

S53 ^{13}C NMR spectrum of **8** in $CDCl_3$

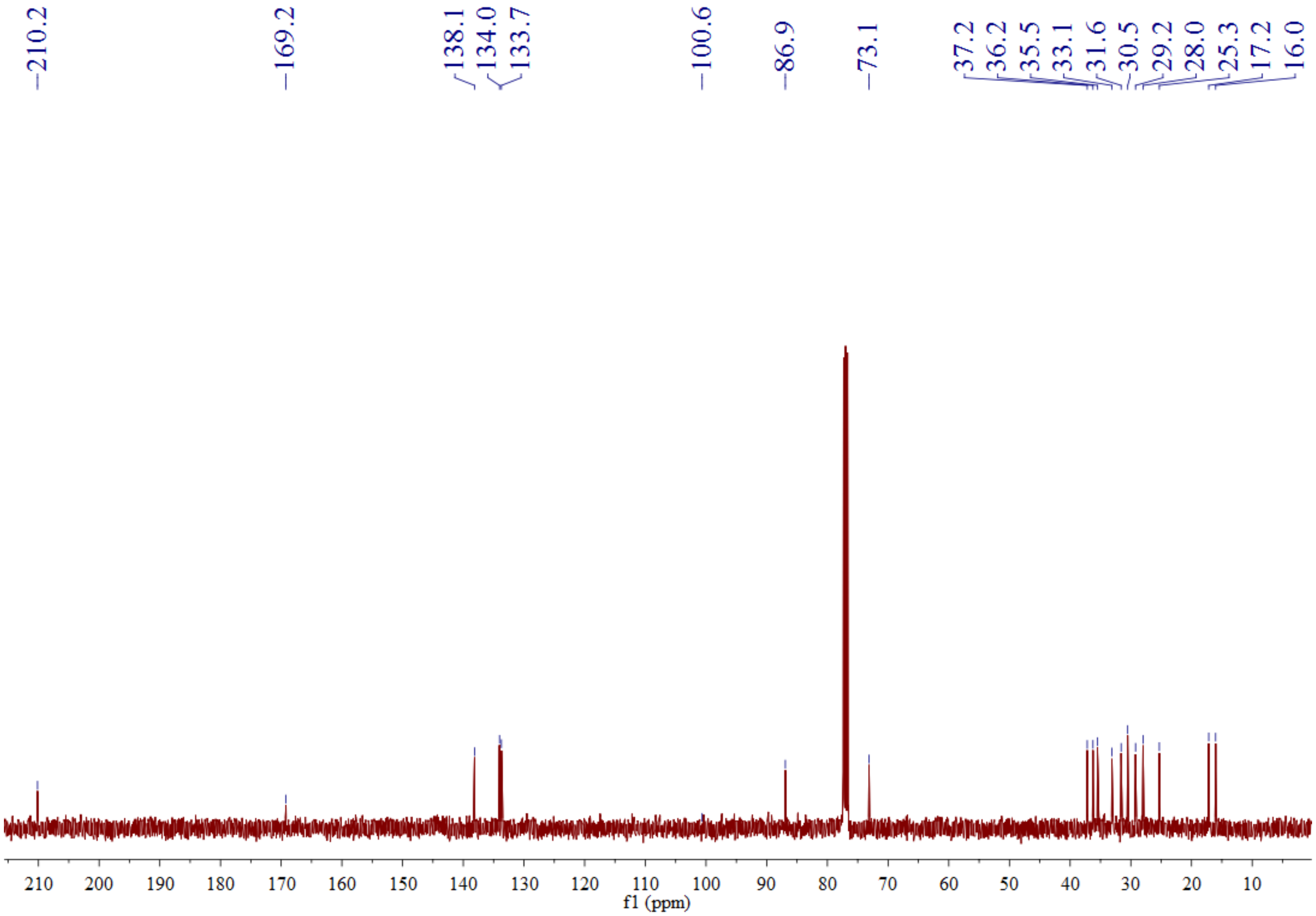

S54 ^{1}H NMR spectrum of **9** in Methanol-d_4

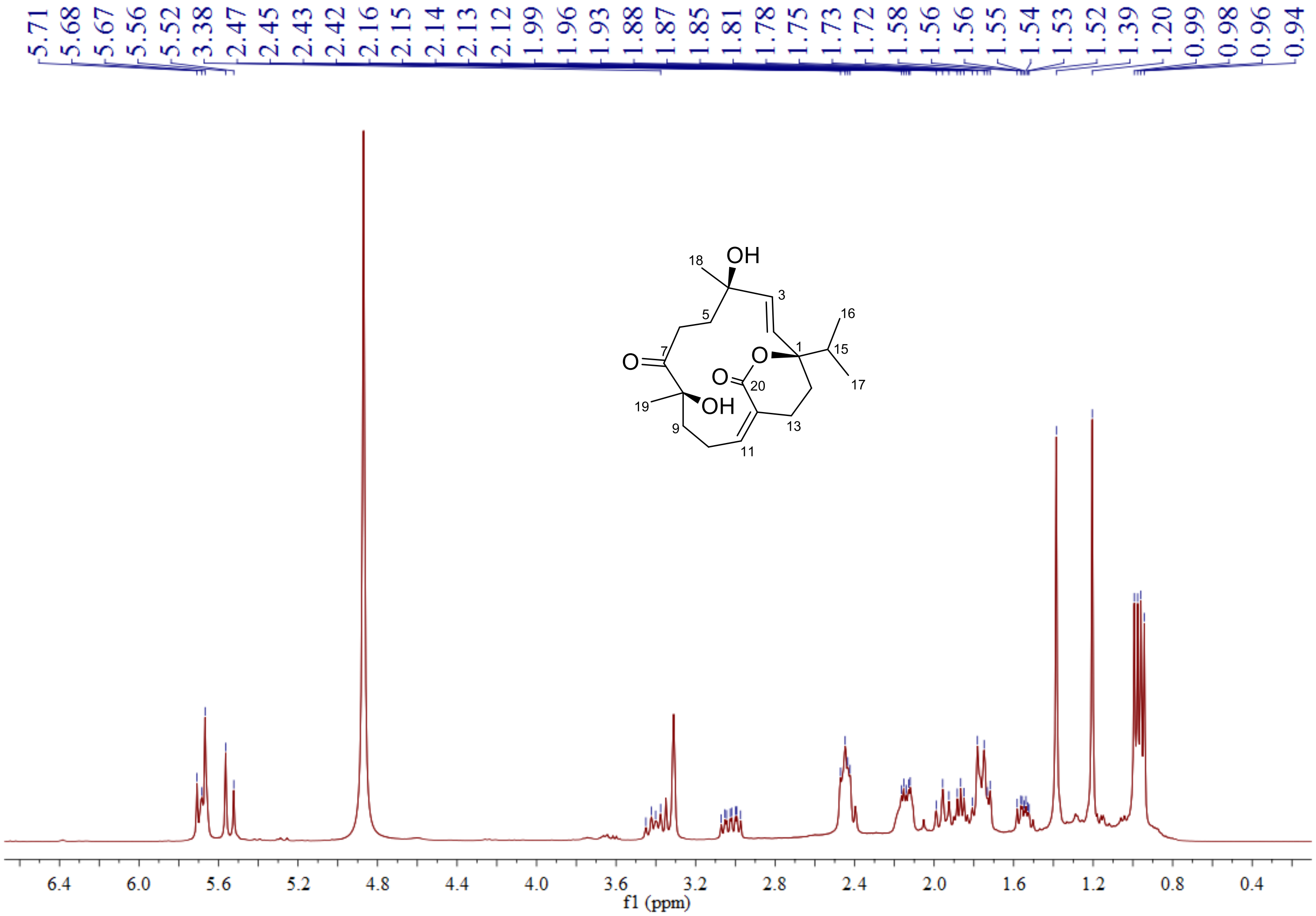

S55 ^{13}C NMR spectrum of **9** in Methanol-d_4

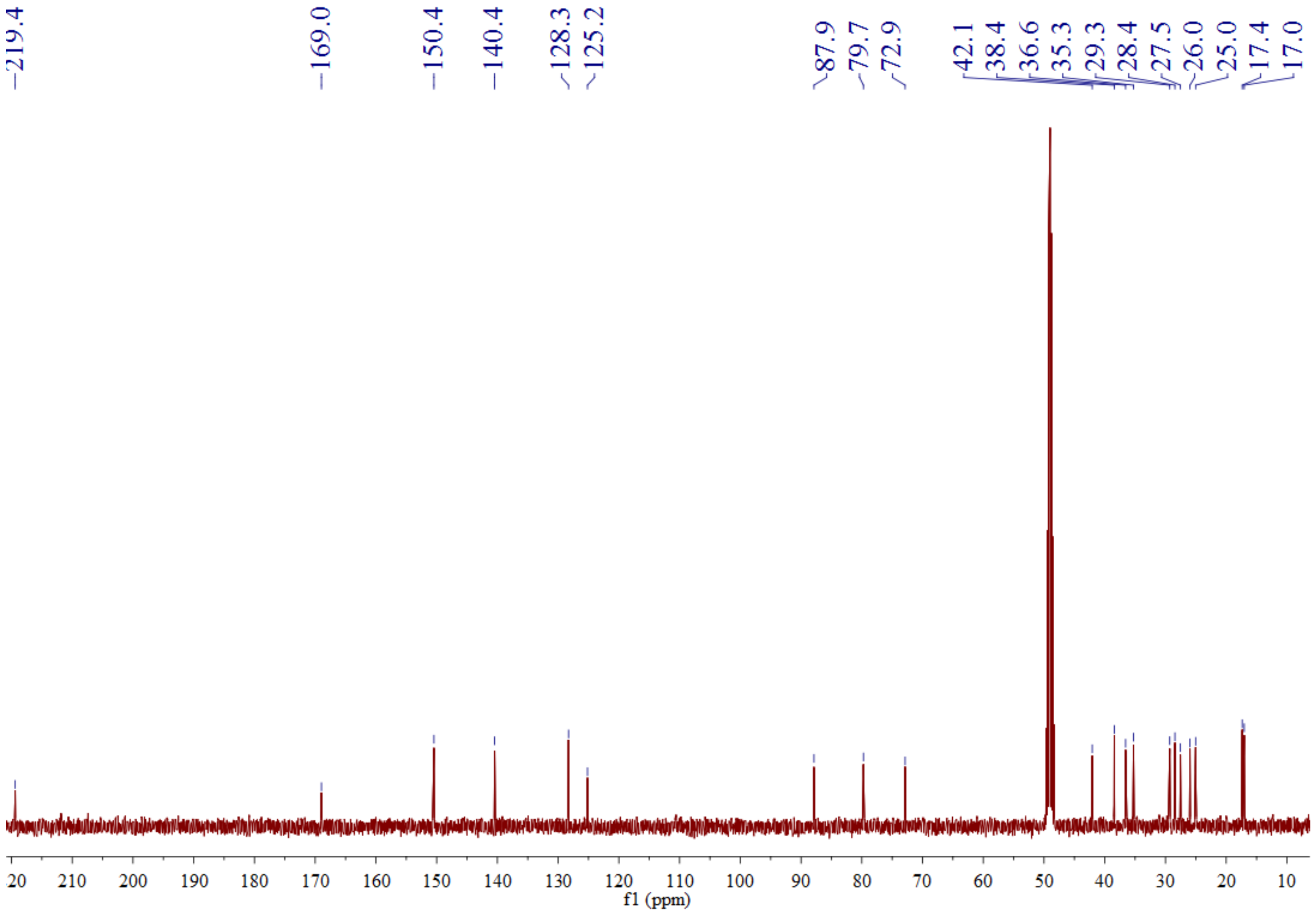

S56 Selected NOESY ( ) correlations of **2**.

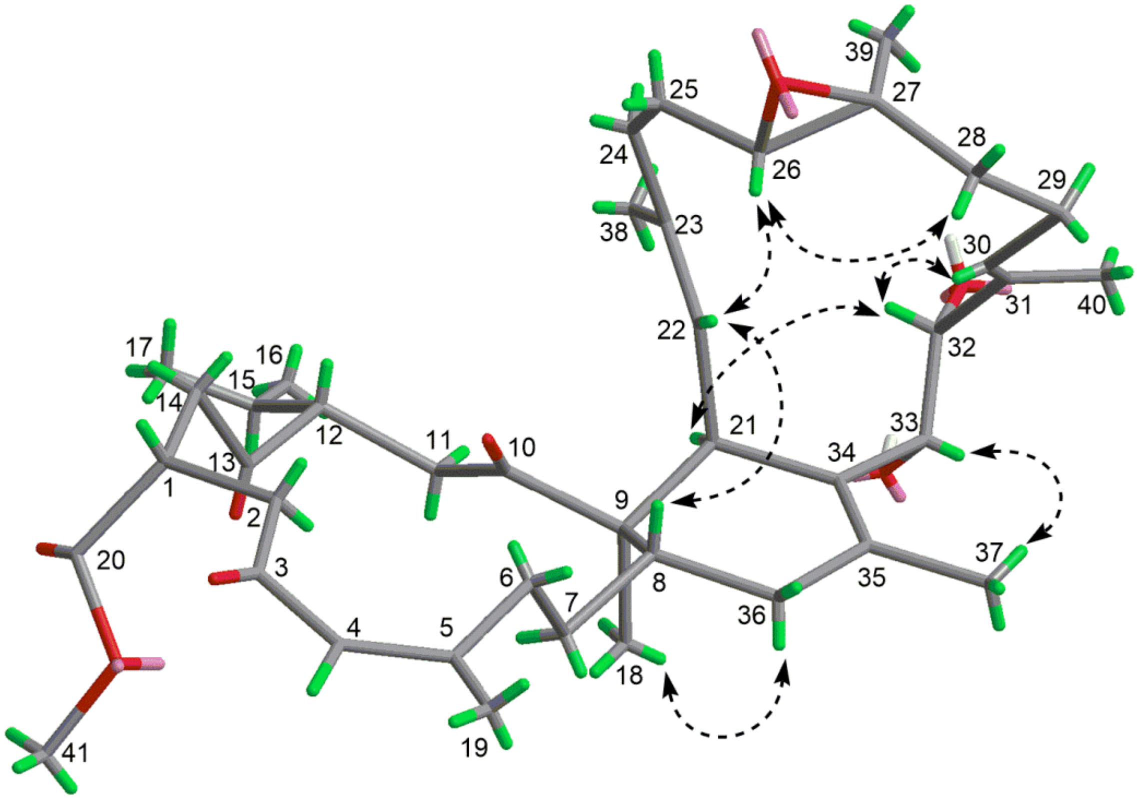

Supplement: Supplementary file 1 [file marinedrugs-15-00085-s001.pdf]
